# Multidisciplinary prediction of running-related injuries using machine learning

**DOI:** 10.1038/s41746-026-02413-y

**Published:** 2026-02-06

**Authors:** Han Wu, Katherine Brooke-Wavell, Michael R. Barnes, Zainab Awan, Sarabjit Mastana, Sam Allen, Richard C. Blagrove

**Affiliations:** 1 School of Sport, Exercise and Health Sciences, Loughborough University, Loughborough, UK; 2 Centre for Translational Bioinformatics, Queen Mary University of London, London, UK

**Keywords:** Computational biology and bioinformatics, Health care, Medical research, Risk factors

## Abstract

The causes of endurance running-related injury (RRI) are multifactorial, yet little research has been conducted which utilizes multidisciplinary risk factors for individualized RRI prediction. This paper presents a machine learning (ML)-ready RRI weekly prediction dataset using evidence-based multidisciplinary risk factors. Risk factors in genetic single-nucleotide polymorphisms, history, muscular strength, biomechanics, body composition, nutrition, and training were collected from competitive endurance runners (*n* = 142), who were prospectively monitored for 12 months for RRIs, accumulating 6181 weekly samples. ML models were fitted using (i) risk factors with high-level supporting evidence, and (ii) a broader range of risk factors to establish a performance baseline. Model performance (AUC = 0.784 ± 0.014) showed moderate improvement compared to previous RRI prediction modeling. Random forest achieved the best performance (AUC = 0.781 ± 0.016, 0.784 ± 0.014), which was significantly higher (*q* < 0.05) than most other algorithms. Only logistic regression achieved significantly improved (*q* < 0.05) performance when trained using a broader range of risk factors compared to a selection of high-quality risk factors. This study introduces a reproducible methodological framework for future ML sports injury prediction research and a valuable dataset for pooling in larger-scale analytics. Comparisons among different ML methods revealed nuanced insights into the interaction between data structure and model suitability.

## Introduction

Despite the considerable popularity and health-related benefits associated with endurance running, the activity has a high injury prevalence, estimated at ~45%^1^. Running-related injuries (RRIs) can have profound negative impacts upon quality of life and carry high financial burdens to participants and medical services^2^. The causes of RRIs are usually multifactorial and can be influenced by intrinsic characteristics (e.g., genetics, age, anthropometry), neuromuscular capabilities (i.e., muscular strength), previous injury, ground reaction force profile (i.e., biomechanics) during running, and training behaviors^3^. Although research in broader sports domains has considered the contribution of multidisciplinary risk factors in injury prediction modeling^4^, there has been an under-appreciation for the complex interplay of factors that cause RRI, with most studies examining risk factors from a unidimensional perspective^5^. For instance, single-nucleotide polymorphisms (SNPs) within the human genome affect proteins of various functions (e.g. hormone secretion, collagen formation/degradation) and form an integral part of an athlete’s injury risk profile^6^. Yet no research has combined genetic markers with other known multidisciplinary risk factors, specifically for RRI.

ML is currently used extensively in broader medical settings, specifically in diagnostics, prognostics, and precision medicine, which have application to sport injury research^7^. A recent scoping review noted numerous ML-based injury prediction studies have been published, yet the clinical efficacy of findings was limited by small cohort sizes, unclear definitions of injury, and vague reporting of measurements^8^. Injury predictor variables used as inputs in ML models should also be based upon well-established supporting evidence or have a strong theoretical basis^9^; however, previous studies utilized a broad range of independent variables (7–957), often without rationale^10–17^. Furthermore, previous studies have tended to use field-based or self-report measures as predictor variables, which lack internal validity compared to laboratory-based measures^10,11,14,16,17^. Accordingly, more research with high methodological quality that addresses ML’s unique strengths for injury prediction is warranted.

In previous studies that have used ML as a tool to predict sports injury, typically only a small number of predictive models have been applied^8^. This methodological approach limits the evaluation of ML techniques to predict injury in a cohort of athletes as more effective models are potentially omitted. Different ML models have been compared for performance in daily or weekly injury prediction across several sports^11–14,17^, yielding mixed results; however, a similar approach has not been conducted for the popular sport of endurance running. Consequently, there is a need to explore the comparative advantages of different ML algorithms in RRI prediction under varying data structures using robust measures that reflect well-established risk factors.

To-date, there is only one prospective study that has used ML to predict RRI in endurance runners^10^. Lövdal and co-workers used the XGBoost algorithm on training-related data (training logs, GPS, heart rate, perceived exertion) from 74 high-level endurance runners in the same team, reporting moderate ML performance with daily RRI prediction (AUC = 0.724), and low performance with weekly prediction (AUC = 0.678)^10^. Although these results show promise, the study included a small number of factors and lacked the insight that multivariable analysis of external and internal risk factors can potentially provide^10^. A recent study by Iatropoulos and colleagues explored the use of time-integrating feature engineering for training and sleep-related features in predicting weekly injuries among French track and field athletes (*n* = 165). The study employed logistic regression (LR), Support Vector Machines (SVMs), and several tree-based algorithms and achieved performance up to AUC = 0.82 with ensemble tree methods^17^. The comparative methodological approach to analyzing the performance of different ML models provided useful insight in this paper; however, the participants specialized in eight different event categories (i.e., sprints, jumps, throws, walking, running etc), with each category associated with injuries to different anatomical regions and varied injury types. Whether these ML methods retain their superiority in an endurance-runner-only cohort remains unknown.

The primary aim of this study was to create a multidisciplinary ML modeling dataset in a cohort of well-trained endurance runners utilizing a broad range of well-established risk factors for RRI in the areas of genetics, muscular strength, biomechanics, nutrition, body composition, anthropometry, and training. This methodology could serve as a reproducible framework for future attempts to predict RRIs or broader sports injuries using ML. Using this robust and multidisciplinary dataset, the secondary aim was to conduct a comparative analysis of the performance of different ML models for RRI prediction over a 12-month period.

## Results

### Participant and injury characteristics

**Fig. 1:**
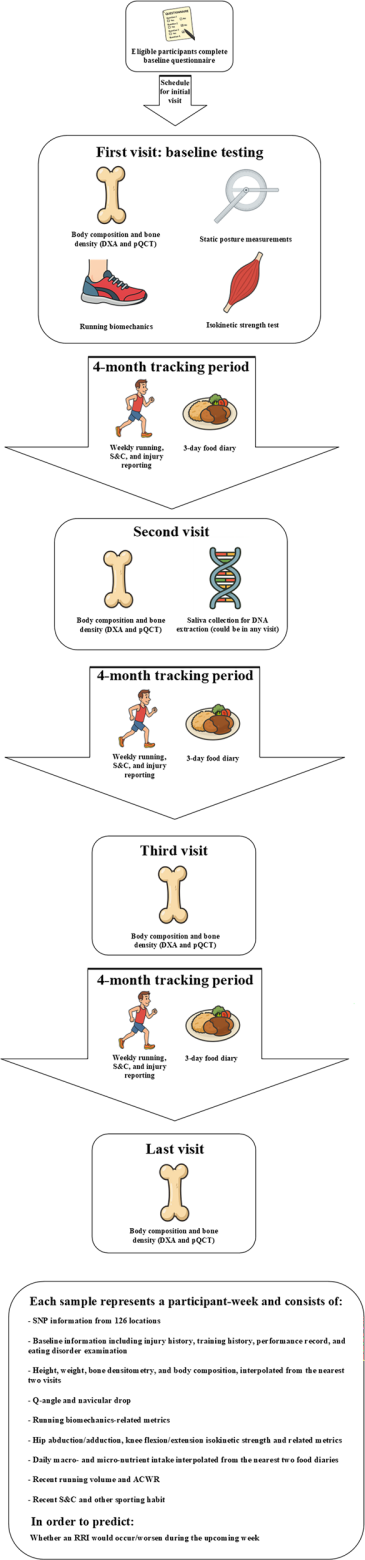
An overview of the data collection process. DXA dual x-ray absorptiometry, pQCT peripheral quantitative computed tomography, S&C strength and conditioning, SNP single-nucleotide polymorphism, Q quadriceps, ACWR acute:chronic workload ratio, RRI running-related injury. Figure was created using Microsoft Visio and cartoon images were generated using ChatGPT-5.1.

Figure 1 illustrates the data collection process. A total of 142 participants (female *n* = 64; male *n* = 78) contributed 6181 valid samples, including 564 recorded injury instances. World Athletics scores^18^ derived from six-month performance records averaged 811 ± 181 for females and 584 ± 245 for males. Participants reported 47.7 ± 91.4 days affected by lower limb injuries in the 12 months pre-baseline, during which 34 participants were uninjured. Among 607 identified injuries, knee (*n* = 122), Achilles tendon (*n* = 76), and hip (*n* = 73) were the most injured regions. Full details on participant adherence at each stage (Supplementary Fig. 1), descriptive statistics (Supplementary Table 1), and injury incidence by body location (Supplementary Table 2) are provided in Supplementary Material Section (SMS) 1.

### Model performance

**Fig. 2:**
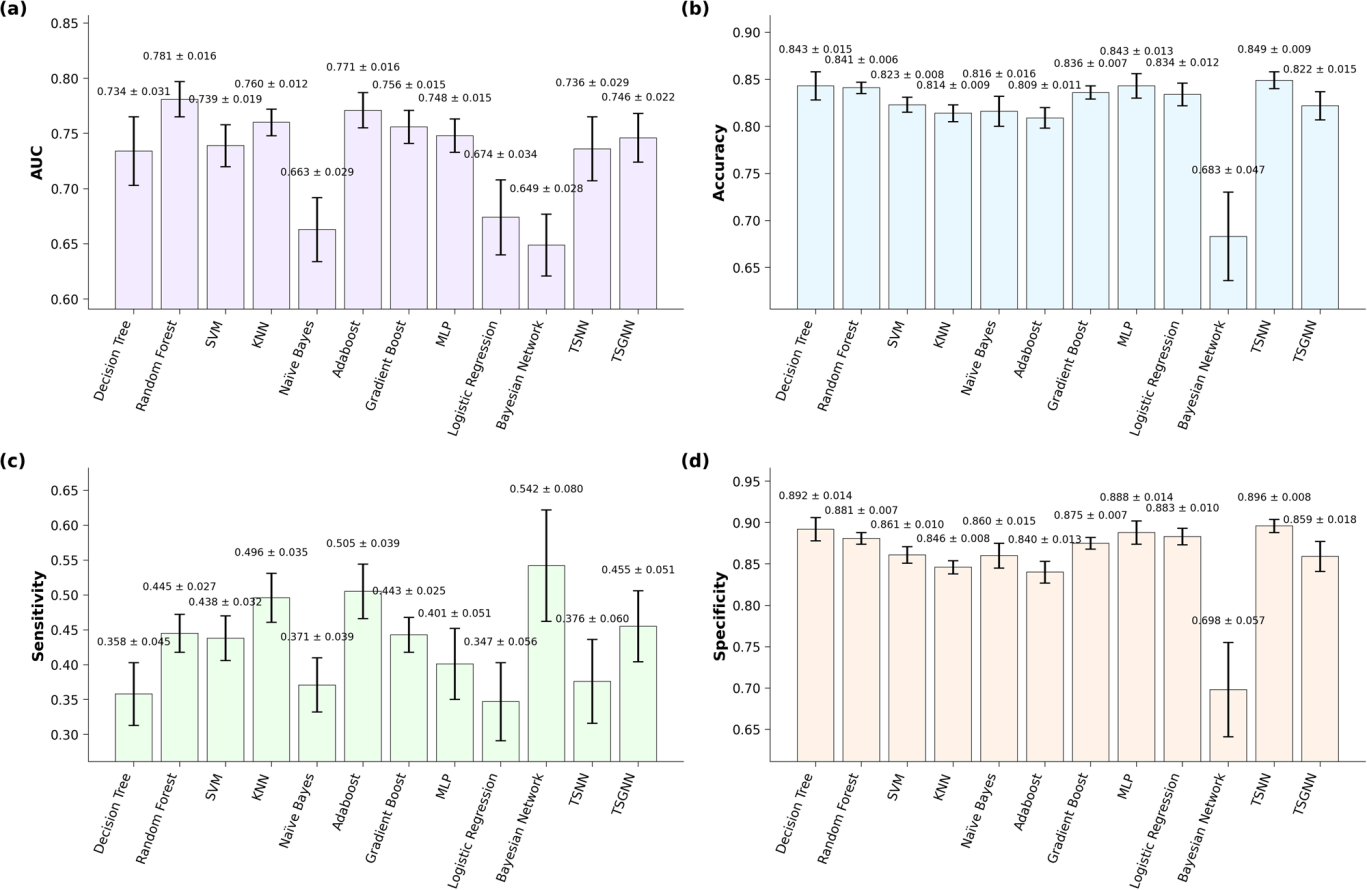
ML model performance for. **a** Area under the (receiver operating characteristic) curve (AUC), **b** accuracy, **c** sensitivity, and **d** specificity, when trained using only high-quality evidence-based risk factors (class 1 risk factors). Error bars denote 95% confidence interval. SVM support vector machine, KNN K-nearest neighbor, MLP multi-layer perceptron, TSNN time-sequenced neural network, TSGNN time-sequenced graph neural network. TSNN and TSGNN are novel algorithms. Figure was generated using matplotlib in python.

**Fig. 3:**
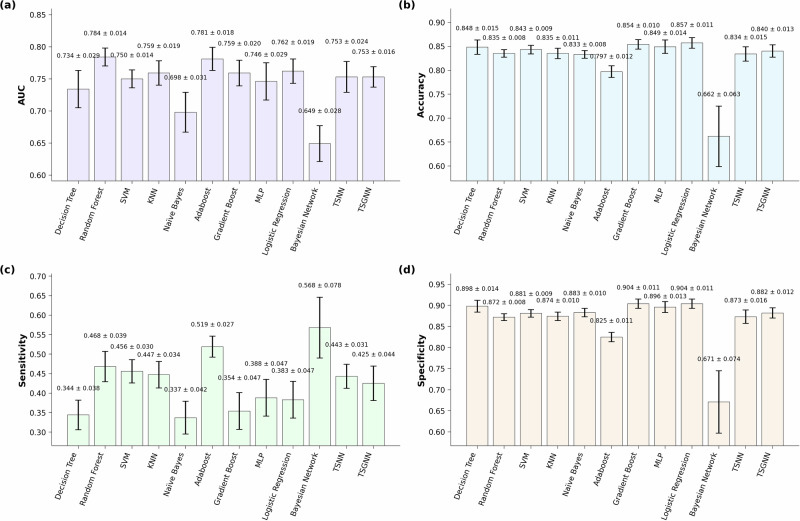
ML model performance for. **a** Area under the (receiver operating characteristic) curve (AUC), **b** accuracy, **c** sensitivity, and **d** specificity, when trained using all available risk factors. Error bars denote 95% confidence interval. SVM support vector machine. KNN K-nearest neighbor, MLP multi-layer perceptron, TSNN time-sequenced neural network, TSGNN time-sequenced graph neural network. TSNN and TSGNN are novel algorithms. Figure was generated using matplotlib in python.

Figures 2 and 3 show different ML models’ performance and corresponding 95% confidence intervals (CI) when trained using a selection of evidence-based risk factors (class 1 risk factors, *n* = 39) and using all available risk factors, which include those with weaker evidence (class 1–3, *n* = 257), respectively. Time-sequenced neural network (TSNN) and time-sequenced graph neural network (TSGNN) are novel algorithms designed to integrate domain-specific knowledge specifically suited for prognostic modeling and sports injury prediction; details can be found in the authors’ preprint publication^19^. Open-source code is available via GitHub page henrywu0709/TSNN-TSGNN-for-prognostic-modelling.

One-way analysis of variance (ANOVA) showed significant inter-model differences (F = 17.73, *p* < 0.001). Random forest achieved the best average AUC performance. When trained with class 1 feature set, its AUC (0.781 ± 0.016) was significantly higher than all other algorithms (*q* < 0.05) except Adaboost (AUC = 0.771 ± 0.016) in one-sided *t* tests. When trained using all features, its AUC (0.784 ± 0.014) remained higher than all algorithms (*q* < 0.05) except Adaboost (AUC = 0.781 ± 0.018) and logistic regression (LR, AUC = 0.762 ± 0.019). Naïve Bayes and Bayesian network were the lowest performing models. For class 1 feature set, their performance (AUC = 0.663 ± 0.029, 0.649 ± 0.028) were lower than all other methods (*q* < 0.05) except LR (AUC = 0.674 ± 0.034) in two-sided *t* tests. For the “all features” set, Bayesian network’s AUC remained low (0.649 ± 0.028); Naïve Bayes’ performance improved slightly (0.698 ± 0.031) but was still significantly lower than all except Bayesian network and decision tree (AUC = 0.734 ± 0.029). LR was the only ML method that significantly improved in AUC performance (*q* < 0.05) when trained using all features (0.762 ± 0.019) compared to only using class 1 features (0.674 ± 0.034). Detailed statistical analysis matrices showing all inter- and intra- algorithm comparisons are provided in Supplementary Tables 3–7 in SMS1.

**Fig. 4:**
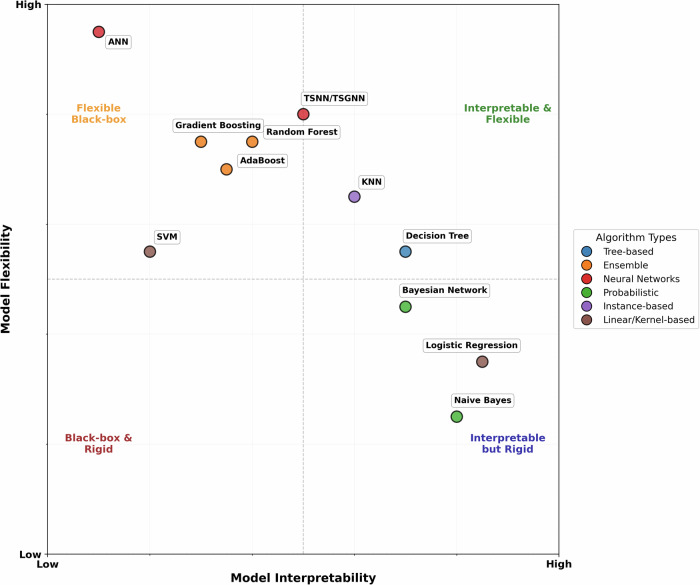
A general estimation of different ML methods’ flexibility and interpretability. Note that the graph is for general reference purposes and should not be used as a sole guide for algorithm selection. TSNN/TSGNN are placed at their expected relative position, subject to further validation on larger datasets. ANN artificial neural network (multi-layer perceptron is the basic form of ANN), SVM support vector machine, TSNN time-sequenced neural network, TSGNN time-sequenced graph neural network, KNN K-nearest neighbor. Figure was generated using matplotlib in python.

Based on the performance outcomes and to facilitate understanding of the relative merits and limitations of the ML algorithms, Fig. 4 shows an estimation of different models’ flexibility and interpretability.

## Discussion

This study collected RRI risk factor data from a wide range of relevant disciplines and conducted ML training using several existing and novel algorithms to compare performance. Its methodology serves as a valuable guide for future ML-based sports injury prediction research, and the results offer nuanced insights into how data structure affects ML model performance. Augmented data collection is warranted to fully achieve the models’ predictive potential, enable external validation, and facilitate translation to broader commercial settings.

This study’s highest AUC performance (Random Forest: 0.784 ± 0.014) is at a moderate level relative to those of previous weekly/daily sports injury prediction studies, which range from 0.62 to 0.84^10–17^. Specifically for the sport of endurance running, the current study showed a moderate improvement compared to Lövdal and colleagues who achieved AUC = 0.724 ± 0.01 for daily RRI prediction and AUC = 0.678 ± 0.01 for weekly RRI prediction^10^. Iatropoulos and co-workers predicted weekly athletics (track and field) injuries and their best-performing model reached AUC = 0.82 ± 0.01, which is slightly higher than the current study, although only a minority of participants specialized in distance running events^17^. Compared to previous research, the current study’s prediction task is particularly challenging given the heterogeneity of the participant cohort. Previous studies were conducted either within the same sports team/club/pathway^10,11,13–16^ or within the same league/federation^12,17^, and in some cases study participants were same sex^11–13,15^. These study design characteristics limit the range of participants’ age, competitive level, average training volume, and seasonal schedules. In contrast, the current study’s participants had a larger age range (14–50 years) and competed over a wider range of levels (county to International/elite level). Additionally, endurance running competitions vary significantly in distance (5 km to >100 km ultramarathon) and format (e.g., trail, road, track), which further increases heterogeneity of the stressors that cause injury. Consequently, while other studies’ prediction models primarily aim for internal use within a designated sports team or league, the current study targeted a broader setting and sport population that included all performance-oriented runners who train on a regular basis. To account for such increased variance among target samples, each sample needs to contain more relevant information to enable effective individualization, and more samples need to be available for sufficient learning. While this study introduces a high-quality multidisciplinary feature set that holistically captures current research understandings of RRI, its sample size (participants, *n* = 142; weekly samples *n* = 6181) remains insufficient due to logistical limitations, which likely explains its mediocre performance. Nevertheless, this study’s transparent and detailed reporting of methodology, data, and code could enable future research to conduct augmented data collection and pooled analysis, further improving practical applicability.

Most ML algorithms’ AUC performance remained unchanged when trained on all features compared to only using those features that have strong supporting evidence (class 1). The exception was LR (class 1 AUC = 0.674 ± 0.034, all features AUC = 0.762 ± 0.019), which improved significantly (*q* < 0.05) when trained using all features in a two-sided *t* test, elevating it from a bottom-three performer under the class 1 dataset to a top-three performer under the “all features” dataset. This is somewhat counterintuitive, as increased feature interrelationships typically favor ML models with higher flexibility over more rigid models^20^. Flexible ML models are better at capturing complex nonlinear interrelationships among features, and tend to perform better under large data size, complex tasks, and high parameterization (i.e., many features; Fig. 4). However, in the current dataset, only LR showed notable performance improvements when parameterization increased. A plausible explanation is that class 1 variables lacked directly correlated features, limiting LR’s performance, whereas more flexible ML models could extract indirect information. With an expanded feature pool, numerous directly linked features emerged, enhancing LR’s performance, while other models’ performances were constrained by limited training samples. Notably, only thirteen features overlapped between class 1 (21 features) and the full pool (95 features) for LR, indicating that some data in the larger pool was unavailable in the smaller pool. On this basis, there is a possibility that some class 2/3 candidate features were strong direct predictors of RRIs despite current weak evidence and thus warrant further investigation. Additionally, the contrast between LR and other ML models underscores that model performance is contingent on multiple factors, and in certain scenarios, simpler statistical models can rival complex models in performance, while offering greater interpretability^21^. Consequently, it is advisable to evaluate multiple models rather than assume superiority of specific models.

When comparing between ML methods, random forest (class 1 AUC = 0.781 ± 0.016, all features AUC = 0.784 ± 0.014) was significantly higher than all other algorithms in one-sided *t* tests (*q* < 0.05) except for Adaboost (class 1 AUC = 0.771 ± 0.016, all features AUC = 0.781 ± 0.018) and LR’s “all features” dataset (AUC = 0.762 ± 0.019). This aligns with Iatropoulos et al.^17^ whose best model performance was also achieved by random forest and Adaboost. A recent study comparing 14 different ML models and meta-models to predict performance in NBA players indicated that Random Forest, Bayesian Ridge, AdaBoost, and Elastic Net provided the most accurate ML results^22^. Additionally, a review article inspected the comparative use of different supervised ML algorithms for disease prediction and found that random forest had a higher chance of outperforming other methods^21^. Collectively, these findings suggest that random forest (and other ensemble tree methods) may have better structural compatibility with prognostic datasets and could be prioritized in future sports injury prediction studies.

Bayesian models (NB and Bayesian network) performed the worst and were significantly lower than all but LR in two-sided *t* tests (*q* < 0.05) when using the class 1 dataset (NB AUC = 0.663 ± 0.029, Bayesian network AUC = 0.649 ± 0.028). For the “all features” dataset, Bayesian network’s performance remained low (AUC = 0.649 ± 0.028), and although NB slightly improved in performance (AUC = 0.698 ± 0.031), it was still significantly lower than all other methods (*q* < 0.05) except decision tree (AUC = 0.734 ± 0.029). The application of Bayesian network required feature discretization, which likely resulted in a substantial loss of information since the current study contains many continuous features. NB builds on the assumption of conditional independence, thus any feature contingencies (e.g., feature ‘A’ only affects RRI given the presence of feature ‘B’) or inter-feature correlation could detriment NB’s performance^23^. During feature construction, many risk factors that were thought to highly correlate with existing features were included in class 3, which likely explains why NB’s performance did not improve as much as LR when employing the “all features” dataset.

An important limitation of the current study is the lack of external validation on a separate cohort of runners, which reduces its generalizability. Additionally, the small participant number (*n* = 142), although twice as large compared to previous research^10^, is insufficient to capture the large variance among the target cohort, potentially resulting in suboptimal model performance. Consequently, the models are not yet ready for clinical application and first requires validation on larger independent cohorts.

Although participants were required to be uninjured at baseline and engaging in habitual running training, the nature of an injury prediction study may have attracted injury-prone participants, potentially introducing selection bias. Participant recruitment for prospective injury monitoring studies is also prone to ‘survivorship bias’. This type of selection bias describes how, at population level, runners who have suffered severe or recurring injuries and dropped out of the sport will not form part of the eligible participant pool. Older participants are therefore potentially prone to longer periods of survivorship bias compared to younger participants and could thus be subject to relatively lower RRI risk at baseline.

In terms of data collection, RRIs were self-reported via questionnaire, which may have introduced reporting bias. This was somewhat alleviated as the reported injury cases were individually inspected during data curation, and any uncertainties were confirmed via direct correspondence with participants. Due to logistical constraints, the temporal resolutions (i.e., frequency of data collection) of some features (e.g., nutrition-related features) were low, potentially resulting in suboptimal model performance.

Regarding ML training, different algorithms employed varied computational loads. An algorithm with low computational load allowed for more hyperparameter combinations to be tested without overburdening the budget of the study. This may have led to higher chances of optimal performance in these models, which could bias the comparative results. This is particularly true for TSNN and TSGNN. Since they were newly designed models that were not yet optimized for computation, their practical testing iterations were severely limited compared to other ML methods. For the same reason, some hyperparameters (e.g., attention weights, batch normalization, oversampling) were fixed for TSNN and TSGNN to facilitate feature number and learning rate exploration^19^, possibly overlooking superior hyperparameter configurations. Finally, feature ranking was performed on the entire dataset prior to cross-validation to ensure a consistent feature set across folds. While pragmatic, this approach introduces a risk of data leakage and potential overfitting.

The long-term goal of this research is to predict RRI risk using a decision-support tool (e.g., a mobile application) that provides an individualized risk estimate to performance-oriented runners. A user would receive a one-time genetic sequencing, complete an annual test battery (including strength assessment, running biomechanics, bone scans, and static posture), upload regular food diaries, and report weekly training and injuries. The application would be capable of providing the user with accurate daily advice on workload prescription and injury prevention, which would inform training and lifestyle decision-making.

Future studies should conduct larger-scale data collection to enable external validation and pooled analysis. Subsequently, it is recommended that randomized controlled trials (RCTs) are carried out to determine if ML-derived feedback can effectively reduce RRI incidence. Demonstrated efficacy in an RCT is a prerequisite for clinical adoption and further development.

Several current or upcoming technologies could be integrated into the data collection process to improve convenience and accuracy. For instance, many commercially available electronic watches contain GPS-based running tracking and photoplethysmography-based heart rate monitoring that can be used instead of weekly self-reporting to track external and internal workload. These watches can also estimate sleep duration and quality, which could be valuable indicators of real-time physiological stress^24,25^. Recent research suggests that lab-based biomechanics measurements could potentially be calculated from wearable inertial measurement units and pressure insole data through supervised ML^26^. Furthermore, ML-based image recognition could potentially be applied to calculate nutrient intake directly from images of food^27^. These advancements could vastly improve the temporal resolution and convenience of data collection (i.e., compared to a 3-day food diary every four months).

Feature importance analysis (e.g., Shapley Additive Explanations) could be used in a follow-up study to explain the trained models, and the results compared against traditional univariate statistical analysis to inspect whether ML-based explainability provides additional insights. Longitudinal data (i.e., data collected weekly) could also be further explored on the feature-engineering level^17^. This could not only improve model performance but also enable longitudinal explainability via feature importance analysis. Causal inference methodologies (e.g., meta-learners) could be applied to investigate whether modifiable risk factors (e.g., muscular strength) contribute to increased or decreased RRI risk on an individual level.

## Methods

### Ethics and participant recruitment

This study employed a prospective cohort design examining risk factors associated with RRIs over a 1-year period in 149 endurance runners. It received approval from the National Health Service (NHS) Research Ethics Committee (South West - Central Bristol) and the Loughborough University Ethics Sub-Committee. Participants were recruited between November 2022 and July 2023. Inclusion criteria were: (i) age 14–50 years, (ii) participate in competitive endurance (≥5 km) running for at least three years, (iii) minimum four hours running per week, and (iv) absence of injuries at initiation. Exclusion criteria were: (i) use of medications or presence of medical conditions significantly affecting bone health, (ii) pins or plates in limbs subjected to bone scans, (iii) pregnant or breastfeeding within previous six months, and (iv) current use of vaping or smoking. Recruitment was conducted via social media and direct contact with running clubs and coaches across the UK. The study was explained to each participant, and signed informed consent was obtained. For participants under 18 years old, assent from the participant and consent from their parent/carer were obtained.

### Baseline measurements

Due to the high number of potential predictor variables and associated measurement protocols, detailed procedures are included in the supplementary material (SMS2-3). ML features, which represent potential RRI risk factors, were categorized into three classes based on quality of associated evidence (SMS4). Class 1 risk factors possessed the highest evidence level (prospective evidence for non-genetic variables; strong mechanistic support plus associative evidence for genetic variables; SMS4) and included the following: sex^28^, age^29^, days injured during prior 12 months^30^, average weekly running hours and interval training frequency during prior 12 months^31^, score for the Eating Disorder Examination Questionnaire (EDE-Q)^32^, hip abduction peak torque^33^, ratio between hip abduction/adduction strengths^34^, knee extension and flexion peak torque^33^, navicular drop and asymmetry^30,35^, Quadriceps (Q) angle and asymmetry^36^, vertical average loading rate^37^, impact peak force^37^, duty factor^38^, daily fat intake^39^, body mass index (BMI)^30^, bone mineral density for anterior-posterior lumbar spine^32^, running distance during previous month^40^, ACWR, calculated as last 7-days running volume divided by the 28-days running volume prior^41^, average weekly S&C volume and non-running exercise volume during past 3 months^42^, prior injuries during prospective tracking period^30^, Single-Nucleotide Polymorphisms (SNPs) rs11225395^43^, rs1144393^43^, rs650108^44^, rs679620^44,45^, rs2252070^43^, rs4986938^46^, rs1800012^47^, rs4789932^45^, rs9340799^48^, rs970547^49^, rs1800795^50^, rs13946^51^, rs12722^50,51^, and total risk score combining all class1 SNPs.

Participants completed an online questionnaire (Onlinesurveys, Jisc, Bristol, UK) encompassing 12-month injury history, Bone-specific Physical Activity Questionnaire (BPAQ)^52^, six-month performance records, 12-month training history, EDE-Q^53^, and Low Energy Availability in Females Questionnaire (LEAF-Q)^54^. On arrival at the laboratory, participants’ height and body mass were measured (Seca 274 stadiometer, Birmingham, UK). Dual-energy X-ray Absorptiometry (DXA) scans (GE Lunar iDXA, GE HealthCare Technologies, Chicago, US) were used to measure bone density of the lumbar spine, hips and whole body, plus fat free mass of the leg region. A Peripheral Quantitative Computed Tomography (pQCT) scan (XCT 2000L, Novotec Medical GmbH and Stratec Medizintechnik GmbH, Pforzheim, Germany) was conducted for the non-dominant tibia at 66% tibial length to measure muscle cross-sectional area. Q-angle was measured using a goniometer, and sit-to-stand navicular drop was assessed using an established protocol^55^. Following a 5 min warm-up, running vertical ground reaction forces and stride frequency were recorded on an instrumented treadmill (Treadmetrix, Utah, US) at 10km/h and 12 km/h, each for one minute at 1000 Hz sampling frequency. Concentric hip abduction/adduction and knee flexion/extension peak torques were measured using an isokinetic dynamometer (Isomed 2000, D&R Ferstl GmbH, Hemau, Germany) at 60 degrees/sec and 200 Hz sampling frequency, over five sets of four repetitions as indicators of muscular strength.

### Prospective tracking period

Following baseline testing, participants completed a weekly online questionnaire (Qualtrics XM, Seattle, US) for 52 weeks, reporting running volume, other physical training, and injuries using the Oslo Sports Trauma Research Centre Overuse Injury Questionnaire^56^. Every four months, participants revisited the laboratory for DXA and pQCT scans, replicating baseline procedures. Between visits, participants maintained a three-day food diary using Libro App (Nutritics, Dublin, Ireland) with standardized digital weighing scales (Duratool, Farnell; Leeds, UK) provided.

Participants provided 2 ml of saliva (Isohelix GeneFix™ Saliva-Prep 2 DNA Kit, Cell Projects, Harrietsham, UK) during a visit for DNA extraction, which was subsequently analyzed by CD Genomics (New York, US) for selected SNPs previously correlated with RRIs.

### Data preprocessing

Each participant-week constituted a sample, with the objective of utilizing available information preceding each week to predict injury occurrence within a given week. Injury was defined as an increase in Oslo Questionnaire score for any bodily region compared to the prior week plus the participant’s subjective identification of the injury being running-related^56^. Samples lacking prior week data were excluded and injury cases were individually inspected to reduce errors. Analyses were performed separately for class 1 (*n* = 39) and all features (class 1–3; *n* = 257).

All data was normalized to 0–1 for ML training. All participants completed the baseline questionnaire and the initial testing session, so data imputation was not needed for these risk factors. However, the biomechanics laboratory experienced a power outage during an afternoon of initial testing, and three participants’ data collection was affected. Two participants conducted the testing in their next visit, and one dropped out before conducting the next visit, resulting in missed biomechanics data for one participant, which was imputed using the median value from all other participants of the same sex. Running biomechanics included variables related to vertical loading rate, which can only be calculated from rearfoot strikers (defined as participants who produced two vGRF peaks within the landing phase in >70% of their steps^57^). For non-rearfoot strikers, features relevant to vertical loading rate were imputed with 0. Missing SNP results were imputed using the mean of all available participants’ values to reflect the average genotypic expectancy of the target cohort. Missing nutritional values were imputed using the median of all available participants’ calculated daily intake. For weekly tracking data, missing values would constitute excluded samples, so all data was available in the curated dataset.

### Model selection

ML algorithms commonly used in previous sports injury prevention and medical prognostic modeling research were selected for comparison to identify model(s) with the best performance and interpretability^8,58^. This selection ranged from simple, more transparent models (Decision Tree, LR, Bayesian Networks) to complex models with different mathematical assumptions, including tree-based ensemble methods (Random Forest, Adaboost, Gradient Boosting), Support Vector Machine (SVM), K-nearest neighbor (KNN), and Artificial Neural Network (ANN). Additionally, two novel ML algorithms, Time-Sequenced Neural Network (TSNN) and Time-Sequenced Graph Neural Network (TSGNN) were designed and tested^19^. These novel algorithms integrate temporal domain-specific logic that reflects the progressive nature of human risk exposure, and possess the potential to achieve better interpretability specifically for prognostic modeling^19^.

### Model evaluation

AUC for the Receiver Operating Characteristic within a stratified 10-fold cross-validation framework was employed as the evaluation metric to prevent overfitting and for comparison against previous ML RRI studies^10,59^. Each fold divides the dataset into a 90% training set and a 10% testing set with the same injured-to-uninjured ratio. Due to high candidate feature-to-sample ratio, Relief and logistic Least Absolute Shrinkage and Selection Operator (LASSO) were used to reduce dimensionality. Relief is not constrained by sample distribution assumptions but can be severely affected by inter-feature correlations^60^. In contrast, LASSO handles correlated features by selecting only the most influential feature but is heavily dependent on model-based mathematical assumptions^61^. Consequently, the two methods were employed in parallel.

Relief was first applied to the entire dataset to rank features by importance. An algorithm was subsequently trained and tested via stratified 10-fold cross-validation using scikit-learn default hyperparameters and the top-ranked feature, the top 1 + 2 features, the top 1 + 2 + 3 features, and so on. Consequently, the number of feature subsets tested equals the total number of features within the feature pool. The feature subset with the highest average AUC was selected for each algorithm.

For LASSO, 40 equally spaced alpha values (0.1–0.00001) were employed to rank features separately, followed by iterative testing as performed in relief. Since LASSO may reduce unimportant features’ score to 0, the number of subsets eventually tested ≤40*number of features in the pool, and the best-performing feature subset was selected. During application, only decision tree and Bayesian network presented with situations where relief and LASSO yielded large performance differences, in which case only the better-performing feature subset was taken forward to hyperparameter tuning. For cases where relief and LASSO yielded two subsets that performed similarly, both feature subsets went through hyperparameter tuning.

Hyperparameter tuning also used average AUC from stratified 10-fold cross-validation as the evaluation metric. Grid search was employed for all commonly used hyperparameters (SMS5) to enhance robustness. Oversampling strategies and sampling rates were concurrently tuned to address class imbalance. If optimal hyperparameters reached the boundaries of specified ranges during the initial trial, the ranges were expanded, and a narrowed search was conducted until all hyperparameters resided within boundaries. The novel algorithms possessed unique structures which necessitated a different approach that merges dimensionality reduction and hyperparameter tuning^19^. Following hyperparameter tuning, models were retrained using optimal hyperparameters to determine a threshold that maximizes the f1 score. Accuracy, sensitivity, and specificity were reported under this threshold. For each performance metric, mean and 95% CI were reported. One-way ANOVA was conducted, and post-hoc one- and two-sided independent *t* tests were performed to inspect significant differences between different ML methods and between different feature sets. The Benjamini–Hochberg procedure was used to control false discovery rate.

## Supplementary information

Supplementary information

Supplementary Data1

Supplementary Data2

## Data Availability

Processed data is shared within the supplementary materials. Raw data is available upon contact, however some metrics (e.g. past performance records) need to remain normalized to ensure participant anonymity.

## Code availability

Code is available via GitHub page: henrywu0709/TSNN-TSGNN-for-prognostic-modelling.

## References

[1] Kakouris, N., Yener, N. & Fong, D. T. P. A systematic review of running-related musculoskeletal injuries in runners. *J. Sport Health Sci.* **10**, 513–522 (2021). 33862272 10.1016/j.jshs.2021.04.001 PMC8500811

[2] Hespanhol Junior, L. C., van Mechelen, W., Postuma, E. & Verhagen, E. Health and economic burden of running-related injuries in runners training for an event: a prospective cohort study. *Scand. J. Med. Sci. Sports* **26**, 1091–1099 (2016). 26282068 10.1111/sms.12541

[3] Winter, S. C., Gordon, S., Brice, S. M., Lindsay, D. & Barrs, S. A multifactorial approach to overuse running injuries: a 1-year prospective study. *Sports Health.* **12**, 296–303 (2020). 31994970 10.1177/1941738119888504 PMC7222667

[4] Bittencourt, N. F. N. et al. Complex systems approach for sports injuries: moving from risk factor identification to injury pattern recognition—narrative review and new concept. *Br. J. Sports Med.* **50**, 1309–1314 (2016). 27445362 10.1136/bjsports-2015-095850

[5] Correia, C. K. et al. Risk factors for running-related injuries: an umbrella systematic review. *J. Sport Health Sci.* **13**, 793–804 (2024). 38697289 10.1016/j.jshs.2024.04.011 PMC11336318

[6] Maffulli, N. et al. The genetics of sports injuries and athletic performance. *Muscles Ligaments Tendons J.* **3**, 173–189 (2013). 24367777 PMC3838326

[7] Naik, K. et al. Current status and future directions: the application of artificial intelligence/machine learning for precision medicine. *Clin. Pharm. Ther.* **115**, 673–686 (2024). 10.1002/cpt.3152 38103204

[8] Leckey, C., van Dyk, N., Doherty, C., Lawlor, A. & Delahunt, E. Machine learning approaches to injury risk prediction in sport: a scoping review with evidence synthesis. *Br. J. Sports Med.* **59**, 491–500 (2025). 39613453 10.1136/bjsports-2024-108576 PMC12013557

[9] Edouard, P., Verhagen, E. & Navarro, L. Machine learning analyses can be of interest to estimate the risk of injury in sports injury and rehabilitation. *Ann. Phys. Rehabil. Med.* **65**, 101431 (2022). 32871283 10.1016/j.rehab.2020.07.012

[10] Lövdal, S. S., Den Hartigh, R. J. R. & Azzopardi, G. Injury prediction in competitive runners with machine learning. *Int. J. Sports Physiol. Perform.* **16**, 1522–1531 (2021). 33931574 10.1123/ijspp.2020-0518

[11] Rossi, A. et al. Effective injury forecasting in soccer with GPS training data and machine learning. *PLoS One* **13**, e0201264 (2018). 30044858 10.1371/journal.pone.0201264 PMC6059460

[12] Hecksteden, A. et al. Forecasting football injuries by combining screening, monitoring and machine learning. *Sci. Med. Footb.* **7**, 214–228 (2023). 35757889 10.1080/24733938.2022.2095006

[13] Mandorino, M., Figueiredo, A. J., Cima, G. & Tessitore, A. Predictive analytic techniques to identify hidden relationships between training load, fatigue and muscle strains in young soccer players. *Sports* **10**, 3 (2021). 35050968 10.3390/sports10010003 PMC8822888

[14] Carey, D. L. et al. Predictive modelling of training loads and injury in Australian football. *Int. J. Comput Sci. Sport* **17**, 49–66 (2018).

[15] Goggins, L. et al. Detecting injury risk factors with algorithmic models in elite women’s pathway cricket. *Int. J. Sports Med.* **43**, 344–349 (2022). 34560790 10.1055/a-1502-6824

[16] Thornton, H. R., Delaney, J. A., Duthie, G. M. & Dascombe, B. J. Importance of various training-load measures in injury incidence of professional rugby league athletes. *Int. J. Sports Physiol. Perform.* **12**, 819–824 (2017). 27918659 10.1123/ijspp.2016-0326

[17] Iatropoulos, S., Dandrieux, P.-E., Edouard, P. & Navarro, L. Development and internal validation of machine learning prognostic models of sports injuries using self-reported data in athletics (track and field): The influence of quantity and quality of features. *J. Sports Sci.* 1–15, 10.1080/02640414.2025.2517971 (2025). 10.1080/02640414.2025.2517971 40509963

[18] Spiriev, B., Spiriev, A. *World Athletics Scoring Tables of Athletics.* https://worldathletics.org/about-iaaf/documents/technical-information (2022).

[19] Wu, H. et al. A time-sequenced approach to machine learning prognostic modelling with implementation on running-related injury prediction. Preprint at 10.1101/2025.05.07.25327162 (2025).

[20] Steyerberg, E. W., van der Ploeg, T. & Van Calster, B. Risk prediction with machine learning and regression methods. *Biometrical J.* **56**, 601–606 (2014). 10.1002/bimj.201300297 24615859

[21] Uddin, S., Khan, A., Hossain, M. E. & Moni, M. A. Comparing different supervised machine learning algorithms for disease prediction. *BMC Med. Inf. Decis. Mak.* **19**, 281 (2019). 10.1186/s12911-019-1004-8 PMC6925840 31864346

[22] Papageorgiou, G., Sarlis, V. & Tjortjis, C. An innovative method for accurate NBA player performance forecasting and line-up optimization in daily fantasy sports. *Int. J. Data Sci. Anal.* **20**, 1215–1238 (2025).

[23] Yang, F. An implementation of naive bayes classifier. In *2018 International Conference on Computational Science and Computational Intelligence (CSCI**)* 301–306 (IEEE, 2018). 10.1109/CSCI46756.2018.00065.

[24] Senbel, S. et al. Impact of sleep and training on game performance and injury in division-1 women’s basketball amidst the pandemic. *IEEE Access* **10**, 15516–15527 (2022).

[25] Goldberg, M. et al. Poor sleep quality is associated with an increased risk of running-related injuries: a prospective study of 339 runners over six months. *Scand. J. Med. Sci. Sports* **35**, e70164 (2025). 10.1111/sms.70164 41239840

[26] Xiang, L. et al. Recent machine learning progress in lower limb running biomechanics with wearable technology: a systematic review. *Front. Neurorobot.* **16**, 913052 (2022). 10.3389/fnbot.2022.913052 PMC9201717 35721274

[27] Theodore Armand, T. P., Nfor, K. A., Kim, J.-I. & Kim, H.-C. Applications of artificial intelligence, machine learning, and deep learning in nutrition: a systematic review. *Nutrients* **16**, 1073 (2024). 38613106 10.3390/nu16071073 PMC11013624

[28] Messier, S. P. et al. A 2-year prospective cohort study of overuse running injuries: the runners and injury longitudinal study (TRAILS). *Am. J. Sports Med.* **46**, 2211–2221 (2018). 29791183 10.1177/0363546518773755

[29] Nielsen, R. O. et al. Predictors of running-related injuries among 930 novice runners: a 1-year prospective follow-up study. *Orthop. J. Sports Med.* **1**, 2325967113487316 (2013). 10.1177/2325967113487316 PMC4555503 26535228

[30] Buist, I., Bredeweg, S. W., Lemmink, K. A. P. M., van Mechelen, W. & Diercks, R. L. Predictors of running-related injuries in novice runners enrolled in a systematic training program. *Am. J. Sports Med.* **38**, 273–280 (2010). 19966104 10.1177/0363546509347985

[31] Hespanhol Junior, L. C., Pena Costa, L. O. & Lopes, A. D. Previous injuries and some training characteristics predict running-related injuries in recreational runners: a prospective cohort study. *J. Physiother.* **59**, 263–269 (2013). 24287220 10.1016/S1836-9553(13)70203-0

[32] Rauh, M. J., Barrack, M. & Nichols, J. F. Associations between the female athlete triad and injury among high school runners. *Int J. Sports Phys. Ther.* **9**, 948–958 (2014). 25540710 PMC4275199

[33] Luedke, L. E., Heiderscheit, B. C., Blaise Williams, D. S. & Rauh, M. J. Association of isometric strength of hip and knee muscles with injury risk in high school cross country runners. *Int. J. Sports Phys. Ther.* **10**, 868–876 (2015). 26618066 PMC4637921

[34] Jungmalm, J. et al. Associations between biomechanical and clinical/anthropometrical factors and running-related injuries among recreational runners: a 52-week prospective cohort study. *Inj. Epidemiol.* **7**, 10 (2020). 32234070 10.1186/s40621-020-00237-2 PMC7110719

[35] Raissi, G. R. D., Cherati, A. D. S., Mansoori, K. D. & Razi, M. D. The relationship between lower extremity alignment and Medial Tibial Stress Syndrome among non-professional athletes. *BMC Sports Sci. Med. Rehabil.* **1**, 11 (2009). 10.1186/1758-2555-1-11 PMC2700791 19519909

[36] Rauh, M. J., Koepsell, T. D., Rivara, F. P., Rice, S. G. & Margherita, A. J. Quadriceps angle and risk of injury among high school cross-country runners. *J. Orthop. Sports Phys. Ther.* **37**, 725–733 (2007). 18560184 10.2519/jospt.2007.2453

[37] Davis, I. S., Bowser, B. J. & Mullineaux, D. R. Greater vertical impact loading in female runners with medically diagnosed injuries: a prospective investigation. *Br. J. Sports Med.* **50**, 887–892 (2016). 26644428 10.1136/bjsports-2015-094579

[38] Malisoux, L., Gette, P., Delattre, N., Urhausen, A. & Theisen, D. Spatiotemporal and ground-reaction force characteristics as risk factors for running-related injury: a secondary analysis of a randomized trial including 800+ recreational runners. *Am. J. Sports Med.* **50**, 537–544 (2022). 35049407 10.1177/03635465211063909

[39] Gerlach, K. E., Burton, H. W., Dorn, J. M., Leddy, J. J. & Horvath, P. J. Fat intake and injury in female runners. *J. Int. Soc. Sports Nutr.* **5**, 1 (2008). 10.1186/1550-2783-5-1 PMC2235827 18173851

[40] Walter, S. D., Hart, L. E., Mclntosh, J. M. & Sutton, J. R. The Ontario cohort study of running-related injuries. *Arch. Intern. Med.* **149**, 2561 (1989). 2818114

[41] Dijkhuis, T. B., Otter, R., Aiello, M., Velthuijsen, H. & Lemmink, K. Increase in the acute: chronic workload ratio relates to injury risk in competitive runners. *Int. J. Sports Med.*10.1055/a-1171-2331 (2020). 10.1055/a-1171-2331 32485779

[42] Leppänen, M. et al. Hip and core exercise programme prevents running-related overuse injuries in adult novice recreational runners: a three-arm randomised controlled trial (Run RCT). *Br. J. Sports Med.* **58**, 722–732 (2024). 38724071 10.1136/bjsports-2023-107926

[43] de Araujo Munhoz, F. B. et al. Posterior tibial tendinopathy associated with matrix metalloproteinase 13 promoter genotype and haplotype. *J. Gene Med.* **18**, 325–330 (2016). 27886420 10.1002/jgm.2934

[44] Raleigh, S. M. et al. Variants within the MMP3 gene are associated with Achilles tendinopathy: possible interaction with the COL5A1 gene. *Br. J. Sports Med.* **43**, 514–520 (2009). 19042922 10.1136/bjsm.2008.053892

[45] Nie, G. et al. Additional evidence supports association of common genetic variants in MMP3 and TIMP2 with increased risk of chronic Achilles tendinopathy susceptibility. *J. Sci. Med. Sport* **22**, 1074–1078 (2019). 31208828 10.1016/j.jsams.2019.05.021

[46] Nogara, P. R. B. et al. Association of estrogen receptor β polymorphisms with posterior tibial tendon dysfunction. *Mol. Cell Biochem.* **471**, 63–69 (2020). 32472323 10.1007/s11010-020-03765-z

[47] Leźnicka, K. et al. Interactions between Gene Variants within the COL1A1 and COL5A1 Genes and Musculoskeletal Injuries in Physically Active Caucasian. *Genes (Basel)* **12**, 1056 (2021). 34356072 10.3390/genes12071056 PMC8307722

[48] Pontin, P. A. et al. ERα PvuII and XbaI polymorphisms in postmenopausal women with posterior tibial tendon dysfunction: a case control study. *J. Orthop. Surg. Res.* **13**, 316 (2018). 30537990 10.1186/s13018-018-1020-x PMC6290490

[49] Bell, R. D., Shultz, S. J., Wideman, L. & Henrich, V. C. Collagen gene variants previously associated with anterior cruciate ligament injury risk are also associated with joint laxity. *Sports Health.* **4**, 312–318 (2012). 23016102 10.1177/1941738112446684 PMC3435918

[50] Hall, E. C. R. et al. The genetic association with injury risk in male academy soccer players depends on maturity status. *Scand. J. Med. Sci. Sports* **32**, 338–350 (2022). 34633711 10.1111/sms.14077

[51] Guo, R. et al. Association of COL5A1 gene polymorphisms and musculoskeletal soft tissue injuries: a meta-analysis based on 21 observational studies. *J. Orthop. Surg. Res.* **17**, 129 (2022). 35241120 10.1186/s13018-022-03020-9 PMC8895797

[52] Weeks, B. K. & Beck, B. R. The BPAQ: a bone-specific physical activity assessment instrument. *Osteoporos. Int.* **19**, 1567–1577 (2008). 18414964 10.1007/s00198-008-0606-2

[53] Luce, K. H. & Crowther, J. H. The reliability of the eating disorder examination-Self-report questionnaire version (EDE-Q). *Int. J. Eat. Disord.* **25**, 349–351 (1999). 10192002 10.1002/(sici)1098-108x(199904)25:3<349::aid-eat15>3.0.co;2-m

[54] Melin, A. et al. The LEAF questionnaire: a screening tool for the identification of female athletes at risk for the female athlete triad. *Br. J. Sports Med.* **48**, 540–545 (2014). 24563388 10.1136/bjsports-2013-093240

[55] Brody, D. M. Techniques in the evaluation and treatment of the injured runner. *Orthop. Clin. North Am.* **13**, 541–558 (1982). 6124922

[56] Clarsen, B. et al. Improved reporting of overuse injuries and health problems in sport: an update of the Oslo Sport Trauma Research Center questionnaires. *Br. J. Sports Med.* **54**, 390–396 (2020). 32060142 10.1136/bjsports-2019-101337

[57] Bredeweg, S. W., Kluitenberg, B., Bessem, B. & Buist, I. Differences in kinetic variables between injured and noninjured novice runners: a prospective cohort study. *J. Sci. Med. Sport* **16**, 205–210 (2013). 22921763 10.1016/j.jsams.2012.08.002

[58] Nguyen, N. H. et al. Machine learning-based prediction models for diagnosis and prognosis in inflammatory bowel diseases: a systematic review. *J. Crohns Colitis* **16**, 398–413 (2022). 34492100 10.1093/ecco-jcc/jjab155 PMC8919806

[59] Saarela, M. & Jauhiainen, S. Comparison of feature importance measures as explanations for classification models. *SN Appl. Sci.* **3**, 272 (2021).

[60] Urbanowicz, R. J., Meeker, M., La Cava, W., Olson, R. S. & Moore, J. H. Relief-based feature selection: introduction and review. *J. Biomed. Inf.* **85**, 189–203 (2018). 10.1016/j.jbi.2018.07.014 PMC6299836 30031057

[61] Bouchlaghem, Y., Akhiat, Y. & Amjad, S. Feature selection: a review and comparative study. *E3S Web. Conf.* **351**, 01046 (2022).

