# Supplementary material for: Multidisciplinary prediction of running-related injuries using machine learning
Source: NPJ Digit Med. 2026 Feb 6;9:213. doi: 10.1038/s41746-026-02413-y (PMC12987969; doi:10.1038/s41746-026-02413-y)
Supplement: Supplementary file 1 — Supplementary information [file 41746_2026_2413_MOESM1_ESM.pdf]

Table of Contents

Supplementary Material Section 1: Adherence, Descriptives, Injury Incidence, t-test results 2

Supplementary Material Section 2: Questionnaires 10

Supplementary Material Section 3: Experimental Protocols 22

Supplementary Material Section 4: Feature Description and Evidence Appraisal .. 24

Supplementary Material Section 5: Model Output 177

Click title to jump to corresponding section.

Supplementary Material Section 1: Adherence, Descriptives, Injury Incidence, t-test results

Flowchart shows participants adherence; supplementary tables 1 and 2 show participants' baseline descriptive statistics and injury incidence during the tracking period; supplementary tables 3-7 show two-sided independent t-test results for model AUC performance.

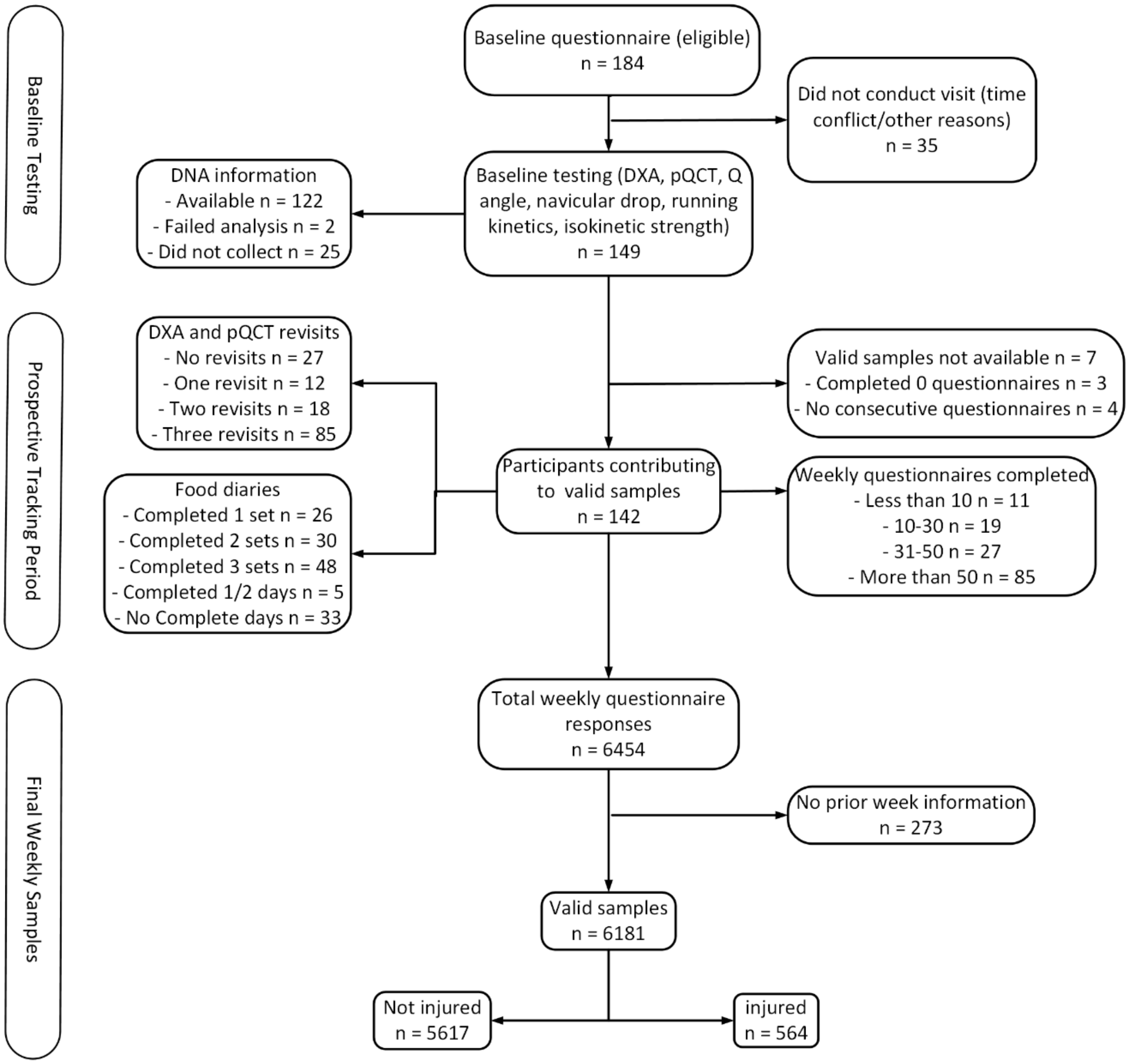

Supplementary Figure 1: Flowchart illustrating participant recruitment, drop-out, and adherence to data collection tasks during the study.

Supplementary Table 1: Descriptive statistics at baseline. BMI = body mass index. Data is presented as mean ± standard deviation.

	**Male (n=78)**	**Female (n=64)**	**Total (n=142)**
Age (years)	32.0 ± 9.7	30.9 ± 9.9	31.5 ± 9.8
Body mass (kg)	72.0 ± 8.6	55.8 ± 6.2	64.7 ± 11.1
Performance score[18]	584 ± 245	811 ± 181	686 ± 245
Past year running frequency (runs per week)	6.24 ± 2.00	5.42 ± 1.39	5.88 ± 1.80
BMI (kg.m^{-2})	22.3 ± 2.2	20.4 ± 1.9	21.4 ± 2.2
Past year injury days	42.4 ± 91.4	54.1 ± 91.7	47.7 ± 91.4
Participants injured past year (%)	55 (70.5)	53 (82.8)	108 (76.1)
EDE-Q score	0.46 ± 0.60	0.65 ± 0.85	0.55 ± 0.72
Hip abduction peak torque (N*m)	116.4 ± 28.0	87.9 ± 21.3	103.6 ± 28.9
Knee extension peak torque (N*m)	160.0 ± 38.5	109.8 ± 25.6	137.6 ± 41.6
Knee flexion peak torque (N*m)	89.7 ± 20.6	63.1 ± 13.2	77.8 ± 22.1
Navicular drop (cm)	0.50 ± 0.20	0.51 ± 0.25	0.50 ± 0.23
Quadriceps angle (°)	12.0 ± 3.8	15.0 ± 4.5	13.3 ± 4.4
Vertical impact peak (BW)	2.66 ± 0.23	2.66 ± 0.23	2.66 ± 0.23
Duty factor	0.63 ± 0.05	0.61 ± 0.05	0.62 ± 0.05

Supplementary Table 2. Injury incidence during the prospective tracking period by location.

	**Male (%; n=3544)**	**Female (%; n=2637)**	**Total (%; n=6181)**
Total injury	349	258	607[a]
Achilles tendon	47 (13.5)	29 (11.2)	76 (12.5)
Hip	46 (13.2)	27 (10.5)	73 (12.0)
Lateral knee	9 (2.6)	11 (4.3)	20 (3.3)
Middle knee	23 (6.6)	23 (8.9)	46 (7.6)
Medial knee	11 (3.2)	28 (10.9)	39 (6.4)
Back of knee	13 (3.7)	4 (1.6)	17 (2.8)
Ankle	29 (8.3)	21 (8.1)	50 (8.2)
Back of thigh	29 (8.3)	17 (6.6)	46 (7.6)
Lateral thigh	16 (4.6)	3 (1.2)	19 (3.1)
Shin	17 (4.9)	30 (11.6)	47 (7.7)
Sole of foot	26 (7.4)	14 (5.4)	40 (6.6)
Back of foot	8 (2.3)	2 (0.8)	10 (1.6)
Lower back	7 (2.0)	4 (1.6)	11 (1.8)
Other	68 (19.5)	45 (17.4)	113 (18.6)

[a]Total cases >564 (weeks of injury identification) because some weeks contain multiple injury occurrences.

Supplementary Table 3. Benjamini-Hochberg Q-value matrix of two-sided independent t-test for between-algorithm AUC differences when trained on class 1 feature set.

	Decision Tree	Random Forest	SVM	KNN	Naïve Bayes	Adaboost	Gradient Boost	MLP	Logistic Regression	Bayesian Network	TSNN	TSGNN
**Decision Tree**	*N/A*	**0.0192**	0.7962	0.1658	**0.0036**	**0.0548**	0.2518	0.4754	**0.0190**	**7.86e-04**	0.9163	0.5522
**Random Forest**	**0.0192**	*N/A*	**0.0036**	**0.0546**	**2.74e-05**	0.4279	**0.0368**	**0.0078**	**1.30e-04**	**1.32e-05**	**0.0188**	**0.0200**
**SVM**	0.7962	**0.0036**	*N/A*	**0.0871**	**6.03e-04**	**0.0195**	0.2003	0.4943	**0.0051**	**1.12e-04**	0.8738	0.6413
**KNN**	0.1658	**0.0546**	**0.0871**	*N/A*	**1.07e-04**	0.3044	0.6850	0.2518	**7.49e-04**	**3.51e-05**	0.1717	0.3044
**Naïve Bayes**	**0.0036**	**2.74e-05**	**6.03e-04**	**1.07e-04**	*N/A*	**3.80e-05**	**1.12e-04**	**2.32e-04**	0.6413	0.5121	**0.0024**	**3.61e-04**
**Adaboost**	**0.0548**	0.4279	**0.0195**	0.3044	**3.80e-05**	*N/A*	0.2089	**0.0546**	**2.90e-04**	**1.74e-05**	**0.0548**	**0.0885**
**Gradient Boost**	0.2518	**0.0368**	0.2003	0.6850	**1.12e-04**	0.2089	*N/A*	0.4943	**9.43e-04**	**3.51e-05**	0.2644	0.4943
**MLP**	0.4754	**0.0078**	0.4943	0.2518	**2.32e-04**	**0.0546**	0.4943	*N/A*	**0.0021**	**5.95e-05**	0.4952	0.8806
**Logistic Regression**	**0.0190**	**1.30e-04**	**0.0051**	**7.49e-04**	0.6413	**2.90e-04**	**9.43e-04**	**0.0021**	*N/A*	0.2970	**0.0137**	**0.0030**
**Bayesian Network**	**7.86e-04**	**1.32e-05**	**1.12e-04**	**3.51e-05**	0.5121	**1.74e-05**	**3.51e-05**	**5.95e-05**	0.2970	*N/A*	**4.96e-04**	**8.51e-05**
**TSNN**	0.9163	**0.0188**	0.8738	0.1717	**0.0024**	**0.0548**	0.2644	0.4952	**0.0137**	**4.96e-04**	*N/A*	0.6070
**TSGNN**	0.5522	**0.0200**	0.6413	0.3044	**3.61e-04**	**0.0885**	0.4943	0.8806	**0.0030**	**8.51e-05**	0.6070	*N/A*

Blue: q<0.05, green: q<0.001.

Supplementary Table 4. Benjamini-Hochberg Q-value matrix of one-sided independent t-test for between-algorithm AUC differences when trained on class 1 feature set.

	Decision Tree	Random Forest	SVM	KNN	Naïve Bayes	Adaboost	Gradient Boost	MLP	Logistic Regression	Bayesian Network	TSNN	TSGNN
**Decision Tree**	*N/A*				**0.0018**				**0.0095**	**3.93e-04**		
**Random Forest**	**0.0096**	*N/A*	**0.0018**	**0.0273**	**1.37e-05**	0.2140	**0.0184**	**0.0039**	**6.50e-05**	**6.62e-06**	**0.0094**	**0.0100**
**SVM**	0.3981		*N/A*		**3.01e-04**				**0.0026**	**5.62e-05**	0.4369	
**KNN**	**0.0829**		**0.0435**	*N/A*	**5.34e-05**		0.3425	0.1259	**3.75e-04**	**1.75e-05**	**0.0859**	0.1522
**Naïve Bayes**					*N/A*					0.2561		
**Adaboost**	**0.0274**		**0.0098**	0.1522	**1.90e-05**	*N/A*	0.1045	**0.0273**	**1.45e-04**	**8.70e-06**	**0.0274**	**0.0442**
**Gradient Boost**	0.1259		0.1001		**5.62e-05**		*N/A*	0.2472	**4.72e-04**	**1.75e-05**	0.1322	0.2472
**MLP**	0.2377		0.2472		**1.16e-04**			*N/A*	**0.0010**	**2.98e-05**	0.2476	0.4403
**Logistic Regression**					0.3207				*N/A*	0.1485		
**Bayesian Network**										*N/A*		
**TSNN**	0.4582				**0.0012**				**0.0068**	**2.48e-04**	*N/A*	
**TSGNN**	0.2761		0.3207		**1.81e-04**				**0.0015**	**4.25e-05**	0.3035	*N/A*

Comparisons made to test row>column; greyed out grids denote row<column. Blue: q<0.05, green: q<0.001.

Supplementary Table 5. Benjamini-Hochberg Q-value matrix of two-side independent t-test for between-algorithm AUC differences when trained on all features.

	Decision Tree	Random Forest	SVM	KNN	Naïve Bayes	Adaboost	Gradient Boost	MLP	Logistic Regression	Bayesian Network	TSNN	TSGNN
**Decision Tree**	*N/A*	**0.0140**	0.4221	0.2081	0.1381	**0.0203**	0.2112	0.6555	0.1552	**8.45e-04**	0.4129	0.3467
**Random Forest**	**0.0140**	*N/A*	**0.0055**	**0.0653**	**5.38e-04**	0.8358	**0.0742**	**0.0484**	0.1011	**6.79e-06**	**0.0562**	**0.0141**
**SVM**	0.4221	**0.0055**	*N/A*	0.5590	**0.0147**	**0.0203**	0.5590	0.8358	0.4129	**4.81e-05**	0.8358	0.8358
**KNN**	0.2081	**0.0653**	0.5590	*N/A*	**0.0078**	0.1384	1.0000	0.5590	0.8358	**2.38e-05**	0.7728	0.7368
**Naïve Bayes**	0.1381	**5.38e-04**	**0.0147**	**0.0078**	*N/A*	**6.80e-04**	**0.0078**	**0.0484**	**0.0057**	**0.0447**	**0.0175**	**0.0127**
**Adaboost**	**0.0203**	0.8358	**0.0203**	0.1384	**6.80e-04**	*N/A*	0.1486	**0.0742**	0.2049	**6.79e-06**	0.1011	**0.0452**
**Gradient Boost**	0.2112	**0.0742**	0.5590	1.0000	**0.0078**	0.1486	*N/A*	0.5590	0.8358	**2.38e-05**	0.7728	0.7369
**MLP**	0.6555	**0.0484**	0.8358	0.5590	**0.0484**	**0.0742**	0.5590	*N/A*	0.4583	**2.65e-04**	0.7728	0.7679
**Logistic Regression**	0.1552	0.1011	0.4129	0.8358	**0.0057**	0.2049	0.8358	0.4583	*N/A*	**2.38e-05**	0.6555	0.5590
**Bayesian Network**	**8.45e-04**	**6.79e-06**	**4.81e-05**	**2.38e-05**	**0.0447**	**6.79e-06**	**2.38e-05**	**2.65e-04**	**2.38e-05**	*N/A*	**4.81e-05**	**3.81e-05**
**TSNN**	0.4129	**0.0562**	0.8358	0.7728	**0.0175**	0.1011	0.7728	0.7728	0.6555	**4.81e-05**	*N/A*	1.0000
**TSGNN**	0.3467	**0.0141**	0.8358	0.7368	**0.0127**	**0.0452**	0.7369	0.7679	0.5590	**3.81e-05**	1.0000	*N/A*

Blue: q<0.05, green: q<0.001.

Supplementary Table 6. Benjamini-Hochberg Q-value matrix of one-sided independent t-test for between-algorithm AUC differences when trained on all features.

	Decision Tree	Random Forest	SVM	KNN	Naïve Bayes	Adaboost	Gradient Boost	MLP	Logistic Regression	Bayesian Network	TSNN	TSGNN
**Decision Tree**	*N/A*				**0.0690**					**4.22e-04**		
**Random Forest**	**0.0070**	*N/A*	**0.0028**	**0.0326**	**2.69e-04**	0.4179	**0.0371**	**0.0242**	**0.0505**	**3.39e-06**	**0.0281**	**0.0071**
**SVM**	0.2110		*N/A*		**0.0074**			0.4179		**2.41e-05**		
**KNN**	0.1040		0.2795	*N/A*	**0.0039**		0.5000	0.2795		**1.19e-05**	0.3864	0.3684
**Naïve Bayes**					*N/A*					**0.0224**		
**Adaboost**	**0.0102**		**0.0102**	**0.0692**	**3.40e-04**	*N/A*	**0.0743**	**0.0371**	0.1024	**3.39e-06**	**0.0505**	**0.0226**
**Gradient Boost**	0.1056		0.2795		**0.0039**		*N/A*	0.2795		**1.19e-05**	0.3864	0.3685
**MLP**	0.3277				**0.0242**			*N/A*		**1.32e-04**		
**Logistic Regression**	**0.0776**		0.2065	0.4179	**0.0029**		0.4179	0.2292	*N/A*	**1.19e-05**	0.3277	0.2795
**Bayesian Network**										*N/A*		
**TSNN**	0.2065		0.4179		**0.0088**			0.3864		**2.41e-05**	*N/A*	0.5000
**TSGNN**	0.1733		0.4179		**0.0063**			0.3840		**1.91e-05**		*N/A*

Comparisons made to test row>column; greyed out grids denote row<column. Blue: q<0.05, green: q<0.001.

Supplementary Table 7. Benjamini-Hochberg Q-values for two-sided independent t-test results for within-algorithm AUC comparison when trained using class 1 vs. all features.

Classifier	FS1_Mean	FS2_Mean	Difference_FS2_minus_FS1	Percent_Change	t_stat	p_value	q_value	FDR_sig
Decision Tree	0.734	0.734	0.000	0.0%	0.000	1.000000	1.000000	
Random Forest	0.781	0.784	+0.003	0.4%	-0.319	0.753307	1.000000	
SVM	0.739	0.750	+0.011	1.5%	-1.054	0.306871	0.864420	
KNN	0.760	0.759	-0.001	-0.1%	+0.101	0.921133	1.000000	
Naïve Bayes	0.663	0.698	**+0.035**	**5.3%**	-1.865	0.078621	0.471726	
Adaboost	0.771	0.781	+0.010	1.3%	-0.939	0.360175	0.864420	
Gradient Boost	0.756	0.759	+0.003	0.4%	-0.271	0.789367	1.000000	
MLP	0.748	0.746	-0.002	-0.3%	+0.139	0.891835	1.000000	
Logistic Regression	0.674	0.762	**+0.088**	**13.1%**	-5.111	0.000154	**0.001848**	**
Bayesian Network	0.649	0.649	0.000	0.0%	0.000	1.000000	1.000000	
TSNN	0.736	0.753	+0.017	2.3%	-1.022	0.320965	0.864420	
TSGNN	0.746	0.753	+0.007	0.9%	-0.582	0.568391	1.000000	

Supplementary Material Section 2: Questionnaires

The baseline questionnaire was completed before participants scheduled for their initial visits. The weekly questionnaire was for weekly injury and training tracking, and was completed every week during the tracking period.

Baseline Questionnaire

Basic Information

1. Please enter your first name.

2. Please enter your last name.

3. Please select your date of birth.

4. Please select your biological sex. This refers to your physiological sex at the time of birth.

4.a. If you selected Other, please specify:

5. Please select the date you complete this questionnaire (the date today)

Health Screen

6. At present, do you have any health problem for which you are:

6.1.a. (a) on medication, prescribed or otherwise

7. Have you ever had any of the following:

7.1.a. (a) Convulsions/epilepsy

7.2.a. (b) Asthma

7.3.a. (c) Eczema

7.4.a. (d) Diabetes

7.5.a. (e) A blood disorder

7.6.a. (f) Head injury

7.7.a. (g) Digestive problems

7.8.a. (h) Heart problems/chest pains

7.9.a. (i) Problems with muscles, bones or joints

7.10.a. (j) Disturbance of balance/coordination

7.11.a. (k) Numbness in hands or feet

7.12.a. (l) Disturbance of vision

7.13.a. (m) Ear/hearing problems

7.14.a. (n) Thyroid problems

7.15.a. (o) Kidney or liver problems

7.16.a. (p) Problems with blood pressure

7.a. If YES to any question, please describe briefly if you wish (eg to confirm problem was/is short-lived, insignificant or well controlled.)

8. Smoking, physical activity and family history

8.1.a. (a) Are you a current or recent (within the last six months) smoker or vaper?

9. Allergy Information

9.1.a. (a) Are you allergic to any food products?

9.2.a. (b) Are you allergic to any medicines?

9.3.a. (c) Are you allergic to plasters?

9.4.a. (d) Are you allergic to latex?

9.a. If YES to any of the above, please provide additional information on the allergy

10. Additional questions for female participants

10.1.a. (a) Are your periods normal/regular?

10.2.a. (b) Are you on hormonal contraception

10.3.a. (c) Are you taking hormone replacement therapy (HRT)?

11. Stress fracture history

11.1.a. (a) Have you ever been diagnosed with a bone stress injury (stress fracture, stress reaction, stress response)?

11.a. If yes, please provide details of type of injury, age of occurrence, anatomical location, time of year and method of diagnosis.

12. Research involvement

12.1.a. (a) Are you currently involved in any other research studies at the University or elsewhere?

12.a. If yes, please provide details.

13. Please provide the name of your emergency contact in the event of any incident or emergency.

13.a. Telephone number

13.b. Relationship to participant

Past 12-month Injuries

14. Please indicate the location of the problem(s) you have experienced over the last 12 months and the number of days you were forced to reduce your normal running routine because of each problem:

14.1.a. 1 - Location of the problem

14.1.b. 1 - Number of days the problem lasted

14.1.c. 1 - Did you visit a healthcare professional (e.g. physiotherapist), to obtain a diagnosis?

14.2.a. 2 - Location of the problem

14.2.b. 2 - Number of days the problem lasted

14.2.c. 2 - Did you visit a healthcare professional (e.g. physiotherapist), to obtain a diagnosis?

14.3.a. 3 - Location of the problem

14.3.b. 3 - Number of days the problem lasted

14.3.c. 3 - Did you visit a healthcare professional (e.g. physiotherapist), to obtain a diagnosis?

14.4.a. 4 - Location of the problem

14.4.b. 4 - Number of days the problem lasted

14.4.c. 4 - Did you visit a healthcare professional (e.g. physiotherapist), to obtain a diagnosis?

14.5.a. 5 - Location of the problem

14.5.b. 5 - Number of days the problem lasted

14.5.c. 5 - Did you visit a healthcare professional (e.g. physiotherapist), to obtain a diagnosis?

14.6.a. 6 - Location of the problem

14.6.b. 6 - Number of days the problem lasted

14.6.c. 6 - Did you visit a healthcare professional (e.g. physiotherapist), to obtain a diagnosis?

14.7.a. 7 - Location of the problem

14.7.b. 7 - Number of days the problem lasted

14.7.c. 7 - Did you visit a healthcare professional (e.g. physiotherapist), to obtain a diagnosis?

14.8.a. 8 - Location of the problem

14.8.b. 8 - Number of days the problem lasted

14.8.c. 8 - Did you visit a healthcare professional (e.g. physiotherapist), to obtain a diagnosis?

14.9.a. 9 - Location of the problem

14.9.b. 9 - Number of days the problem lasted

14.9.c. 9 - Did you visit a healthcare professional (e.g. physiotherapist), to obtain a diagnosis?

14.10.a. 10 - Location of the problem

14.10.b. 10 - Number of days the problem lasted

14.10.c. 10 - Did you visit a healthcare professional (e.g. physiotherapist), to obtain a diagnosis?

14.a. If there is any further information you would like to add, please describe below:

Bone-specific Physical Activity Questionnaire (BPAQ): standard questionnaire; see references within article

Athletic Performance Level

17. Please list the fastest times you have recorded in races or time trials over the following distances in the last 6 months:

17.1.a. 5000m track - Time

17.2.a. 5km road - Time

17.3.a. 10,000m track - Time

17.4.a. 10km road - Time

17.5.a. Half Marathon - Time

17.6.a. Marathon - Time

17.7.a. Other distance (5km or further): - Time

17.a. If there is any further information you would like to specify, please describe:

Past 12-month Training and S&C

18. Typically, how many hours did you run per week over the last 12 months during the following training phases?

18.1.a. Preparatory (off-season) period: - hours per week

18.2.a. Competitive (in-season or tapering) period: - hours per week

19. Typically, how often (runs per week) did you run per week over the last 12 months during the following training phases?

19.1.a. Preparatory (off-season) period: - times per week

19.2.a. Competitive (in-season or tapering) period: - times per week

20. Typically, how often have you performed intensive running sessions (speeds faster than half-marathon intensity, i.e. interval training and tempo running) per week over the last 12 months during the following training phases?

20.1.a. Preparatory (off-season) period: - times per week

20.2.a. Competitive (in-season or tapering) period: - times per week

21. In addition to your running sessions, did you include any other forms of aerobic exercise training over the last 12 months, and if so, typically how many hours and how often per week (sessions per week)?

21.1.a. Swimming - Yes/No

21.1.b. Swimming - If yes, hours per week

21.1.c. Swimming - If yes, how often per week

21.2.a. Cycling - Yes/No

21.2.b. Cycling - If yes, hours per week

21.2.c. Cycling - If yes, how often per week

21.3.a. Rowing - Yes/No

21.3.b. Rowing - If yes, hours per week

21.3.c. Rowing - If yes, how often per week

21.4.a. Cross-trainer - Yes/No

21.4.b. Cross-trainer - If yes, hours per week

21.4.c. Cross-trainer - If yes, how often per week

21.5.a. Other1 - Yes/No

21.5.b. Other1 - If yes, hours per week

21.5.c. Other1 - If yes, how often per week

21.6.a. Other2 - Yes/No

21.6.b. Other2 - If yes, hours per week

21.6.c. Other2 - If yes, how often per week

21.7.a. Other3 - Yes/No

21.7.b. Other3 - If yes, hours per week

21.7.c. Other3 - If yes, how often per week

21.a. Plese specify what "other" exercises refer to, if applicable:

21.a.1.a. other1 - Name of exercise

21.a.2.a. other2 - Name of exercise

21.a.3.a. other3 - Name of exercise

22. Over the last 12 months, have you participated in the following training activities, and if so, typically how many hours and how often per week (sessions per week)?

22.1.a. Resistance training (i.e. free weights, kettlebells, machines, elastic bands, medicine balls) - Yes/No

22.1.b. Resistance training (i.e. free weights, kettlebells, machines, elastic bands, medicine balls) - If yes, hours per week

22.1.c. Resistance training (i.e. free weights, kettlebells, machines, elastic bands, medicine balls) - If yes, how often per week

22.2.a. Plyometrics (i.e. jumping, hopping, bounding) - Yes/No

22.2.b. Plyometrics (i.e. jumping, hopping, bounding) - If yes, hours per week

22.2.c. Plyometrics (i.e. jumping, hopping, bounding) - If yes, how often per week

22.3.a. Core stability (i.e. exercises that specifically target the trunk/abdominal region) - Yes/No

22.3.b. Core stability (i.e. exercises that specifically target the trunk/abdominal region) - If yes, hours per week

22.3.c. Core stability (i.e. exercises that specifically target the trunk/abdominal region) - If yes, how often per week

22.4.a. Bodyweight exercises (i.e. burpees, press-ups, lunges without external load) - Yes/No

22.4.b. Bodyweight exercises (i.e. burpees, press-ups, lunges without external load) - If yes, hours per week

22.4.c. Bodyweight exercises (i.e. burpees, press-ups, lunges without external load) - If yes, how often per week

22.5.a. Stretching or yoga - Yes/No

22.5.b. Stretching or yoga - If yes, hours per week

22.5.c. Stretching or yoga - If yes, how often per week

23. Other than the activities mentioned above, were there any other sports or other physical activities (be as specific as possible) that you participated in regularly over the last 12 months, and if so, typically how many hours and how often per week (sessions per week)?

23.1.a. Activity1 - Name of activity

23.1.b. Activity1 - Hours per week

23.1.c. Activity1 - How often per week

23.2.a. Activity2 - Name of activity

23.2.b. Activity2 - Hours per week

23.2.c. Activity2 - How often per week

23.3.a. Activity3 - Name of activity

23.3.b. Activity3 - Hours per week

23.3.c. Activity3 - How often per week

23.4.a. Activity4 - Name of activity

23.4.b. Activity4 - Hours per week

23.4.c. Activity4 - How often per week

23.5.a. Activity5 - Name of activity

23.5.b. Activity5 - Hours per week

23.5.c. Activity5 - How often per week

23.6.a. Activity6 - Name of activity

23.6.b. Activity6 - Hours per week

23.6.c. Activity6 - How often per week

23.7.a. Activity7 - Name of activity

23.7.b. Activity7 - Hours per week

23.7.c. Activity7 - How often per week

24. If there is additional information you would like to specify, please describe:

Eating Disorder Examination Questionnaire (EDE-Q)): standard questionnaire; see references within article

Low Energy Availability in Females Questionnaire (LEAF-Q)): standard questionnaire; see references within article

Weekly Questionnaire

Q20_1. How many kilometers did you run at the following intensities during the past 7 days? - Low-moderate intensity (easy/recovery run; can hold conversation while running; 1-4 out of 10 perceived exertion)

Q20_2. How many kilometers did you run at the following intensities during the past 7 days? - Steady/heavy intensity (tempo run or around half-marathon pace; can say no more than 5 words while running; 5-6 out of 10 perceived exertion)

Q20_3. How many kilometers did you run at the following intensities during the past 7 days? - Severe/High intensity (intervals at 10km – 1500m/mile pace; very hard to talk while running; 7-10 out of 10 perceived exertion)

Q20_4. How many kilometers did you run at the following intensities during the past 7 days? - Very high intensity (sprint intervals at faster than 1500m/mile pace)

Q23_1. What is the typical speed you ran at the above-mentioned intensities (kilometers/hour)? - Low-moderate intensity (easy/recovery run; can hold conversation while running)

Q23_2. What is the typical speed you ran at the above-mentioned intensities (kilometers/hour)? - Steady/heavy intensity (tempo run or around half-marathon pace; can say no more than 5 words while running)

Q23_3. What is the typical speed you ran at the above-mentioned intensities (kilometers/hour)? - Severe/High intensity (intervals at 10km – 1500m/mile pace; very hard to talk while running)

Q23_4. What is the typical speed you ran at the above-mentioned intensities (kilometers/hour)? - Very high intensity (sprint intervals at faster than 1500m/mile pace)

Q30_1. How many minutes of non-running exercises did you perform during the past 7 days that could be represented by the descriptions below? - resistance training (e.g. free weights, kettlebells, machines, elastic bands, medicine balls)

Q30_2. How many minutes of non-running exercises did you perform during the past 7 days that could be represented by the descriptions below? - bodyweight exercises (e.g. unweighted lunges, wall squats)

Q30_16. How many minutes of non-running exercises did you perform during the past 7 days that could be represented by the descriptions below? - core stability exercises (e.g. planks, side planks, swiss ball-based, exercises specifically targeting trunk/abdominal region)

Q30_19. How many minutes of non-running exercises did you perform during the past 7 days that could be represented by the descriptions below? - balance training (e.g. exercises on an unstable surface)

Q30_3. How many minutes of non-running exercises did you perform during the past 7 days that could be represented by the descriptions below? - plyometric exercises (e.g. jumping, hopping, bounding)

Q30_18. How many minutes of non-running exercises did you perform during the past 7 days that could be represented by the descriptions below? - running technique drills (e.g. A- and B- drills, dribbles)

Q30_5. How many minutes of non-running exercises did you perform during the past 7 days that could be represented by the descriptions below? - circuit training

Q30_20. How many minutes of non-running exercises did you perform during the past 7 days that could be represented by the descriptions below? - barefoot exercises

Q30_6. How many minutes of non-running exercises did you perform during the past 7 days that could be represented by the descriptions below? - stretching or yoga

Q30_12. How many minutes of non-running exercises did you perform during the past 7 days that could be represented by the descriptions below? - swimming

Q30_13. How many minutes of non-running exercises did you perform during the past 7 days that could be represented by the descriptions below? - cycling

Q30_14. How many minutes of non-running exercises did you perform during the past 7 days that could be represented by the descriptions below? - rowing

Q30_17. How many minutes of non-running exercises did you perform during the past 7 days that could be represented by the descriptions below? - cross-trainer/elliptical machine

Q30_7. How many minutes of non-running exercises did you perform during the past 7 days that could be represented by the descriptions below? - Other (please specify)

Q30_7_TEXT. How many minutes of non-running exercises did you perform during the past 7 days that could be represented by the descriptions below? - Other (please specify) - Text

Q32. If there are any other details regarding the exercises you completed during the past 7 days you would like to specify, please describe below:

Acknowledgement. Please answer all following questions regardless of whether or not you have physical problems. Select the alternative that is most appropriate for you, and in the case that you are unsure, try to answer as best you can anyway.

The term "physical problems" refers to pain, ache, stiffness, clicking/catching, swelling, instability/giving way, locking, or other complaints related to your joint, bone, tendon, ligament, or muscle.

1_Q3. Have you had any difficulties participating in training and competition due to (other) physical
problems during the past 7 days? ("The past 7 days" refers to the 7 consecutive days on and before the day this questionnaire is released, which is Sunday on each week)

1_Q4. Please select the location that best represents your physical problem. Select one, and you will come back to this question if you have more than one physical problems. - Selected Choice

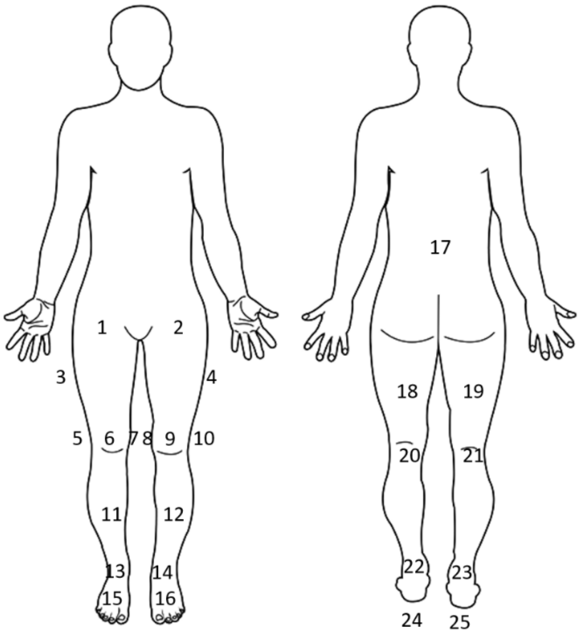

1_Q4_26_TEXT. Please select the location that best represents your physical problem. Select one, and you will come back to this question if you have more than one physical problems. - Other (please specify; you can use descriptions such as "between area X and area X", "at the X part of my body", or use anatomical descriptions if you are familiar with them) – Text

1_Q6. Have you had any difficulties participating in training and competition due to your selected problem during the past 7 days?

1_Q7. To what extent have you modified your training or competition due to your selected problem during the past 7 days?

1_Q8. To what extent has your selected problem affected your performance during the past 7
days?

1_Q9. To what extent have you experienced pain at your selected location related to your sport during the
past 7 days?

1_Q10. How would you describe the cause of your selected physical problem?

1_Q11. Would you describe your selected physical problem as caused by or related to running?

1_Q33. Have you obtained a diagnosis by a qualified healthcare professional (i.e. physician or physiotherapist) for your selected physical problem?

1_Q34. Please specify your diagnosis below.

1_Q12. If there are any other details regarding your selected physical problem you would like to specify, please describe below:

2_Q3. Have you had any difficulties participating in training and competition due to (other) physical
problems during the past 7 days? ("The past 7 days" refers to the 7 consecutive days on and before the day this questionnaire is released, which is Sunday on each week)

Note: all the injury-related questions will be looped if answered ‘yes’ when this question appears. Otherwise, the questionnaire is completed.

Supplementary Material Section 3: Experimental Protocols

This document contains the data collection procedures of non-self-reported (i.e., not collected through questionnaire) variables.

Bone Scans

DXA and pQCT measurement were made according to standard manufacturer guidelines.

Posture Measurements

Q Angle Measurement

Q angle was measured in a supine lying position. The thighs were placed vertically to the line connecting the anterior-superior iliac spines (ASIS), with knees facing anteriorly. Three dots were penned on the superior right, left, and inferior tips of the patella, before another dot was penned in the middle of the three. An additional dot was penned in the middle of the tibial tuberosity. A goniometer was placed with the center aligning with the middle patellar dot, one arm overlapping with the tibial tuberosity dot, and the other arming pointing towards the unilateral ASIS. A thread was stretched between the center of the goniometer and the unilateral ASIS to help align the goniometer arm; the participant was asked to hold the thread down.

Navicular Drop Measurement

Navicular heights were measured in both seated and standing positions to calculate navicular drop. The participant first adopted a comfortable standing position, then a dot is penned on each side’s protrusion of the navicular bone. A marking paper was used to mark the height of each dot. The participant was then comfortably seated with knees and feet pointing forward and tibia vertical to the ground, and the height of the penned dots were marked again using the same paper.

Treadmill GRF Test

Running kinetics were measured using an instrumented treadmill (Treadmetrix, Utah, US) at 10km/h and 12km/h running pace for 1 minute each. Sampling frequency was set at 1000Hz. Participants were asked to run for 3 minutes at each pace, and data collection

was conducted during the last minute. Data along the vertical (z) axis collected by the four sensors on the corners of the forceplate was taken forward for vGRF analysis.

Isokinetic Dynamometer SOP

An isokinetic dynamometer (Isomed 2000, D&R Ferstl GmbH, Hemau, Germany) was used to measure both sides' hip abduction/adduction and knee flexion/extension strengths at 60 degrees/s and 200 Hz sampling frequency. The participant was instructed to do forceful concentric contractions alternating between each couple of motions (abduction-adduction, flexion-extension). For each exercise, two practice sets and five measured sets, each consisting of four repetitions plus 1 minute rest period, were performed to avoid fatigue while providing sufficient exercise familiarisation. Participants were asked to use ~80% strength during the practice sets. Among the 20 measured repetitions, the top 3-7 were taken to remove potential high outliers due to compensation and low performance repetitions due to familiarisation. Dynamometer swivel arm lengths were adjusted based on measured participant limb lengths. All strength measurements were normalised by participants' body mass.

For hip abduction/adduction, participants were laid on their lateral side, with the measured leg attached to the swivel arm, and unmeasured thigh flexed at 45 degrees (allowing room for the measured leg's adduction). Participants were asked to maintain back and hip extension so that their back and measured leg remain at 180 degrees. They were allowed to hold on to the bench's side to exert strength but were not allowed to exert their hand and elbow lateral to their head to avoid forced spine lateral flexion.

During knee flexion/extension, participants were seated with their torso and tested leg strapped to the testing bench with lower legs hanging over the bench's side. Their hands were grabbed onto holders on both sides to help exert strength. They were asked to keep ankles dorsiflexed during knee flexion to allow gastrocnemius exertion and were reminded to keep their hip from slipping forward to prevent compensation using torso strength.

Food Diary Instructions

Participants were instructed to collect from two weekdays and one weekend day, and from days that represent their normal dietary habits. They were asked to weigh and record all food and drink intakes except for water. For cooked dishes, the closest match from Libro was selected.

DNA Extraction SOP

See Manufacturer Isolation Protocol for GFX-02 4ml GeneFix™ saliva sample (2ml saliva collected into 2ml lysis buffer)

Supplementary Material Section 4: Feature Description and Evidence Appraisal

Jump to: Baseline Questionnaire Isokinetic Strength Biomechanics and static posture Nutrition Bone Scans and Anthropometry Training (weekly questionnaire) Genetics

[*]: linked to descriptions at the end. Click to follow.

Evidence Appraisal Criteria

Criteria were separated for genetic features vs. non-genetic features. Since we were only looking at genotypes without obtaining any information on gene expression, all the genetic evidence we gathered was essentially prospective evidence since genetic makeup is determined at birth. As a result, a separate set of criteria that do not distinguish between prospective and cross-sectional evidence was established for genetic features.

Genetic Features

- Class 1: Gene associated with the target SNP has theoretical connections with soft tissue injuries/musculoskeletal injuries (such as type I collagen), plus research has shown a direct link between the target SNP and common running-related injuries (not necessarily within a runner population, but the type of injury is also common in runners).
- Class 1: Fulfill 1 of the 2 conditions above (theoretical connection of associated gene; research showing direct link with RRI).
- Class 3: Fulfill none of the 2 conditions or has >90% overlap in genotypic distribution with another SNP within the study population. When an overlapping case was discovered, a subjective judgement was made to downgrade the one with relatively lower quality of evidence.

Non-genetic Features

- Class 1: There is prospective evidence showing a direct link between the feature and running-related injuries. The study must be conducted directly on runners.
- Class 2: There is cross-sectional/retrospective evidence showing a direct link between the feature and running-related injuries. Studies conducted with non-runner populations were included as long as the injury type was common among runners.

- Class 3: There is only circumferential evidence supporting the feature (such as opinion article), or the feature makes theoretical sense but does not have research evidence to support. If a feature is considered to be highly correlated with other features, subjective judgements were made to keep 1 of all correlated features and leave all the rest to class 3.

Below are some examples of class assignments of non-genetic features:

1. A feature with high quality prospective evidence on runners, but some results showing no correlation. Pooled results showed no correlation. For instance, pooled meta-analysis showed no difference in injury risk between sexes, but there were differences on specific injury types between sexes. Class 1
2. A feature that has no prospective evidence on runners, but has prospective evidence on other sports. Class 2
3. A feature that has no prospective evidence on runners, but has retrospective/cross-sectional evidence. Class 2
4. A feature that has no evidence on runners, but has evidence linking it to common running-related injury types (such as PFPS, plantar fasciitis). Class 2 or 3 depending on quality of evidence
5. A feature that has no original study to support directly, but could make sense via inference from original studies on similar topics (such as the same asymmetry study cited for all the asymmetry features). Class 3

6. A feature that has no original study to support, but appeared in an opinion article potentially due to some circumstantial evidence (such as most of the nutrient features). Class 3
7. A feature that has no study to support, but the reason for that was likely because the measurement method is complex, and researchers often use simpler replacement methods (such as thigh and calf FFMI vs. BMI). Class 3

Supplementary Table 8: Baseline Questionnaire variables

Feature Name	Explanation	References	Evidence Appraisal
Sex		Jacobsson, J., Timpka, T., Kowalski, J., Nilsson, S., Ekberg, J., Dahlström, Ö., & Renström, P. A. (2013). Injury patterns in Swedish elite athletics: annual incidence, injury types and risk factors. *British Journal of Sports Medicine*, *47*(15), 941-952.	Multiple prospective studies with conflicting results. Different types of injuries also seem to

		Rauh, M. J., Koepsell, T. D., Rivara, F. P., Margherita, A. J., & Rice, S. G. (2006). Epidemiology of musculoskeletal injuries among high school cross-country runners. *American journal of epidemiology*, *163*(2), 151-159.<br>Messier, S. P., Martin, D. F., Mihalko, S. L., Ip, E., DeVita, P., Cannon, D. W., ... & Seay, J. F. (2018). A 2-year prospective cohort study of overuse running injuries: the runners and injury longitudinal study (TRAILS). *The American journal of sports medicine*, *46*(9), 2211-2221.<br>Hollander, K., Rahlf, A. L., Wilke, J., Edler, C., Steib, S., Junge, A., & Zech, A. (2021). Sex-specific differences in running injuries: a systematic review with meta-	have different variations between sexes. The last article is a systematic review and meta-analysis that contains a comprehensive summarisation of existing evidence.

		analysis and meta-regression. *Sports Medicine*, *51*, 1011-1039.	
Age	Age as of the date of completing the baseline questionnaire	Taunton, J. E., Ryan, M. B., Clement, D. B., McKenzie, D. C., Lloyd-Smith, D. R., & Zumbo, B. D. (2003). A prospective study of running injuries: the Vancouver Sun Run "In Training" clinics. *British journal of sports medicine*, *37*(3), 239-244.<br>Nielsen, R. O., Buist, I., Parner, E. T., Nohr, E. A., Sørensen, H., Lind, M., & Rasmussen, S. (2013). Predictors of running-related injuries among 930 novice runners: a 1-year prospective follow-up study. *Orthopaedic journal of sports medicine*, *1*(1), 2325967113487316.	Prospective investigations showed some link (one only found in women, the other not statistically significant)

		Taunton, J. E., Ryan, M. B., Clement, D. B., McKenzie, D. C., Lloyd-Smith, D. R., & Zumbo, B. D. (2002). A retrospective case-control analysis of 2002 running injuries. *British journal of sports medicine*, *36*(2), 95-101.	
Past_stress_injury	Y/N on being diagnosed with stress injury in the past	Kelsey, J. L., Bachrach, L. K., Procter-Gray, E., Nieves, J. E. R. I., Greendale, G. A., Sowers, M., ... & Cobb, K. L. (2007). Risk factors for stress fracture among young female cross-country runners. *Medicine & Science in Sports & Exercise*, *39*(9), 1457-1463.<br>Tenforde, A. S., Sayres, L. C., McCurdy, M. L., Sainani, K. L., & Fredericson, M. I. C. H. A. E. L. (2013). Identifying sex-specific risk factors for stress fractures in	Prospective evidence showing link between previous stress frature and future stress fracture. However, as stress fracture only forms a fraction of all

		adolescent runners. *Medicine & Science in Sports & Exercise*, *45*(10), 1843-1851.<br>Wright, A. A., Taylor, J. B., Ford, K. R., Siska, L., & Smoliga, J. M. (2015). Risk factors associated with lower extremity stress fractures in runners: a systematic review with meta-analysis. *British Journal of Sports Medicine*, *49*(23), 1517-1523.	injuries, this feature is downgraded.
lower_limb_days_total[1]	Total number of days affected by lower limb injuries during the past year	Buist, I., Bredeweg, S. W., Lemmink, K. A., Van Mechelen, W., & Diercks, R. L. (2010). Predictors of running-related injuries in novice runners enrolled in a systematic training program: a prospective cohort study. *The American journal of sports medicine*, *38*(2), 273-280.	Ample evidence in prospective studies showing links between previous RRI and future RRI.

		Theisen, D., Malisoux, L., Genin, J., Delattre, N., Seil, R., & Urhausen, A. (2014). Influence of midsole hardness of standard cushioned shoes on running-related injury risk. *British Journal of Sports Medicine*, *48*(5), 371-376.<br>Saragiotto, B. T., Yamato, T. P., Hespanhol Junior, L. C., Rainbow, M. J., Davis, I. S., & Lopes, A. D. (2014). What are the main risk factors for running-related injuries?. *Sports medicine*, *44*, 1153-1163.<br>Hulme, A., Nielsen, R. O., Timpka, T., Verhagen, E., & Finch, C. (2017). Risk and protective factors for middle- and long-distance running-related injury. *Sports Medicine*, *47*, 869-886.	

Athlete_Score[2]	Athlete score based on best performance reported (or gathered online) during the past year	Van Mechelen, W. (1992). Running injuries: a review of the epidemiological literature. *Sports medicine*, *14*, 320-335.<br>Fredette, A., Roy, J. S., Perreault, K., Dupuis, F., Napier, C., & Esculier, J. F. (2022). The association between running injuries and training parameters: a systematic review. *Journal of Athletic Training*, *57*(7), 650-671.	Comparison between studies show large differences among cohorts with different performance levels. However, it is suspected that this features correlates with training volume, thus it is downgraded.

average_run_hours	Average hours of running per week during the past year	Junior, L. C. H., Costa, L. O. P., & Lopes, A. D. (2013). Previous injuries and some training characteristics predict running-related injuries in recreational runners: a prospective cohort study. *Journal of Physiotherapy*, *59*(4), 263-269.<br>Kluitenberg, B., van der Worp, H., Huisstede, B. M., Hartgens, F., Diercks, R., Verhagen, E., & van Middelkoop, M. (2016). The NLstart2run study: Training-related factors associated with running-related injuries in novice runners. *Journal of Science and Medicine in Sport*, *19*(8), 642-646.<br>Fredette, A., Roy, J. S., Perreault, K., Dupuis, F., Napier, C., & Esculier, J. F. (2022). The association between	Prospective tracking studies show conflicting evidence. Most show no significant correlation between duration and injuries, some show significant correlations.

		running injuries and training parameters: a systematic review. *Journal of Athletic Training*, *57*(7), 650-671.	
average_run_frequency	Average number of running sessions per week during the past year	Taunton, J. E., Ryan, M. B., Clement, D. B., McKenzie, D. C., Lloyd-Smith, D. R., & Zumbo, B. D. (2003). A prospective study of running injuries: the Vancouver Sun Run "In Training" clinics. *British journal of sports medicine*, *37*(3), 239-244.<br>Malisoux, L., Ramesh, J., Mann, R., Seil, R., Urhausen, A., & Theisen, D. (2015). Can parallel use of different running shoes decrease running-related injury risk?. *Scandinavian journal of medicine & science in sports*, *25*(1), 110-115.<br>Fredette, A., Roy, J. S., Perreault, K., Dupuis, F., Napier, C., & Esculier, J. F. (2022). The association between	Prospective evidence show conflicting results, however this feature is correlated with average_run_hours.

		running injuries and training parameters: a systematic review. *Journal of Athletic Training*, *57*(7), 650-671.	
average_interval_training_frequency	Average number of interval training sessions per week during the past year	Junior, L. C. H., Costa, L. O. P., & Lopes, A. D. (2013). Previous injuries and some training characteristics predict running-related injuries in recreational runners: a prospective cohort study. *Journal of Physiotherapy*, *59*(4), 263-269.	Prospective evidence shows correlation.
EDEQ_total	EDEQ score	Rauh, M. J., Barrack, M., & Nichols, J. F. (2014). Associations between the female athlete triad and injury among high school runners. *International journal of sports physical therapy*, *9*(7), 948.<br>Rauh, M. J., Nichols, J. F., & Barrack, M. T. (2010). Relationships among injury and disordered eating, menstrual dysfunction, and low bone mineral density in	Prospective studies showed association between EDEQ overall and some subscale scores and RRIs.

		high school athletes: a prospective study. *Journal of athletic training*, *45*(3), 243-252.<br><br>Hamstra-Wright, K. L., Bliven, K. C. H., Coumbe-Lilley, J. E., Djelovic, E., & Patel, J. (2023). The relationship between eating disorders, disordered eating, and injury in athletes: a critically appraised topic. *Journal of Sport Rehabilitation*, *32*(4), 474-481.	
LEAF-Q[3]	LEAF-Q score	Barrack, M. T., Gibbs, J. C., De Souza, M. J., Williams, N. I., Nichols, J. F., Rauh, M. J., & Nattiv, A. (2014). Higher incidence of bone stress injuries with increasing female athlete triad–related risk factors: a prospective multisite study of exercising girls and women. *The American journal of sports medicine*, *42*(4), 949-958.	Prospective studies showed association between LEAF-Q and stress fratures in females. However, stress fratures only make up a fraction of

		Holtzman, B., Popp, K. L., Tenforde, A. S., Parziale, A. L., Taylor, K., & Ackerman, K. E. (2022). Low energy availability surrogates associated with lower bone mineral density and bone stress injury site. *PM&R*, *14*(5), 587-596.<br>Hamstra-Wright, K. L., Bliven, K. C. H., Coumbe-Lilley, J. E., Djelovic, E., & Patel, J. (2023). The relationship between eating disorders, disordered eating, and injury in athletes: a critically appraised topic. *Journal of Sport Rehabilitation*, *32*(4), 474-481.	all injuires, and females make up less than 1/2 of the cohort.

Supplementary Table 9: Isokinetic Strength variables

Feature Name	Explanation	References	Evidence Appraisal

Hip_abduction_peak_torque	The average peak torque of hip abduction for the top 3-7 reps (total of 4*5 reps, taking away 13 familiarisation and 2 potential compensation)	Becker, J. A. M. E. S., Nakajima, M. I. M. I., & Wu, W. F. (2018). Factors Contributing to Medial Tibial Stress Syndrome in Runners: A Prospective Study. *Medicine and science in sports and exercise*, *50*(10), 2092-2100.<br>Luedke, L. E., Heiderscheit, B. C., Williams, D. B., & Rauh, M. J. (2015). Association of isometric strength of hip and knee muscles with injury risk in high school cross country runners. *International journal of sports physical therapy*, *10*(6), 868.<br>Finnoff, J. T., Hall, M. M., Kyle, K., Krause, D. A., Lai, J., & Smith, J. (2011). Hip strength and knee pain in high school runners: a prospective study. *PM&R*, *3*(9), 792-801.	Prospective studies show correlations between hip abductor strength and RRIs.

		Mucha, M. D., Caldwell, W., Schlueter, E. L., Walters, C., & Hassen, A. (2017). Hip abductor strength and lower extremity running related injury in distance runners: a systematic review. *Journal of science and medicine in sport*, *20*(4), 349-355.<br>Christopher, S. M., McCullough, J., Snodgrass, S. J., & Cook, C. (2019). Do alterations in muscle strength, flexibility, range of motion, and alignment predict lower extremity injury in runners: a systematic review. *Archives of Physiotherapy*, *9*, 1-14.<br>de Marche Baldon, R., Nakagawa, T. H., Muniz, T. B., Amorim, C. F., Maciel, C. D., & Serrão, F. V. (2009). Eccentric hip muscle function in females with and	

		without patellofemoral pain syndrome. *Journal of athletic training*, *44*(5), 490-496.<br>Neal, B. S., Lack, S. D., Lankhorst, N. E., Raye, A., Morrissey, D., & Van Middelkoop, M. (2019). Risk factors for patellofemoral pain: a systematic review and meta-analysis. *British Journal of Sports Medicine*, *53*(5), 270-281.	
Hip_abduction_peak_angle	The average joint angle at peak torque of the 5 reps used above, measured as the angle of the isokinetic rotor arm	Brughelli, M., Cronin, J., & Nosaka, K. (2010). Muscle architecture and optimum angle of the knee flexors and extensors: a comparison between cyclists and Australian Rules football players. *The Journal of Strength & Conditioning Research*, *24*(3), 717-721.	Mostly speculative.
hip_abduction_peak_torq	The absolute value of the difference between the left and right hip	De Blaiser, C., Roosen, P., Willems, T., De Bleecker, C., Vermeulen, S., Danneels, L., & De Ridder, R. (2021). The	Prospective studies show correlations in

ue_asymmetry	abduction peak torque divided by their total value	role of core stability in the development of non-contact acute lower extremity injuries in an athletic population: A prospective study. *Physical Therapy in Sport*, *47*, 165-172.<br>Hietamo, J., Pasanen, K., Leppänen, M., Steffen, K., Kannus, P., Heinonen, A., ... & Parkkari, J. (2021). Association between lower extremity muscle strength and acute ankle injury in youth team-sports athletes. *Physical Therapy in Sport*, *48*, 188-195.<br>Niemuth, P. E., Johnson, R. J., Myers, M. J., & Thieman, T. J. (2005). Hip muscle weakness and overuse injuries in recreational runners. *Clinical Journal of Sport Medicine*, *15*(1), 14-21.	sporting populations but not runners specifically.

		Guan, Y., Bredin, S. S., Taunton, J., Jiang, Q., Wu, N., & Warburton, D. E. (2022). Association between inter-limb asymmetries in lower-limb functional performance and sport injury: a systematic review of prospective cohort studies. *Journal of clinical medicine*, *11*(2), 360.	
hip_abduction_peak_angle_asymmetry	The difference between the left and right hip abduction peak angle, NOT normalised by the mean		Speculative.
Hip_adduction_peak_torque		de Marche Baldon, R., Nakagawa, T. H., Muniz, T. B., Amorim, C. F., Maciel, C. D., & Serrão, F. V. (2009). Eccentric hip muscle function in females with and without patellofemoral pain syndrome. *Journal of athletic training*, *44*(5), 490-496.	Cross-sectional evidence links hip adduction strength to PFPS which is a common RRI.

Hip_adduction_peak_angle			Speculative
hip_adduction_peak_torque_asymmetry		Niemuth, P. E., Johnson, R. J., Myers, M. J., & Thieman, T. J. (2005). Hip muscle weakness and overuse injuries in recreational runners. *Clinical Journal of Sport Medicine*, *15*(1), 14-21.<br>Guan, Y., Bredin, S. S., Taunton, J., Jiang, Q., Wu, N., & Warburton, D. E. (2022). Association between inter-limb asymmetries in lower-limb functional performance and sport injury: a systematic review of prospective cohort studies. *Journal of clinical medicine*, *11*(2), 360.	Cross-sectional study shows injured runners had greater asymmetry in isometric hip adduction strength.

hip_adduction_peak_angle_asymmetry			Speculative
Total_ad_ab_ratio	The ratio between the left and right hip adduction and abduction average peak torque	Jungmalm, J., Nielsen, R. Ø., Desai, P., Karlsson, J., Hein, T., & Grau, S. (2020). Associations between biomechanical and clinical/anthropometrical factors and running-related injuries among recreational runners: a 52-week prospective cohort study. *Injury epidemiology*, *7*, 1-9.<br>Finnoff, J. T., Hall, M. M., Kyle, K., Krause, D. A., Lai, J., & Smith, J. (2011). Hip strength and knee pain in high school runners: a prospective study. *PM&R*, *3*(9), 792-801.	Prospective studies shows link with RRIs.

		Ferber, R., Hreljac, A., & Kendall, K. D. (2009). Suspected mechanisms in the cause of overuse running injuries: a clinical review. *Sports health*, *1*(3), 242-246.	
Ad_ab_ratio_asymmetry	The difference between the left and right adduction and abduction average peak torque ratio		Speculative.
Knee_extension_peak_torque		Luedke, L. E., Heiderscheit, B. C., Williams, D. B., & Rauh, M. J. (2015). Association of isometric strength of hip and knee muscles with injury risk in high school cross country runners. *International journal of sports physical therapy*, *10*(6), 868. Peterson, B., Hawke, F., Spink, M., Sadler, S., Hawes, M., Callister, R., & Chuter, V. (2022). Biomechanical and	Prospective study shows correlation, pooled analysis from a meta-analysis also shows significant correlation despite

		musculoskeletal measurements as risk factors for running-related injury in non-elite runners: A systematic review and meta-analysis of prospective studies. *Sports medicine-open*, *8*(1), 38.<br>McGuire, B., & King, B. (2021). Neuromuscular risk factors for non-contact knee injury: a systematic review and meta-analysis. *medRxiv*, 2021-09.<br>Neal, B. S., Lack, S. D., Lankhorst, N. E., Raye, A., Morrissey, D., & Van Middelkoop, M. (2019). Risk factors for patellofemoral pain: a systematic review and meta-analysis. *British Journal of Sports Medicine*, *53*(5), 270-281.	each included study not significant.

Knee_extension_peak_angle			Speculative.
Knee_extension_peak_torque_asymmetry		Fousekis, K., Tsepis, E., Poulmedis, P., Athanasopoulos, S., & Vagenas, G. (2011). Intrinsic risk factors of non-contact quadriceps and hamstring strains in soccer: a prospective study of 100 professional players. *British journal of sports medicine*, *45*(9), 709-714. Guan, Y., Bredin, S. S., Taunton, J., Jiang, Q., Wu, N., & Warburton, D. E. (2022). Association between inter-limb asymmetries in lower-limb functional performance and sport injury: a systematic review of prospective cohort studies. *Journal of clinical medicine*, *11*(2), 360.	Prospective study shows association within soccer players.

Knee_extension_peak_angle_asymmetry			Speculative.
Knee_flexion_peak_torque		Luedke, L. E., Heiderscheit, B. C., Williams, D. B., & Rauh, M. J. (2015). Association of isometric strength of hip and knee muscles with injury risk in high school cross country runners. *International journal of sports physical therapy*, *10*(6), 868.<br>Hein, T., Janssen, P., Wagner-Fritz, U., Haupt, G., & Grau, S. (2014). Prospective analysis of intrinsic and extrinsic risk factors on the development of achilles tendon pain in runners. *Scandinavian Journal of Medicine & Science in Sports*, *24*(3), e201-e212.	Prospective studies show association with RRIs and achilles tendon pain.

Knee_flexion _peak_angle		Timmins, R. G., Shield, A. J., Williams, M. D., & Opar, D. A. (2016). Is there evidence to support the use of the angle of peak torque as a marker of hamstring injury and re-injury risk?. *Sports Medicine*, *46*, 7-13.	Some theoretical basis with sprinters but not robust.
Knee_flexion _peak_torque _asymmetry		Knapik, J. J., Bauman, C. L., Jones, B. H., Harris, J. M., & Vaughan, L. (1991). Preseason strength and flexibility imbalances associated with athletic injuries in female collegiate athletes. *The American journal of sports medicine*, *19*(1), 76-81.<br>Fousekis, K., Tsepis, E., Poulmedis, P., Athanasopoulos, S., & Vagenas, G. (2011). Intrinsic risk factors of non-contact quadriceps and hamstring strains in soccer: a prospective study of 100 professional players. *British journal of sports medicine*, *45*(9), 709-714.	Prospective evidence present in general sporting population and soccer players.

		Grazioli, R., Sobieski, N., Wilhelm, E. N., Brusco, C. M., & Rech, A. (2022). Divergent isokinetic muscle strength deficits in street running athletes. *Sport Sciences for Health*, 1-8.<br>Guan, Y., Bredin, S. S., Taunton, J., Jiang, Q., Wu, N., & Warburton, D. E. (2022). Association between inter-limb asymmetries in lower-limb functional performance and sport injury: a systematic review of prospective cohort studies. *Journal of clinical medicine*, *11*(2), 360.	
Knee_flexion_peak_angle_asymmetry			Speculative
Total_fl_ex_ratio		Knapik, J. J., Bauman, C. L., Jones, B. H., Harris, J. M., & Vaughan, L. (1991). Preseason strength and flexibility	Prospective evidence present in

		imbalances associated with athletic injuries in female collegiate athletes. *The American journal of sports medicine*, *19*(1), 76-81.<br>Padasala, M., Joksimovic, M., Bruno, C., Melino, D., & Manzi, V. (2020). Muscle injuries in athletes. The relationship between H/Q ratio (hamstring/quadriceps ratio). *Ita J Sports Reh Po*, *7*(1), 1478-1498.<br>McGuire, B., & King, B. (2021). Neuromuscular risk factors for non-contact knee injury: a systematic review and meta-analysis. *medRxiv*, 2021-09.	general sporting population.
Fl_ex_ratio_asymmetry			Speculative.

Supplementary Table 10: Biomechanics variables

Feature Name	Explanation	References	Evidence Appraisal
Navicular_drop	The average navicular drop between left and right	Buist, I., Bredeweg, S. W., Lemmink, K. A., Van Mechelen, W., & Diercks, R. L. (2010). Predictors of running-related injuries in novice runners enrolled in a systematic training program: a prospective cohort study. *The American journal of sports medicine*, *38*(2), 273-280.<br>Bennett, J. E., Reinking, M. F., & Rauh, M. J. (2012). The relationship between isotonic plantar flexor endurance, navicular drop, and exercise-related leg pain in a cohort of collegiate cross-country runners. *International journal of sports physical therapy*, *7*(3), 267.	Multiple prospective studies showing correlation with RRIs.

		Raissi, G. R. D., Cherati, A. D. S., Mansoori, K. D., & Razi, M. D. (2009). The relationship between lower extremity alignment and Medial Tibial Stress Syndrome among non-professional athletes. *BMC Sports Science, Medicine and Rehabilitation*, *1*, 1-8.<br>Zifchock, R. A., Davis, I., Higginson, J., McCaw, S., & Royer, T. (2008). Side-to-side differences in overuse running injury susceptibility: a retrospective study. *Human movement science*, *27*(6), 888-902.<br>Christopher, S. M., McCullough, J., Snodgrass, S. J., & Cook, C. (2019). Do alterations in muscle strength, flexibility, range of motion, and alignment predict lower extremity injury in runners: a systematic review. *Archives of Physiotherapy*, *9*, 1-14.	

Navicular_drop_asymmetry	The absolute value of the difference between left and right navicular drop	Raissi, G. R. D., Cherati, A. D. S., Mansoori, K. D., & Razi, M. D. (2009). The relationship between lower extremity alignment and Medial Tibial Stress Syndrome among non-professional athletes. *BMC Sports Science, Medicine and Rehabilitation*, *1*, 1-8.	Prospective study shows runners sustaining MTSS have higher navicular drop asymmetry.
Q_angle	The average Q angle between left and right	Rauh, M. J., Koepsell, T. D., Rivara, F. P., Rice, S. G., & Margherita, A. J. (2007). Quadriceps angle and risk of injury among high school cross-country runners. *Journal of Orthopaedic & Sports Physical Therapy*, *37*(12), 725-733.<br>Puckree, T., Govender, A., Govender, K., & Naidoo, P. (2007). The quadriceps angle and the incidence of knee injury in Indian long-distance runners. *South African Journal of Sports Medicine*, *19*(1), 9-11.	Prospective studies show association with RRIs.

		Ellapen, T. J., Satyendra, S., Morris, J., & Van Heerden, H. J. (2013). Common running musculoskeletal injuries among recreational half-marathon runners in KwaZulu-Natal. *South African Journal of Sports Medicine*, *25*(2), 39-43.	
Q_angle_asymmetry	The absolute value of the difference between left and right Q angle	Rauh, M. J., Koepsell, T. D., Rivara, F. P., Rice, S. G., & Margherita, A. J. (2007). Quadriceps angle and risk of injury among high school cross-country runners. *Journal of Orthopaedic & Sports Physical Therapy*, *37*(12), 725-733.	Prospective study shows association with RRIs.
VILR_10[4]	Vertical impact loading rate during the 10km/h run	Davis, I. S., Bowser, B. J., & Mullineaux, D. R. (2016). Greater vertical impact loading in female runners with medically diagnosed injuries: a prospective	Ample prospective evidence on runners. However since features under

		investigation. *British journal of sports medicine*, *50*(14), 887-892.<br>Johnson, C. D., Tenforde, A. S., Outerleys, J., Reilly, J., & Davis, I. S. (2020). Impact-related ground reaction forces are more strongly associated with some running injuries than others. *The American journal of sports medicine*, *48*(12), 3072-3080.<br>Bredeweg, S. W., Kluitenberg, B., Bessem, B., & Buist, I. (2013). Differences in kinetic variables between injured and noninjured novice runners: a prospective cohort study. *Journal of Science and Medicine in Sport*, *16*(3), 205-210.	10km/h highly correlate with features under 12km/h, they are downgraded.
VALR_10[4]	Vertical average loading rate during the 10km/h run	Davis, I. S., Bowser, B. J., & Mullineaux, D. R. (2016). Greater vertical impact loading in female runners with	Same as above.

		medically diagnosed injuries: a prospective investigation. *British journal of sports medicine*, *50*(14), 887-892.<br>Johnson, C. D., Tenforde, A. S., Outerleys, J., Reilly, J., & Davis, I. S. (2020). Impact-related ground reaction forces are more strongly associated with some running injuries than others. *The American journal of sports medicine*, *48*(12), 3072-3080.<br>Bredeweg, S. W., Kluitenberg, B., Bessem, B., & Buist, I. (2013). Differences in kinetic variables between injured and noninjured novice runners: a prospective cohort study. *Journal of Science and Medicine in Sport*, *16*(3), 205-210.		

VILR_asymmetry_10	The absolute value of the difference between alternating steps on VILR normalised by the mean		Speculative.
VALR_asymmetry_10	The absolute value of the difference between alternating steps on VALR normalised by the mean		Speculative.
Impact_peak_10[5]	The average value of peak force experienced during each step during the 10km/h run	Davis, I. S., Bowser, B. J., & Mullineaux, D. R. (2016). Greater vertical impact loading in female runners with medically diagnosed injuries: a prospective investigation. *British journal of sports medicine*, *50*(14), 887-892.	Downgraded due to high correlation with feature under 12km/h.
Impact_peak_asymmetry_10	The absolute value of the difference between alternating steps on average value of peak force for the		Speculative

	10km/h run, normalised by the mean		
Flight_time_10[5]	Average amount of time during each step when the participant is not in contact with the ground for the 10km/h run.	Malisoux, L., Gette, P., Delattre, N., Urhausen, A., & Theisen, D. (2022). Spatiotemporal and ground-reaction force characteristics as risk factors for running-related injury: A secondary analysis of a randomized trial including 800+ recreational runners. *The American Journal of Sports Medicine*, *50*(2), 537-544.<br>Winter, S. C., Gordon, S., Brice, S. M., Lindsay, D., & Barrs, S. (2020). A multifactorial approach to overuse running injuries: a 1-year prospective study. *Sports Health*, *12*(3), 296-303.	Downgraded due to high correlation with feature under 12km/h.
Contact_time_10[5]	Average amount of time during each step when the participant is in	Malisoux, L., Gette, P., Delattre, N., Urhausen, A., & Theisen, D. (2022). Spatiotemporal and ground-reaction	Downgraded due to high correlation with

	contact with the ground for the 10km/h run.	force characteristics as risk factors for running-related injury: A secondary analysis of a randomized trial including 800+ recreational runners. *The American Journal of Sports Medicine*, *50*(2), 537-544.	feature under 12km/h.
Duty_factor_10	Ratio between contact time and stride time (flight time + contact time) for the 10km/h run.	Malisoux, L., Gette, P., Delattre, N., Urhausen, A., & Theisen, D. (2022). Spatiotemporal and ground-reaction force characteristics as risk factors for running-related injury: A secondary analysis of a randomized trial including 800+ recreational runners. *The American Journal of Sports Medicine*, *50*(2), 537-544.	Downgraded due to high correlation with feature under 12km/h.
Step_frequency_10	Number of steps per minutes during the 10km/h run	Winter, S. C., Gordon, S., Brice, S. M., Lindsay, D., & Barrs, S. (2020). A multifactorial approach to overuse running injuries: a 1-year prospective study. *Sports Health*, *12*(3), 296-303.	Downgraded due to high correlation with feature under 12km/h.

		Anderson, L. M., Martin, J. F., Barton, C. J., & Bonanno, D. R. (2022). What is the effect of changing running step rate on injury, performance and biomechanics? A systematic review and meta-analysis. *Sports Medicine-Open*, *8*(1), 112.	
Cadence_asymmetry_10	The absolute value of the difference between alternating steps on average time taken for each step for the 10km/h run, normalised by the mean.		Speculative.
Duty_factor_asymmetry_10	The absolute value of the difference between the duty factors of alternating steps for the 10km/h run, normalised by the mean.		Speculative.

VILR_12		Davis, I. S., Bowser, B. J., & Mullineaux, D. R. (2016). Greater vertical impact loading in female runners with medically diagnosed injuries: a prospective investigation. *British journal of sports medicine*, *50*(14), 887-892.<br>Johnson, C. D., Tenforde, A. S., Outerleys, J., Reilly, J., & Davis, I. S. (2020). Impact-related ground reaction forces are more strongly associated with some running injuries than others. *The American journal of sports medicine*, *48*(12), 3072-3080.<br>Bredeweg, S. W., Kluitenberg, B., Bessem, B., & Buist, I. (2013). Differences in kinetic variables between injured and noninjured novice runners: a prospective cohort	Prospective studies link VILR with RRIs, however since VILR correlates highly with VALR, it is downgraded.

		study. *Journal of Science and Medicine in Sport*, *16*(3), 205-210.	
VALR_12		Davis, I. S., Bowser, B. J., & Mullineaux, D. R. (2016). Greater vertical impact loading in female runners with medically diagnosed injuries: a prospective investigation. *British journal of sports medicine*, *50*(14), 887-892.<br>Johnson, C. D., Tenforde, A. S., Outerleys, J., Reilly, J., & Davis, I. S. (2020). Impact-related ground reaction forces are more strongly associated with some running injuries than others. *The American journal of sports medicine*, *48*(12), 3072-3080.<br>Bredeweg, S. W., Kluitenberg, B., Bessem, B., & Buist, I. (2013). Differences in kinetic variables between injured	VALR is chosen over VILR because within the prospective studies VALR present with higher effect sizes when both variables show significant correlation with RRIs.

		and noninjured novice runners: a prospective cohort study. *Journal of Science and Medicine in Sport*, *16*(3), 205-210.	
VILR_asymmetry_12			Speculative.
VALR_asymmetry_12			Speculative.
Impact_peak_12		Davis, I. S., Bowser, B. J., & Mullineaux, D. R. (2016). Greater vertical impact loading in female runners with medically diagnosed injuries: a prospective investigation. *British journal of sports medicine*, *50*(14), 887-892.	Prospective study shows correlations with RRIs.

Impact_peak_asymmetry_12			Speculative.
Flight_time_12	Average amount of time during each step when the participant is not in contact with the ground for the 12km/h run.	Malisoux, L., Gette, P., Delattre, N., Urhausen, A., & Theisen, D. (2022). Spatiotemporal and ground-reaction force characteristics as risk factors for running-related injury: A secondary analysis of a randomized trial including 800+ recreational runners. *The American Journal of Sports Medicine*, *50*(2), 537-544.<br>Winter, S. C., Gordon, S., Brice, S. M., Lindsay, D., & Barrs, S. (2020). A multifactorial approach to overuse running injuries: a 1-year prospective study. *Sports Health*, *12*(3), 296-303.	Downgraded due to high correlation with duty factor.

Contact_time_12	Average amount of time during each step when the participant is in contact with the ground for the 12km/h run.	Malisoux, L., Gette, P., Delattre, N., Urhausen, A., & Theisen, D. (2022). Spatiotemporal and ground-reaction force characteristics as risk factors for running-related injury: A secondary analysis of a randomized trial including 800+ recreational runners. *The American Journal of Sports Medicine*, *50*(2), 537-544.	Downgraded due to high correlation with duty factor.
Duty_factor_12	Ratio between contact time and stride time (flight time + contact time) for the 12km/h run.	Malisoux, L., Gette, P., Delattre, N., Urhausen, A., & Theisen, D. (2022). Spatiotemporal and ground-reaction force characteristics as risk factors for running-related injury: A secondary analysis of a randomized trial including 800+ recreational runners. *The American Journal of Sports Medicine*, *50*(2), 537-544.	Prospective study shows correlation with larger efffect sizes than flight time and contact time.
Step_frequency_12	Number of steps per minutes during the 12km/h run	Winter, S. C., Gordon, S., Brice, S. M., Lindsay, D., & Barrs, S. (2020). A multifactorial approach to overuse	Downgraded due to high correlation with

		running injuries: a 1-year prospective study. *Sports Health*, *12*(3), 296-303.<br>Anderson, L. M., Martin, J. F., Barton, C. J., & Bonanno, D. R. (2022). What is the effect of changing running step rate on injury, performance and biomechanics? A systematic review and meta-analysis. *Sports Medicine-Open*, *8*(1), 112.	flight time, contact time, and duty factor.
Cadence_asymmetry_12	The absolute value of the difference between alternating steps on average time taken for each step for the 12km/h run, normalised by the mean		Speculative.

Duty_factor_ asymmetry_ 12	The absolute value of the difference between the duty factors of alternating steps for the 12km/h run, normalised by the mean..		Speculative.
Alt_strike[6]	Alternating striker, meaning that for the most part of either the 10km/h or the 12km/h trial, the participant shows a rearfoot strike pattern with one leg and a forefoot strike pattern with the other leg		Speculative.

Supplementary Table 11: Nutrition variables

Feature Name	Explanation	References	Evidence Appraisal

Fat_intake_avg[7]	Average fat intake per day in grams	Gerlach, K. E., Burton, H. W., Dorn, J. M., Leddy, J. J., & Horvath, P. J. (2008). Fat intake and injury in female runners. *Journal of the International society of sports nutrition*, *5*, 1-8.	Prospective study shows link between fat intake and RRIs.
Fat_intake_BW[7] [9]	Average fat intake per day in grams / lean body mass		Speculative.
Fat_percentage_avg	Percentage of calories obtained from fat relative to total energy intake	Gerlach, K. E., Burton, H. W., Dorn, J. M., Leddy, J. J., & Horvath, P. J. (2008). Fat intake and injury in female runners. *Journal of the International society of sports nutrition*, *5*, 1-8.	Downgraded due to high correlation with fat intake.
Average_energy_availability[8]	(Caloric intake - exercise energy expenditure + resting metabolic rate during exercising hours) / lean body	Heikura, I. A., Uusitalo, A. L., Stellingwerff, T., Bergland, D., Mero, A. A., & Burke, L. M. (2018). Low energy availability is difficult to assess but outcomes have large impact on bone injury rates in elite distance	Prospective evidence seems to show LEAF-Q to be a better predictor than energy

	mass, averaged over each 3-day food diary	athletes. *International journal of sport nutrition and exercise metabolism*, *28*(4), 403-411.<br>Gerlach, K. E., Burton, H. W., Dorn, J. M., Leddy, J. J., & Horvath, P. J. (2008). Fat intake and injury in female runners. *Journal of the International society of sports nutrition*, *5*, 1-8.<br>Edama, M., Inaba, H., Hoshino, F., Natsui, S., Maruyama, S., & Omori, G. (2021). The relationship between the female athlete triad and injury rates in collegiate female athletes. *PeerJ*, *9*, e11092.<br>Close, G. L., Sale, C., Baar, K., & Bermon, S. (2019). Nutrition for the prevention and treatment of injuries in track and field athletes. *International journal of sport nutrition and exercise metabolism*, *29*(2), 189-197.	availability measured via food diary in predicting stress fracutres. Lack of evidence for EA’s direct correlation with sports injuries other than BSI.

		Griffin, K. L., Knight, K. B., Bass, M. A., & Valliant, M. W. (2021). Predisposing risk factors for stress fractures in collegiate cross-country runners. *The Journal of Strength & Conditioning Research*, *35*(1), 227-232. Curtis, L. (2016). Nutritional research may be useful in treating tendon injuries. *Nutrition*, *32*(6), 617-619.	
Protein_intake_BW	Average daily protein intake in g/kg lean body mass	Close, G. L., Sale, C., Baar, K., & Bermon, S. (2019). Nutrition for the prevention and treatment of injuries in track and field athletes. *International journal of sport nutrition and exercise metabolism*, *29*(2), 189-197. Curtis, L. (2016). Nutritional research may be useful in treating tendon injuries. *Nutrition*, *32*(6), 617-619.	Speculative.
Omega3_intake_BW	Average daily omega-3 intake in g/kg lean body mass	Close, G. L., Sale, C., Baar, K., & Bermon, S. (2019). Nutrition for the prevention and treatment of injuries in	Speculative.

		track and field athletes. *International journal of sport nutrition and exercise metabolism*, *29*(2), 189-197.	
vitaminD_intake_BW	Average daily vitamin D intake in in ug/kg lean body mass	Close, G. L., Sale, C., Baar, K., & Bermon, S. (2019). Nutrition for the prevention and treatment of injuries in track and field athletes. *International journal of sport nutrition and exercise metabolism*, *29*(2), 189-197.<br>Griffin, K. L., Knight, K. B., Bass, M. A., & Valliant, M. W. (2021). Predisposing risk factors for stress fractures in collegiate<br>Curtis, L. (2016). Nutritional research may be useful in treating tendon injuries. *Nutrition*, *32*(6), 617-619.cross-country runners. *The Journal of Strength & Conditioning Research*, *35*(1), 227-232.	Speculative.

vitaminC_intake_BW	Average daily vitamin C intake in in mg/kg lean body mass	Close, G. L., Sale, C., Baar, K., & Bermon, S. (2019). Nutrition for the prevention and treatment of injuries in track and field athletes. *International journal of sport nutrition and exercise metabolism*, *29*(2), 189-197. Curtis, L. (2016). Nutritional research may be useful in treating tendon injuries. *Nutrition*, *32*(6), 617-619.	Speculative.
vitaminE_intake_BW	Average daily vitamin E intake in in mg/kg lean body mass	Close, G. L., Sale, C., Baar, K., & Bermon, S. (2019). Nutrition for the prevention and treatment of injuries in track and field athletes. *International journal of sport nutrition and exercise metabolism*, *29*(2), 189-197.	Speculative.
Calcium_intake_BW	Average daily calcium intake in in mg/kg lean body mass	Close, G. L., Sale, C., Baar, K., & Bermon, S. (2019). Nutrition for the prevention and treatment of injuries in track and field athletes. *International journal of sport nutrition and exercise metabolism*, *29*(2), 189-197.	Speculative.

		Griffin, K. L., Knight, K. B., Bass, M. A., & Valliant, M. W. (2021). Predisposing risk factors for stress fractures in collegiate cross-country runners. *The Journal of Strength & Conditioning Research*, *35*(1), 227-232.	
copper_intake_BW	Average daily copper intake in in mg/kg lean body mass	Close, G. L., Sale, C., Baar, K., & Bermon, S. (2019). Nutrition for the prevention and treatment of injuries in track and field athletes. *International journal of sport nutrition and exercise metabolism*, *29*(2), 189-197.	Speculative.
iron_intake_BW	Average daily iron intake in in mg/kg lean body mass	Close, G. L., Sale, C., Baar, K., & Bermon, S. (2019). Nutrition for the prevention and treatment of injuries in track and field athletes. *International journal of sport nutrition and exercise metabolism*, *29*(2), 189-197.	Speculative.
Glycine_intake_BW	Average daily glycine intake in in g/kg lean body mass	Close, G. L., Sale, C., Baar, K., & Bermon, S. (2019). Nutrition for the prevention and treatment of injuries in	Speculative.

		track and field athletes. *International journal of sport nutrition and exercise metabolism*, *29*(2), 189-197.	
arginine_intake_BW	Average daily arginine intake in in g/kg lean body mass	Close, G. L., Sale, C., Baar, K., & Bermon, S. (2019). Nutrition for the prevention and treatment of injuries in track and field athletes. *International journal of sport nutrition and exercise metabolism*, *29*(2), 189-197. Curtis, L. (2016). Nutritional research may be useful in treating tendon injuries. *Nutrition*, *32*(6), 617-619.	Speculative.

Supplementary Table 12: Bone Scans[9] variables

Feature Name	Explanation	References	Evidence Appraisal
height	Average of height measured at all scans	Duffey, M. J., Martin, D. F., Cannon, D. W., Craven, T., & Messier, S. P. (2000). Etiologic factors associated with	Has prospective evidence but

		anterior knee pain in distance runners. *Medicine and science in sports and exercise*, *32*(11), 1825-1832.	downgraded due to high correlation with BMI.
Mass[9]		Shiotani, H., Mizokuchi, T., Yamashita, R., Naito, M., & Kawakami, Y. (2023). Influence of Body Mass on Running-Induced Changes in Mechanical Properties of Plantar Fascia. *The Journal of Strength & Conditioning Research*, *37*(11), e588-e592.	Downgraded due to high correlation with BMI.
BMI	mass / (height^2)	Buist, I., Bredeweg, S. W., Lemmink, K. A., Van Mechelen, W., & Diercks, R. L. (2010). Predictors of running-related injuries in novice runners enrolled in a systematic training program: a prospective cohort study. *The American journal of sports medicine*, *38*(2), 273-280.	Numerous prospective studies showing associations between BMI and RRIs.

		Theisen, D., Malisoux, L., Genin, J., Delattre, N., Seil, R., & Urhausen, A. (2014). Influence of midsole hardness of standard cushioned shoes on running-related injury risk. *British Journal of Sports Medicine*, *48*(5), 371-376.<br>Taunton, J. E., Ryan, M. B., Clement, D. B., McKenzie, D. C., Lloyd-Smith, D. R., & Zumbo, B. D. (2003). A prospective study of running injuries: the Vancouver Sun Run "In Training" clinics. *British journal of sports medicine*, *37*(3), 239-244.<br>Malisoux, L., Nielsen, R. O., Urhausen, A., & Theisen, D. (2015). A step towards understanding the mechanisms of running-related injuries. *Journal of Science and Medicine in Sport*, *18*(5), 523-528.	

		Taunton, J. E., Ryan, M. B., Clement, D. B., McKenzie, D. C., Lloyd-Smith, D. R., & Zumbo, B. D. (2002). A retrospective case-control analysis of 2002 running injuries. *British journal of sports medicine*, *36*(2), 95-101.<br>Van Leeuwen, K. D. B., Rogers, J., Winzenberg, T., & van Middelkoop, M. (2016). Higher body mass index is associated with plantar fasciopathy/'plantar fasciitis': systematic review and meta-analysis of various clinical and imaging risk factors. *British journal of sports medicine*, *50*(16), 972-981.	
Thigh_lean_mass[10]	Lean mass of the thigh region		Speculative.
Thigh_FFMI	Thigh lean mass / (height^2)		Speculative.

Lower_leg_lean_mass[10]	Lean mass of the lower leg region		Speculative.
Lower_leg_FFMI	Lower leg lean mass / (height^2)		Speculative.
Leg_lean_mass[10]	Thigh + lower leg lean mass	Liew, B. X., Zhu, X., Zhai, X., McErlain-Naylor, S. A., & McManus, C. (2024). Association between fat and fat-free body mass indices on shock attenuation during running. *Journal of Biomechanics*, *165*, 112025. Carbuhn, A. F., Yu, D., Magee, L. M., McCulloch, P. C., & Lambert, B. S. (2022). Anthropometric factors associated with bone stress injuries in collegiate distance runners: new risk metrics and screening tools?. *Orthopaedic Journal of Sports Medicine*, *10*(2), 23259671211070308.	Some evidence pointing to its associations with injury-related factors and with BSIs.

Leg_FFMI	Leg lean mass / (height^2)		Speculative.
Total_lean_mass	Lean mass of the entire body	Carbuhn, A. F., Yu, D., Magee, L. M., McCulloch, P. C., & Lambert, B. S. (2022). Anthropometric factors associated with bone stress injuries in collegiate distance runners: new risk metrics and screening tools?. *Orthopaedic Journal of Sports Medicine*, *10*(2), 23259671211070308.	Downgraded due to high correlation with leg lean mass.
Total_FFMI	Total lean mass / (height^2)	Domaradzki, J., & Koźlenia, D. (2022). The performance of body mass component indices in detecting risk of musculoskeletal injuries in physically active young men and women. *PeerJ*, *10*, e12745.	Downgraded due to high correlation with BMI.n
Calf_size	Calf muscle cross sectional area at 66% tibial length		Speculative.

BMD_spine	Bone mineral density of L1-L4 spine measured via DXA.	Rauh, M. J., Barrack, M., & Nichols, J. F. (2014). Associations between the female athlete triad and injury among high school runners. *International journal of sports physical therapy*, *9*(7), 948.<br>Rauh, M. J., Nichols, J. F., & Barrack, M. T. (2010). Relationships among injury and disordered eating, menstrual dysfunction, and low bone mineral density in high school athletes: a prospective study. *Journal of athletic training*, *45*(3), 243-252.	Prospective studies showing associations with RRIs.
BMD_hip	Bone mineral density of both hips measured via DXA.	Rauh, M. J., Barrack, M., & Nichols, J. F. (2014). Associations between the female athlete triad and injury among high school runners. *International journal of sports physical therapy*, *9*(7), 948.	Downgraded to to correlation with BMD_spine.

BMD_body	Bone mineral density of the entire body measured via DXA.	Rauh, M. J., Barrack, M., & Nichols, J. F. (2014). Associations between the female athlete triad and injury among high school runners. *International journal of sports physical therapy*, *9*(7), 948.<br>Rauh, M. J., Nichols, J. F., & Barrack, M. T. (2010). Relationships among injury and disordered eating, menstrual dysfunction, and low bone mineral density in high school athletes: a prospective study. *Journal of athletic training*, *45*(3), 243-252.	Downgraded to to correlation with BMD_spine.

Supplementary Table 13: Training (weekly questionnaire) variables

Feature Name	Explanation	References	Evidence Appraisal

Past_month_distance	Average running volume in km during the past 4 weeks	Hootman, J. M., Macera, C. A., Ainsworth, B. E., Martin, M., Addy, C. L., & Blair, S. N. (2002). Predictors of lower extremity injury among recreationally active adults. *Clinical Journal of Sport Medicine*, *12*(2), 99-106.<br>Lysholm, J., & Wiklander, J. (1987). Injuries in runners. *The American journal of sports medicine*, *15*(2), 168-171.<br>Walter, S. D., Hart, L. E., McIntosh, J. M., & Sutton, J. R. (1989). The Ontario cohort study of running-related injuries. *Archives of internal medicine*, *149*(11), 2561-2564.<br>Macera, C. A., Pate, R. R., Powell, K. E., Jackson, K. L., Kendrick, J. S., & Craven, T. E. (1989). Predicting lower-	Prospective studies show correlation between running distance and RRIs.

		extremity injuries among habitual runners. *Archives of internal medicine*, *149*(11), 2565-2568.<br>Koplan, J. P., Rothenberg, R. B., & Jones, E. L. (1995). The natural history of exercise: a 10-yr follow-up of a cohort of runners. *Medicine and Science in Sports and Exercise*, *27*(8), 1180-1184.	
Past_month_min	Average running volume in min during the past 4 weeks	Besomi, M., Leppe, J., Mauri-Stecca, M., Hooper, T., & Sizer, P. (2019). Training volume and previous injury as associated factors for running-related injuries by race distance: a cross-sectional study.<br>Rasmussen, C. H., Nielsen, R. O., Juul, M. S., & Rasmussen, S. (2013). Weekly running volume and risk of running-related injuries among marathon	Lack of prospective evidence, plus high correlation with distance volume, thus downgraded.

		runners. *International journal of sports physical therapy*, *8*(2), 111.<br>Malisoux, L., Nielsen, R. O., Urhausen, A., & Theisen, D. (2015). A step towards understanding the mechanisms of running-related injuries. *Journal of Science and Medicine in Sport*, *18*(5), 523-528.<br>Wen, D. Y. (2007). Risk factors for overuse injuries in runners. *Current sports medicine reports*, *6*(5), 307-313.	
Past_week_ratio	Total running volume in min during the past week divided by the total running volume in min during the week prior	Nielsen, R. Ø., Parner, E. T., Nohr, E. A., Sørensen, H., Lind, M., & Rasmussen, S. (2014). Excessive progression in weekly running distance and risk of running-related injuries: an association which varies	Downgraded due to high correlation with past_month_ratio. The latter is in line with the IOC's

		according to type of injury. *journal of orthopaedic & sports physical therapy*, *44*(10), 739-747.<br>Nielsen, R. O., Cederholm, P., Buist, I., Sørensen, H., Lind, M., & Rasmussen, S. (2013). Can GPS be used to detect deleterious progression in training volume among runners?. *The Journal of Strength & Conditioning Research*, *27*(6), 1471-1478.<br>Winter, S. C., Gordon, S., Brice, S. M., Lindsay, D., & Barrs, S. (2020). A multifactorial approach to overuse running injuries: a 1-year prospective study. *Sports Health*, *12*(3), 296-303.	definition of acute:chronic workload.
Past_month_ ratio	Total running volume in min during the past week divided by the	Dijkhuis, T. B., Otter, R., Aiello, M., Velthuijsen, H., & Lemmink, K. (2020). Increase in the acute: chronic workload ratio relates to injury risk in competitive	Prospective studies show correlation with RRIs.

	average running volume in min during the past 4 weeks	runners. *International journal of sports medicine*, *41*(11), 736-743.<br>Nakaoka, G., Barboza, S. D., Verhagen, E., Van Mechelen, W., & Hespanhol, L. (2021). The association between the acute: chronic workload ratio and running-related injuries in Dutch runners: a prospective cohort study. *Sports medicine*, *51*, 2437-2447.	
Past_month_volume_low	Average low intensity running volume in min during the past 4 weeks weeks		Speculative.
Past_week_ratio_low	Total low intensity running volume in min during the past week divided by the total running volume in min during the week prior		Speculative.

Past_month_ratio_low	Total low intensity running volume in min during the past week divided by the average running volume in min during the past 4 weeks		Speculative.
Past_month_volume_moderate			Speculative.
Past_week_ratio_moderate			Speculative.
Past_month_ratio_moderate			Speculative.

Past_month_volume_high			Speculative.
Past_week_ratio_high			Speculative.
Past_month_ratio_high			Speculative.
Past_month_volume_very_high			Speculative.
Past_week_ratio_very_high			Speculative.

Past_month_ratio_very_high			Speculative.
Past_month_calculated_volume	Average running volume during the past 4 weeks, calculated via “minutes under intensity * RPE”	Same as above with a different method of volume calculation	Speculative. Method of calculation is invented in this study.
Past_week_ratio_calculated_volume		Same as above with a different method of volume calculation	Speculative.
Past_month_ratio_calculated_volume		Same as above with a different method of volume calculation	Speculative.

Resistance_training_past_month	Total minutes of resistance training conducted during the past 4 weeks		Correlated with the past season feature.
Resistance_training_past_season	Total minutes of resistance training conducted during the past 12 weeks	Leppänen, M., Viiala, J., Kaikkonen, P., Tokola, K., Vasankari, T., Nigg, B. M., ... & Pasanen, K. (2024). Hip and core exercise programme prevents running-related overuse injuries in adult novice recreational runners: a three-arm randomised controlled trial (Run RCT). *British Journal of Sports Medicine*, *58*(13), 722-732.<br>Desai, P., Jungmalm, J., Börjesson, M., Karlsson, J., & Grau, S. (2023). Effectiveness of an 18-week general strength and foam-rolling intervention on running-	Downgraded due to correlation with total SC.

		related injuries in recreational runners. *Scandinavian Journal of Medicine & Science in Sports*, *33*(5), 766-775.<br>Mendez-Rebolledo, G., Figueroa-Ureta, R., Moya-Mura, F., Guzmán-Muñoz, E., Ramirez-Campillo, R., & Lloyd, R. S. (2021). The protective effect of neuromuscular training on the medial tibial stress syndrome in youth female track-and-field athletes: a clinical trial and cohort study. *Journal of sport rehabilitation*, *30*(7), 1019-1027.<br>Wu, H., Brooke-Wavell, K., Fong, D. T., Paquette, M. R., & Blagrove, R. C. (2024). Do Exercise-Based Prevention Programs Reduce Injury in Endurance Runners? A Systematic Review and Meta-Analysis. *Sports Medicine*, 1-19.	

Bodyweight_exercises_past_month	Total minutes of bodyweight exercises conducted during the past 4 weeks		Correlated with the past season feature.
Bodyweight_exercises_past_season	Total minutes of bodyweight exercises conducted during the past 12 weeks	Mendez-Rebolledo, G., Figueroa-Ureta, R., Moya-Mura, F., Guzmán-Muñoz, E., Ramirez-Campillo, R., & Lloyd, R. S. (2021). The protective effect of neuromuscular training on the medial tibial stress syndrome in youth female track-and-field athletes: a clinical trial and cohort study. *Journal of sport rehabilitation*, *30*(7), 1019-1027.<br>Wu, H., Brooke-Wavell, K., Fong, D. T., Paquette, M. R., & Blagrove, R. C. (2024). Do Exercise-Based Prevention Programs Reduce Injury in Endurance Runners? A	Downgraded due to correlation with total SC.

		Systematic Review and Meta-Analysis. *Sports Medicine*, 1-19.	
Core_stability_past_month	Total minutes of core stability exercises conducted during the past 4 weeks		Correlated with the past season feature.
Core_stability_past_season	Total minutes of core stability exercises conducted during the past 12 weeks	Leppänen, M., Viiala, J., Kaikkonen, P., Tokola, K., Vasankari, T., Nigg, B. M., ... & Pasanen, K. (2024). Hip and core exercise programme prevents running-related overuse injuries in adult novice recreational runners: a three-arm randomised controlled trial (Run RCT). *British Journal of Sports Medicine*, *58*(13), 722-732.<br>Mendez-Rebolledo, G., Figueroa-Ureta, R., Moya-Mura, F., Guzmán-Muñoz, E., Ramirez-Campillo, R., & Lloyd,	Downgraded due to correlation with total SC.

		R. S. (2021). The protective effect of neuromuscular training on the medial tibial stress syndrome in youth female track-and-field athletes: a clinical trial and cohort study. *Journal of sport rehabilitation*, *30*(7), 1019-1027.<br>Wu, H., Brooke-Wavell, K., Fong, D. T., Paquette, M. R., & Blagrove, R. C. (2024). Do Exercise-Based Prevention Programs Reduce Injury in Endurance Runners? A Systematic Review and Meta-Analysis. *Sports Medicine*, 1-19.	
Balance_training_past_month	Total minutes of balance training conducted during the past 4 weeks		Correlated with the past season feature.

Balance_training_past_season	Total minutes of balance training conducted during the past 12 weeks	Mendez-Rebolledo, G., Figueroa-Ureta, R., Moya-Mura, F., Guzmán-Muñoz, E., Ramirez-Campillo, R., & Lloyd, R. S. (2021). The protective effect of neuromuscular training on the medial tibial stress syndrome in youth female track-and-field athletes: a clinical trial and cohort study. *Journal of sport rehabilitation*, *30*(7), 1019-1027.<br>Wu, H., Brooke-Wavell, K., Fong, D. T., Paquette, M. R., & Blagrove, R. C. (2024). Do Exercise-Based Prevention Programs Reduce Injury in Endurance Runners? A Systematic Review and Meta-Analysis. *Sports Medicine*, 1-19.	Downgraded due to correlation with total SC.
plyometrics_past_month	Total minutes of plyometrics conducted during the past 4 weeks		Correlated with the past season feature.

plyometrics_past_season	Total minutes of plyometrics conducted during the past 12 weeks	Mendez-Rebolledo, G., Figueroa-Ureta, R., Moya-Mura, F., Guzmán-Muñoz, E., Ramirez-Campillo, R., & Lloyd, R. S. (2021). The protective effect of neuromuscular training on the medial tibial stress syndrome in youth female track-and-field athletes: a clinical trial and cohort study. *Journal of sport rehabilitation*, *30*(7), 1019-1027.<br>Wu, H., Brooke-Wavell, K., Fong, D. T., Paquette, M. R., & Blagrove, R. C. (2024). Do Exercise-Based Prevention Programs Reduce Injury in Endurance Runners? A Systematic Review and Meta-Analysis. *Sports Medicine*, 1-19.	Downgraded due to correlation with total SC.

drills_past_month	Total minutes of running technique drills conducted during the past 4 weeks		Correlated with the past season feature.
drills_past_season	Total minutes of running technique drills conducted during the past 12 weeks	Mendez-Rebolledo, G., Figueroa-Ureta, R., Moya-Mura, F., Guzmán-Muñoz, E., Ramirez-Campillo, R., & Lloyd, R. S. (2021). The protective effect of neuromuscular training on the medial tibial stress syndrome in youth female track-and-field athletes: a clinical trial and cohort study. *Journal of sport rehabilitation*, *30*(7), 1019-1027.<br>Wu, H., Brooke-Wavell, K., Fong, D. T., Paquette, M. R., & Blagrove, R. C. (2024). Do Exercise-Based Prevention Programs Reduce Injury in Endurance Runners? A	Downgraded due to correlation with total SC.

		Systematic Review and Meta-Analysis. *Sports Medicine*, 1-19.	
Circuit_training_past_month	Total minutes of circuit training conducted during the past 4 weeks		Correlated with the past season feature.
Circuit_training_past_season	Total minutes of circuit training conducted during the past 12 weeks		Speculative.
barefoot_past_month	Total minutes of barefoot exercises conducted during the past 4 weeks		Correlated with the past season feature.
barefoot_past_season	Total minutes of barefoot exercises conducted during the past 12 weeks		Speculative.
stretching_past_month	Total minutes of stretching or yoga conducted during the past 4 weeks		Correlated with the past season feature.

stretching_past_season	Total minutes of stretching or yoga conducted during the past 12 weeks	Weldon, S. M., & Hill, R. H. (2003). The efficacy of stretching for prevention of exercise-related injury: a systematic review of the literature. *Manual therapy*, *8*(3), 141-150.	Downgraded due to correlation with total SC.
SC_past_month	Total minutes of all of the above exercises conducted during the past 4 weeks		Correlated with the past season feature.
SC_past_season	Total minutes of all of the above exercises conducted during the past 12 weeks	Leppänen, M., Viiala, J., Kaikkonen, P., Tokola, K., Vasankari, T., Nigg, B. M., ... & Pasanen, K. (2024). Hip and core exercise programme prevents running-related overuse injuries in adult novice recreational runners: a three-arm randomised controlled trial (Run RCT). *British Journal of Sports Medicine*, *58*(13), 722-732.	Prospective intervention studies show supervised S&C has an effect on RRIs.

		Mendez-Rebolledo, G., Figueroa-Ureta, R., Moya-Mura, F., Guzmán-Muñoz, E., Ramirez-Campillo, R., & Lloyd, R. S. (2021). The protective effect of neuromuscular training on the medial tibial stress syndrome in youth female track-and-field athletes: a clinical trial and cohort study. *Journal of sport rehabilitation*, *30*(7), 1019-1027.<br>Desai, P., Jungmalm, J., Börjesson, M., Karlsson, J., & Grau, S. (2023). Effectiveness of an 18-week general strength and foam-rolling intervention on running-related injuries in recreational runners. *Scandinavian Journal of Medicine & Science in Sports*, *33*(5), 766-775.<br>Taddei, U. T., Matias, A. B., Duarte, M., & Sacco, I. C. (2020). Foot core training to prevent running-related	

		injuries: a survival analysis of a single-blind, randomized controlled trial. *The American journal of sports medicine*, *48*(14), 3610-3619.<br>Wu, H., Brooke-Wavell, K., Fong, D. T., Paquette, M. R., & Blagrove, R. C. (2024). Do Exercise-Based Prevention Programs Reduce Injury in Endurance Runners? A Systematic Review and Meta-Analysis. *Sports Medicine*, 1-19.	
Non_running_past_month	Total minutes of non-running (and non-S&C) exercises conducted during the past 4 weeks		Correlated with the past season feanture.
Non_running_past_season	Total minutes of all of non-running (and non-S&C) exercises conducted during the past 12 weeks	This is to control for total training volume by taking into account sports other than running: some people are pure runners, while some do other sports.	Included as a complementary

			factor for running training volume.
Past_month_injury	Whether injury was detected within the 4 weeks prior to the target week		It has been observed that participants who keep training after a recent injury tend to worsen the extent of the injury (re-injuries).
Tracking_period_injury	The participant's injury severity score summed up over all previous weeks / total number of previous weeks relative to current week (if multiple injuries occurred during a	Buist, I., Bredeweg, S. W., Lemmink, K. A., Van Mechelen, W., & Diercks, R. L. (2010). Predictors of running-related injuries in novice runners enrolled in a systematic training program: a prospective cohort	Ample evidence in prospective studies showing links between previous RRI and future RRI.

	week, severity score is calculated by highest region’s score + ((100 - highest region’s score) * second highest region’s score) / 100), and so on for additional regions)	study. *The American journal of sports medicine*, *38*(2), 273-280.<br>Theisen, D., Malisoux, L., Genin, J., Delattre, N., Seil, R., & Urhausen, A. (2014). Influence of midsole hardness of standard cushioned shoes on running-related injury risk. *British Journal of Sports Medicine*, *48*(5), 371-376.<br>Saragiotto, B. T., Yamato, T. P., Hespanhol Junior, L. C., Rainbow, M. J., Davis, I. S., & Lopes, A. D. (2014). What are the main risk factors for running-related injuries?. *Sports medicine*, *44*, 1153-1163.<br>Hulme, A., Nielsen, R. O., Timpka, T., Verhagen, E., & Finch, C. (2017). Risk and protective factors for middle- and long-distance running-related injury. *Sports Medicine*, *47*, 869-886.	

Supplementary Table 14: Genetics variables

rsID	Gene and Function	Risky Allele	References	Evidence Appraisal
rs3753841[11]	COL11A1, collagen XI fiber	Ref/alt	Alakhdar Mohmara, Y., Cook, J., Benítez-Martínez, J. C., McPeek, E. R., Aguilar, A. A., Olivas, E. S., & Hernandez-Sanchez, S. (2020). Influence of genetic factors in elbow tendon pathology: a case-control study. *Scientific Reports*, *10*(1), 6503.	Not RRI, weak evidence.
rs7528684[12]	FCRL3, immunoglobulin receptor	Alt	Salles, J. I., Lopes, L. R., Duarte, M. E. L., Morrissey, D., Martins, M. B., Machado, D. E., ... & Perini, J. A. (2018). Fc receptor-like 3 (− 169T> C) polymorphism increases the risk of tendinopathy in volleyball athletes: a case control study. *BMC medical genetics*, *19*, 1-10.	Function points more towards autoimmune conditions (rheumatoid arthritis) but linkage with lower limb tendinopathy.
rs57104447	CACNA1E, calcium voltage-	Alt	Kim, S. K., Roos, T. R., Roos, A. K., Kleimeyer, J. P., Ahmed, M. A., Goodlin, G. T., ... & Dragoo, J. L. (2017).	Function does not point to physical structrual

	gated channel subunit		Genome-wide association screens for Achilles tendon and ACL tears and tendinopathy. *PloS one*, *12*(3), e0170422.	integrity of tendon but linkage with lower limb tendinopathy. **rs183364169
rs1887632	SOST, provides instructions for making the protein sclerostin	Alt	Varley, I., Hughes, D. C., Greeves, J. P., Stellingwerff, T., Ranson, C., Fraser, W. D., & Sale, C. (2018). The association of novel polymorphisms with stress fracture injury in Elite Athletes: Further insights from the SFEA cohort. *Journal of science and medicine in sport*, *21*(6), 564-568.	Related to stress fracture, very few cases in current study.
rs4654760	TNAP, non-specific alkaline phosphatase	Ref/alt	Peach, C. A., Zhang, Y., Dunford, J. E., Brown, M. A., & Carr, A. J. (2007). Cuff tear arthropathy: evidence of functional variation in pyrophosphate metabolism	Function does not seem to be injury-related, reference is not RRI.

			genes. *Clinical Orthopaedics and Related Research (1976-2007)*, *462*, 67-72.	
rs1137101	LEPR, leptin receptor	Alt	Wang, Y., Meng, F., Wu, J., Long, H., Li, J., Wu, Z., ... & Xie, D. (2022). Associations between adipokines gene polymorphisms and knee osteoarthritis: a meta-analysis. *BMC musculoskeletal disorders*, *23*(1), 166.	Function does not seem to be injury-related, reference is not RRI.
rs4919510[13]	MIR608, microRNA-related	Ref/ref	Abrahams, Y., Laguette, M. J., Prince, S., & Collins, M. (2013). Polymorphisms within the COL5A1 3'-UTR that alters mRNA structure and the MIR608 gene are associated with Achilles tendinopathy. *Annals of human genetics*, *77*(3), 204-214.	Function does not seem to link to injury, but association found for achilles tendinopathy which is a common RRI.
rs1937810	MPP7, establishment of	Alt	Kim, S. K., Nguyen, C., Avins, A. L., & Abrams, G. D. (2021). Identification of Three Loci Associated with Achilles Tendon Injury Risk from a Genome-wide	Function does not seem to link to injury, but association found for

	epithelial cell polarity		Association Study. *Medicine and science in sports and exercise*, *53*(8), 1748.<br>Kim, S. K., Roos, T. R., Roos, A. K., Kleimeyer, J. P., Ahmed, M. A., Goodlin, G. T., ... & Dragoo, J. L. (2017). Genome-wide association screens for Achilles tendon and ACL tears and tendinopathy. *PloS one*, *12*(3), e0170422.<br>Kang, X., Tian, B., Zhang, L., Ge, Z., Zhao, Y., & Zhang, Y. (2019). Relationship of common variants in MPP7, TIMP2 and CASP8 genes with the risk of chronic achilles tendinopathy. *Scientific Reports*, *9*(1), 17627.	achilles tendinopathy which is a common RRI.
rs6481512	MPP7, establishment of	Alt	Kim, S. K., Nguyen, C., Avins, A. L., & Abrams, G. D. (2021). Identification of Three Loci Associated with Achilles Tendon Injury Risk from a Genome-wide	Function does not seem to link to injury, but association found for

	epithelial cell polarity		Association Study. *Medicine and science in sports and exercise*, *53*(8), 1748.	achilles tendinopathy which is a common RRI.
rs1249269	MPP7, establishment of epithelial cell polarity	Ref	Kim, S. K., Nguyen, C., Avins, A. L., & Abrams, G. D. (2021). Identification of Three Loci Associated with Achilles Tendon Injury Risk from a Genome-wide Association Study. *Medicine and science in sports and exercise*, *53*(8), 1748.	Function does not seem to link to injury, but association found for achilles tendinopathy which is a common RRI.
rs11225395	MMP8, matrix metalloproteinase, degradation of type I, II and III collagens	Ref	Godoy-Santos, A., Ortiz, R. T., Junior, R. M., Fernandes, T. D., & Santos, M. C. L. G. (2014). MMP-8 polymorphism is genetic marker to tendinopathy primary posterior tibial tendon. *Scandinavian journal of medicine & science in sports*, *24*(1), 220-223.<br>de Araujo Munhoz, F. B., Baroneza, J. E., Godoy-Santos, A., Fernandes, T. D., Branco, F. P., Alle, L. F., ... & Dos	Both function and references point to potential RRIs.

			Santos, M. C. L. G. (2016). Posterior tibial tendinopathy associated with matrix metalloproteinase 13 promoter genotype and haplotype. *The Journal of Gene Medicine*, *18*(11-12), 325-330.<br>Godoy-Santos, A., Cunha, M. V., Ortiz, R. T., Fernandes, T. D., Mattar Jr, R., & dos Santos, M. C. L. (2013). MMP-1 promoter polymorphism is associated with primary tendinopathy of the posterior tibial tendon. *Journal of Orthopaedic Research*, *31*(7), 1103-1107.	
rs1144393	MMP1, matrix metalloproteinase, breaks down the interstitial collagens	Alt	de Araujo Munhoz, F. B., Baroneza, J. E., Godoy-Santos, A., Fernandes, T. D., Branco, F. P., Alle, L. F., ... & Dos Santos, M. C. L. G. (2016). Posterior tibial tendinopathy associated with matrix metalloproteinase 13 promoter	Both function and references point to potential RRIs.

			genotype and haplotype. *The Journal of Gene Medicine*, *18*(11-12), 325-330. Baroneza, J. E., Godoy-Santos, A., Massa, B. F., de Araujo Munhoz, F. B., Fernandes, T. D., & dos Santos, M. C. L. G. (2014). MMP-1 promoter genotype and haplotype association with posterior tibial tendinopathy. *Gene*, *547*(2), 334-337.	
rs650108	MMP3, matrix metalloproteinase, degrades fibronectin, laminin, collagens III, IV, IX, and X	Alt/alt	Raleigh, S. M., Van der Merwe, L., & Ribbans, W. J. (2009). Variants within the MMP3 gene are associated with Achilles tendinopathy. *possible interaction with the COL5A1 gene*, *2009*, 43. Briški, N., Vrgoč, G., Knjaz, D., Janković, S., Ivković, A., Pećina, M., & Lauc, G. (2021). Association of the matrix metalloproteinase 3 (MMP3) single nucleotide	Both function and references point to potential RRIs.

			polymorphisms with tendinopathies: case-control study in high-level athletes. *International orthopaedics*, *45*, 1163-1168.	
rs679620 / rs591058*	MMP3, matrix metalloproteinase, degrades fibronectin, laminin, collagens III, IV, IX, and X	Alt	Raleigh, S. M., Van der Merwe, L., & Ribbans, W. J. (2009). Variants within the MMP3 gene are associated with Achilles tendinopathy. *possible interaction with the COL5A1 gene*, *2009*, 43.<br>Nie, G., Wen, X., Liang, X., Zhao, H., Li, Y., & Lu, J. (2019). Additional evidence supports association of common genetic variants in MMP3 and TIMP2 with increased risk of chronic Achilles tendinopathy susceptibility. *Journal of science and medicine in sport*, *22*(10), 1074-1078.	Both function and references point to potential RRIs.

			Briški, N., Vrgoč, G., Knjaz, D., Janković, S., Ivković, A., Pećina, M., & Lauc, G. (2021). Association of the matrix metalloproteinase 3 (MMP3) single nucleotide polymorphisms with tendinopathies: case-control study in high-level athletes. *International orthopaedics*, *45*, 1163-1168.<br>Figueiredo, E. A., Loyola, L. C., Belangero, P. S., Campos Ribeiro-dos-Santos, Â. K., Emanuel Batista Santos, S., Cohen, C., ... & Leal, M. F. (2020). Rotator cuff tear susceptibility is associated with variants in genes involved in tendon extracellular matrix homeostasis. *Journal of Orthopaedic Research®*, *38*(1), 192-201.	

rs2252070	MMP13, matrix metalloproteinase, cleaves type II collagen more efficiently than types I and III	Alt	de Araujo Munhoz, F. B., Baroneza, J. E., Godoy-Santos, A., Fernandes, T. D., Branco, F. P., Alle, L. F., ... & Dos Santos, M. C. L. G. (2016). Posterior tibial tendinopathy associated with matrix metalloproteinase 13 promoter genotype and haplotype. *The Journal of Gene Medicine*, *18*(11-12), 325-330.	Both function and references point to potential RRIs.
rs2306033	LRP4, LDL receptor protein	Ref/ref	Yanovich, R., Friedman, E., Milgrom, R., Oberman, B., Freedman, L., & Moran, D. S. (2012). Candidate gene analysis in Israeli soldiers with stress fractures. *Journal of Sports Science & Medicine*, *11*(1), 147.	Function does not seem to directly cause injury, reference is stress fracture, very few cases in current study.
rs2277268	LRP5, LDL receptor protein	Alt	Korvala, J., Hartikka, H., Pihlajamäki, H., Solovieva, S., Ruohola, J. P., Sahi, T., ... & Männikkö, M. (2010).	Function does not seem to directly cause injury, reference is stress

			Genetic predisposition for femoral neck stress fractures in military conscripts. *BMC genetics*, *11*, 1-9.	fracture, very few cases in current study.
rs4988321	LRP5, LDL receptor protein	Alt	Korvala, J., Hartikka, H., Pihlajamäki, H., Solovieva, S., Ruohola, J. P., Sahi, T., ... & Männikkö, M. (2010). Genetic predisposition for femoral neck stress fractures in military conscripts. *BMC genetics*, *11*, 1-9.	Function does not seem to directly cause injury, reference is stress fracture, very few cases in current study.
rs12574452	FGF3, fibroblast growth factor, embryonic development, cell growth, morphogenesis,	Alt/alt	da Rocha Motta, G., Amaral, M. V., Rezende, E., Pitta, R., dos Santos Vieira, T. C., Duarte, M. E. L., ... & Casado, P. L. (2014). Evidence of genetic variations associated with rotator cuff disease. *Journal of shoulder and elbow surgery*, *23*(2), 227-235. Tashjian, R. Z., Kim, S. K., Roche, M. D., Jones, K. B., & Teerlink, C. C. (2021). Genetic variants associated with	Function links to connective tissue repair, but references not with RRI.

	tissue repair, tumor growth		rotator cuff tearing utilizing multiple population-based genetic resources. *Journal of Shoulder and Elbow Surgery*, *30*(3), 520-531.	
rs11232681	RP11-664H7.2, pseudogene	Alt	Sood, R. F., Westenberg, R. F., Winograd, J. M., Eberlin, K. R., & Chen, N. C. (2020). Genetic risk of trigger finger: results of a genomewide association study. *Plastic and reconstructive surgery*, *146*(2), 165e-176e.	Function unclear and reference not RRI.
rs1718119	P2RX7, ATP-dependent lysis of macrophages	Ref/alt	Varley, I., Greeves, J. P., Sale, C., Friedman, E., Moran, D. S., Yanovich, R., ... & Gallagher, J. A. (2016). Functional polymorphisms in the P2X7 receptor gene are associated with stress fracture injury. *Purinergic signalling*, *12*, 103-113.	Function does not seem to directly cause injury, reference is stress fracture, very few cases in current study.

rs3751143	P2RX7, ATP-dependent lysis of macrophages	Ref/alt	Varley, I., Greeves, J. P., Sale, C., Friedman, E., Moran, D. S., Yanovich, R., ... & Gallagher, J. A. (2016). Functional polymorphisms in the P2X7 receptor gene are associated with stress fracture injury. *Purinergic signalling*, *12*, 103-113.	Function does not seem to directly cause injury, reference is stress fracture, very few cases in current study.
rs1544410	VDR, vitamin D receptor	Ref	Chatzipapas, C., Boikos, S., Drosos, G. I., Kazakos, K., Tripsianis, G., Serbis, A., ... & Stratakis, C. A. (2009). Polymorphisms of the vitamin D receptor gene and stress fractures. *Hormone and Metabolic Research*, *41*(08), 635-640.	Function and reference both point to stress fracture, very few cases in current study.
rs2228570	VDR, vitamin D receptor	Ref	Chatzipapas, C., Boikos, S., Drosos, G. I., Kazakos, K., Tripsianis, G., Serbis, A., ... & Stratakis, C. A. (2009). Polymorphisms of the vitamin D receptor gene and	Function and reference both point to stress fracture, very few cases in current study.

			stress fractures. *Hormone and Metabolic Research*, *41*(08), 635-640.	
rs4328262	VDR, vitamin D receptor	Ref/ref, Alt/alt	Yanovich, R., Friedman, E., Milgrom, R., Oberman, B., Freedman, L., & Moran, D. S. (2012). Candidate gene analysis in Israeli soldiers with stress fractures. *Journal of Sports Science & Medicine*, *11*(1), 147.	Function and reference both point to stress fracture, very few cases in current study.
rs1021188	RANKL, osteoclastogenesis	Alt/alt	Varley, I., Hughes, D. C., Greeves, J. P., Stellingwerff, T., Ranson, C., Fraser, W. D., & Sale, C. (2015). RANK/RANKL/OPG pathway: genetic associations with stress fracture period prevalence in elite athletes. *Bone*, *71*, 131-136.	Function and reference both point to stress fracture, very few cases in current study.
rs74544784	KLHL1, actin-organizing proteins	Alt	Sood, R. F., Westenberg, R. F., Winograd, J. M., Eberlin, K. R., & Chen, N. C. (2020). Genetic risk of trigger finger:	Function seems close to musculoskeletal problems

			results of a genomewide association study. *Plastic and reconstructive surgery*, *146*(2), 165e-176e.	but reference not with RRI. **rs12429486
rs12429486 / rs113732656 / rs113033795 / rs111237638 / rs113796757 / rs59988404 / rs113390517 / rs12429555	KLHL1, actin-organizing proteins	Alt	Sood, R. F., Westenberg, R. F., Winograd, J. M., Eberlin, K. R., & Chen, N. C. (2020). Genetic risk of trigger finger: results of a genomewide association study. *Plastic and reconstructive surgery*, *146*(2), 165e-176e.	Function seems close to musculoskeletal problems but reference not with RRI.

/ rs12429576 / rs12428036 / rs76743567 / rs117409997 / rs74092661*				
rs78391032	KLHL1, actin-organizing proteins	Alt	Sood, R. F., Westenberg, R. F., Winograd, J. M., Eberlin, K. R., & Chen, N. C. (2020). Genetic risk of trigger finger: results of a genomewide association study. *Plastic and reconstructive surgery*, *146*(2), 165e-176e.	Function seems close to musculoskeletal problems but reference not with RRI. **rs12429486
rs77569527	KLHL1, actin-organizing proteins	Alt	Sood, R. F., Westenberg, R. F., Winograd, J. M., Eberlin, K. R., & Chen, N. C. (2020). Genetic risk of trigger finger:	Function seems close to musculoskeletal problems

			results of a genomewide association study. *Plastic and reconstructive surgery*, *146*(2), 165e-176e.	but reference not with RRI. **rs12429486
rs117544024	KLHL1, actin-organizing proteins	Alt	Sood, R. F., Westenberg, R. F., Winograd, J. M., Eberlin, K. R., & Chen, N. C. (2020). Genetic risk of trigger finger: results of a genomewide association study. *Plastic and reconstructive surgery*, *146*(2), 165e-176e.	Function seems close to musculoskeletal problems but reference not with RRI. **rs12429486
rs4454832	SOX21, encode a family of DNA-binding proteins	Ref	Kim, S. K., Nguyen, C., Avins, A. L., & Abrams, G. D. (2021). Identification of Three Loci Associated with Achilles Tendon Injury Risk from a Genome-wide Association Study. *Medicine and science in sports and exercise*, *53*(8), 1748.	Function does not relate to RRI, but reference on Achilles tendon injury.
rs912336	STK24, encodes a serine/threonine protein kinase	Ref	Kim, S. K., Nguyen, C., Jones, K. B., & Tashjian, R. Z. (2021). A genome-wide association study for shoulder	Neither function nor reference directly point to RRI.

			impingement and rotator cuff disease. *Journal of shoulder and elbow surgery*, *30*(9), 2134-2145.	
rs3218791 / rs8003305*	POLE2, DNA polymerase epsilon, involved in DNA repair and replication	Alt	Sood, R. F., Westenberg, R. F., Winograd, J. M., Eberlin, K. R., & Chen, N. C. (2020). Genetic risk of trigger finger: results of a genomewide association study. *Plastic and reconstructive surgery*, *146*(2), 165e-176e.	Neither function nor reference directly point to RRI.
rs2761884	BMP4, bone morphogentic protein, heart development and adipogenesis	Alt	Salles, J. I., Amaral, M. V., Aguiar, D. P., Lira, D. A., Quinelato, V., Bonato, L. L., ... & Casado, P. L. (2015). BMP4 and FGF3 haplotypes increase the risk of tendinopathy in volleyball athletes. *Journal of science and medicine in sport*, *18*(2), 150-155.	Function does not point to physical structrual integrity of tendon but linkage with lower limb tendinopathy.
rs4986938	ESR2, estrogen receptor	Alt	Nogara, P. R. B., Godoy-Santos, A. L., Fonseca, F. C. P., Cesar-Netto, C., Carvalho, K. C., Baracat, E. C., ... &	Both function and reference seem to link to

			Santos, M. C. L. (2020). Association of estrogen receptor β polymorphisms with posterior tibial tendon dysfunction. *Molecular and cellular biochemistry*, *471*, 63-69.	common RRIs especially in females.
rs911263	RAD51B, DNA repair by homologous recombination	Alt	Zhi, L., Yao, S., Ma, W., Zhang, W., Chen, H., Li, M., & Ma, J. (2017). Polymorphisms of RAD51B are associated with rheumatoid arthritis and erosion in rheumatoid arthritis patients. *Scientific Reports*, *7*(1), 45876.	Neither function nor reference directly point to RRI.
rs2525504	RAD51B, DNA repair by homologous recombination	Alt	Zhi, L., Yao, S., Ma, W., Zhang, W., Chen, H., Li, M., & Ma, J. (2017). Polymorphisms of RAD51B are associated with rheumatoid arthritis and erosion in rheumatoid arthritis patients. *Scientific Reports*, *7*(1), 45876.	Neither function nor reference directly point to RRI.

rs17756404	RAD51B, DNA repair by homologous recombination	Ref	Zhi, L., Yao, S., Ma, W., Zhang, W., Chen, H., Li, M., & Ma, J. (2017). Polymorphisms of RAD51B are associated with rheumatoid arthritis and erosion in rheumatoid arthritis patients. *Scientific Reports*, *7*(1), 45876.	Neither function nor reference directly point to RRI.
rs4903399	ESRRB, estrogen-realted receptor beta	Ref/ref	da Rocha Motta, G., Amaral, M. V., Rezende, E., Pitta, R., dos Santos Vieira, T. C., Duarte, M. E. L., ... & Casado, P. L. (2014). Evidence of genetic variations associated with rotator cuff disease. *Journal of shoulder and elbow surgery*, *23*(2), 227-235.<br>Bonato, L. L., Quinelato, V., Amaral, M. V. G., de Souza, F. N., Lobo, J. C., Aguiar, D. P., ... & Casado, P. L. (2016). ESRRB polymorphisms are associated with comorbidity of temporomandibular disorders and rotator cuff	Neither function nor reference directly point to RRI ('estrogen-related' receptor is not actually an estrogen receptor).

			disease. *International Journal of Oral and Maxillofacial Surgery*, *45*(3), 323-331.	
rs10132091	ESRRB, estrogen-realted receptor beta	Ref	Bonato, L. L., Quinelato, V., Amaral, M. V. G., de Souza, F. N., Lobo, J. C., Aguiar, D. P., ... & Casado, P. L. (2016). ESRRB polymorphisms are associated with comorbidity of temporomandibular disorders and rotator cuff disease. *International Journal of Oral and Maxillofacial Surgery*, *45*(3), 323-331.	Neither function nor reference directly point to RRI.
rs17583842	ESRRB, estrogen-realted receptor beta	Alt	Teerlink, C. C., Cannon-Albright, L. A., & Tashjian, R. Z. (2015). Significant association of full-thickness rotator cuff tears and estrogen-related receptor-β (ESRRB). *Journal of shoulder and elbow surgery*, *24*(2), e31-e35.	Neither function nor reference directly point to RRI.

rs1676303	ESRRB, estrogen-realted receptor beta	Ref	da Rocha Motta, G., Amaral, M. V., Rezende, E., Pitta, R., dos Santos Vieira, T. C., Duarte, M. E. L., ... & Casado, P. L. (2014). Evidence of genetic variations associated with rotator cuff disease. *Journal of shoulder and elbow surgery*, *23*(2), 227-235. Bonato, L. L., Quinelato, V., Amaral, M. V. G., de Souza, F. N., Lobo, J. C., Aguiar, D. P., ... & Casado, P. L. (2016). ESRRB polymorphisms are associated with comorbidity of temporomandibular disorders and rotator cuff disease. *International Journal of Oral and Maxillofacial Surgery*, *45*(3), 323-331.	Neither function nor reference directly point to RRI.
rs11629171	SERPINA6, serine proteinase inhibitor,	Alt	Yanovich, R., Friedman, E., Milgrom, R., Oberman, B., Freedman, L., & Moran, D. S. (2012). Candidate gene	Function does not seem to link to RRIs, and reference

	glucorticoids and progestins transport		analysis in Israeli soldiers with stress fractures. *Journal of Sports Science & Medicine*, *11*(1), 147.	is in stress fracture, too few cases in current study.
rs2281518	SERPINA6, serine proteinase inhibitor, glucorticoids and progestins transport	Ref/alt	Yanovich, R., Friedman, E., Milgrom, R., Oberman, B., Freedman, L., & Moran, D. S. (2012). Candidate gene analysis in Israeli soldiers with stress fractures. *Journal of Sports Science & Medicine*, *11*(1), 147.	Function does not seem to link to RRIs, and reference is in stress fracture, too few cases in current study.
rs2285053	MMP2, matrix metalloproteinase, gelatinase A, type IV collagenase	Alt	Figueiredo, E. A., Loyola, L. C., Belangero, P. S., Campos Ribeiro-dos-Santos, Â. K., Emanuel Batista Santos, S., Cohen, C., ... & Leal, M. F. (2020). Rotator cuff tear susceptibility is associated with variants in genes involved in tendon extracellular matrix	Neither function nor reference directly point to RRI.

			homeostasis. *Journal of Orthopaedic Research®*, *38*(1), 192-201.	
rs71404070	CDH8, cell adhesion	Alt	Roos, T. R., Roos, A. K., Avins, A. L., Ahmed, M. A., Kleimeyer, J. P., Fredericson, M., ... & Kim, S. K. (2017). Genome-wide association study identifies a locus associated with rotator cuff injury. *PLoS One*, *12*(12), e0189317.	Neither function nor reference directly point to RRI.
rs62051384	WWP2, protein ubiquitination	Alt	Kim, S. K., Ioannidis, J. P., Ahmed, M. A., Avins, A. L., Kleimeyer, J. P., Fredericson, M., & Dragoo, J. L. (2018). Two genetic variants associated with plantar fascial disorders. *International journal of sports medicine*, *39*(04), 314-321.	Function doesn't seem directly linked to injuries, but reference on common RRI.
rs4362400	VAT1L, oxidoreductase	Alt	Rodas, G., Osaba, L., Arteta, D., Pruna, R., Fernández, D., & Lucia, A. (2019). Genomic prediction of	Function doesn't seem directly linked to injuries,

	activity and zinc ion binding activity		tendinopathy risk in elite team sports. *International Journal of Sports Physiology and Performance*, *15*(4), 489-495.	but reference on tendinopathy.
rs710079	MPG, alkylbase DNA N-glycosylase activity	Ref	Chen, S. Y., Wan, L., Huang, C. M., Huang, Y. C., Sheu, J. J. C., Lin, Y. J., ... & Tsai, F. J. (2010). Genetic polymorphisms of the DNA repair gene MPG may be associated with susceptibility to rheumatoid arthritis. *Journal of applied genetics*, *51*, 519-521.	Neither function nor reference directly point to RRI.
rs2858056	MPG, alkylbase DNA N-glycosylase activity	Ref/ref	Chen, S. Y., Wan, L., Huang, C. M., Huang, Y. C., Sheu, J. J. C., Lin, Y. J., ... & Tsai, F. J. (2010). Genetic polymorphisms of the DNA repair gene MPG may be associated with susceptibility to rheumatoid arthritis. *Journal of applied genetics*, *51*, 519-521.	Neither function nor reference directly point to RRI.

rs2586488	COL1A1, pro-alpha1 chains of type I collagen	Ref/ref	Korvala, J., Hartikka, H., Pihlajamäki, H., Solovieva, S., Ruohola, J. P., Sahi, T., ... & Männikkö, M. (2010). Genetic predisposition for femoral neck stress fractures in military conscripts. *BMC genetics*, *11*, 1-9.	Function seems tendon-related but reference only with stress fracture, too few cases in current study.
rs1800012	COL1A1, pro-alpha1 chains of type I collagen	Ref	Wang, C., Li, H., Chen, K., Wu, B., & Liu, H. (2017). Association of polymorphisms rs1800012 in COL1A1 with sports-related tendon and ligament injuries: a meta-analysis. *Oncotarget*, *8*(16), 27627.<br>Leźnicka, K., Żyżniewska-Banaszak, E., Gębska, M., Machoy-Mokrzyńska, A., Krajewska-Pędzik, A., Maciejewska-Skrendo, A., & Leońska-Duniec, A. (2021). Interactions between gene variants within the COL1A1 and COL5A1 genes and musculoskeletal injuries in physically active Caucasian. *Genes*, *12*(7), 1056.	Both function and referenced injuries seem close to RRI.

rs820218	SAP30BP, modulation by host of symbiont transcription	Ref	Tashjian, R. Z., Granger, E. K., Farnham, J. M., Cannon-Albright, L. A., & Teerlink, C. C. (2016). Genome-wide association study for rotator cuff tears identifies two significant single-nucleotide polymorphisms. *Journal of shoulder and elbow surgery*, *25*(2), 174-179.	Neither function nor reference directly point to RRI.
rs2277698	TIMP2, natural inhibitors of the matrix metalloproteinase s	Alt	Tashjian, R. Z., Kim, S. K., Roche, M. D., Jones, K. B., & Teerlink, C. C. (2021). Genetic variants associated with rotator cuff tearing utilizing multiple population-based genetic resources. *Journal of Shoulder and Elbow Surgery*, *30*(3), 520-531. Figueiredo, E. A., Loyola, L. C., Belangero, P. S., Campos Ribeiro-dos-Santos, Â. K., Emanuel Batista Santos, S., Cohen, C., ... & Leal, M. F. (2020). Rotator cuff tear susceptibility is associated with variants in	Function links to tissue homeostasis, but reference not with RRI.

			genes involved in tendon extracellular matrix homeostasis. *Journal of Orthopaedic Research®*, *38*(1), 192-201.	
rs4789932	TIMP2, natural inhibitors of the matrix metalloproteinases	Ref	El Khoury, L., Posthumus, M., Collins, M., Handley, C. J., Cook, J., & Raleigh, S. M. (2013). Polymorphic variation within the ADAMTS2, ADAMTS14, ADAMTS5, ADAM12 and TIMP2 genes and the risk of Achilles tendon pathology: a genetic association study. *Journal of science and medicine in sport*, *16*(6), 493-498.<br>El Khoury, L., Ribbans, W. J., & Raleigh, S. M. (2016). MMP3 and TIMP2 gene variants as predisposing factors for Achilles tendon pathologies: Attempted replication study in a British case–control cohort. *Meta gene*, *9*, 52-55.	Function on tissue homeostasis and references on Achilles tendinopathy.

			Kim, S. K., Roos, T. R., Roos, A. K., Kleimeyer, J. P., Ahmed, M. A., Goodlin, G. T., ... & Dragoo, J. L. (2017). Genome-wide association screens for Achilles tendon and ACL tears and tendinopathy. *PloS one*, *12*(3), e0170422.<br>Kang, X., Tian, B., Zhang, L., Ge, Z., Zhao, Y., & Zhang, Y. (2019). Relationship of common variants in MPP7, TIMP2 and CASP8 genes with the risk of chronic achilles tendinopathy. *Scientific Reports*, *9*(1), 17627.<br>Nie, G., Wen, X., Liang, X., Zhao, H., Li, Y., & Lu, J. (2019). Additional evidence supports association of common genetic variants in MMP3 and TIMP2 with increased risk of chronic Achilles tendinopathy	

			susceptibility. *Journal of science and medicine in sport*, *22*(10), 1074-1078.	
rs3018362	TNFRSF11A, tumor necrosis factor receptor	Ref	Varley, I., Hughes, D. C., Greeves, J. P., Stellingwerff, T., Ranson, C., Fraser, W. D., & Sale, C. (2015). RANK/RANKL/OPG pathway: genetic associations with stress fracture period prevalence in elite athletes. *Bone*, *71*, 131-136.	Function does not link to RRI and reference on stress fracture, too few cases in current study.
rs1800470	TGFB1, transforming growth factor beta 1	Alt/alt	Figueiredo, E. A., Loyola, L. C., Belangero, P. S., Campos Ribeiro-dos-Santos, Â. K., Emanuel Batista Santos, S., Cohen, C., ... & Leal, M. F. (2020). Rotator cuff tear susceptibility is associated with variants in genes involved in tendon extracellular matrix homeostasis. *Journal of Orthopaedic Research®*, *38*(1), 192-201.	Neither function nor reference directly point to RRI.

rs1800469	TGFB1, transforming growth factor beta 1	Alt/alt	Figueiredo, E. A., Loyola, L. C., Belangero, P. S., Campos Ribeiro-dos-Santos, Â. K., Emanuel Batista Santos, S., Cohen, C., ... & Leal, M. F. (2020). Rotator cuff tear susceptibility is associated with variants in genes involved in tendon extracellular matrix homeostasis. *Journal of Orthopaedic Research®*, *38*(1), 192-201.	Neither function nor reference directly point to RRI.
rs25487	XRCC1, repair of DNA single-strand breaks	Ref	Mohamed, R. H., Amal, S., El-Shahawy, E. E., & Galil, S. M. A. (2016). Association of XRCC1 and OGG1 DNA repair gene polymorphisms with rheumatoid arthritis in Egyptian patients. *Gene*, *578*(1), 112-116.	Neither function nor reference directly point to RRI.
rs25489	XRCC1, repair of DNA single-strand breaks	Alt	Mohamed, R. H., Amal, S., El-Shahawy, E. E., & Galil, S. M. A. (2016). Association of XRCC1 and OGG1 DNA	Neither function nor reference directly point to RRI.

			repair gene polymorphisms with rheumatoid arthritis in Egyptian patients. *Gene*, *578*(1), 112-116.	
rs1045485	CASP8, execution-phase of cell apoptosis	Alt	Nell, E. M., Van Der Merwe, L., Cook, J., Handley, C. J., Collins, M., & September, A. V. (2012). The apoptosis pathway and the genetic predisposition to Achilles tendinopathy. *Journal of Orthopaedic Research*, *30*(11), 1719-1724.<br>Kim, S. K., Roos, T. R., Roos, A. K., Kleimeyer, J. P., Ahmed, M. A., Goodlin, G. T., ... & Dragoo, J. L. (2017). Genome-wide association screens for Achilles tendon and ACL tears and tendinopathy. *PloS one*, *12*(3), e0170422.	Evidence points to Achilles tendinopathy but function does not directly link to RRI.

rs2289360	EMILIN1, associates with elastic fibers at the interface between elastin and microfibrils	Alt/alt	Hall, E. C., Baumert, P., Larruskain, J., Gil, S. M., Lekue, J. A., Rienzi, E., ... & Erskine, R. M. (2022). The genetic association with injury risk in male academy soccer players depends on maturity status. *Scandinavian Journal of Medicine & Science in Sports*, *32*(2), 338-350.	Neither function nor reference directly point to RRI.
rs143383	GDF5, growth differentiation factor	Alt	Zhao, L., Chang, Q., Huang, T., & Huang, C. (2016). Prospective cohort study of the risk factors for stress fractures in Chinese male infantry recruits. *Journal of International Medical Research*, *44*(4), 787-795. Posthumus, M., Collins, M., Cook, J., Handley, C. J., Ribbans, W. J., Smith, R. K., ... & Raleigh, S. M. (2010). Components of the transforming growth factor-β family and the pathogenesis of human Achilles tendon	Function does not seem to link to RRI but reference contains Achilles tendinopathy.

			pathology—a genetic association study. *Rheumatology*, *49*(11), 2090-2097.<br>Vaes, R. B. A., Rivadeneira, F., Kerkhof, J. M., Hofman, A., Pols, H. A. P., Uitterlinden, A. G., & van Meurs, J. B. J. (2009). Genetic variation in the GDF5 region is associated with osteoarthritis, height, hip axis length and fracture risk: the Rotterdam study. *Annals of the rheumatic diseases*, *68*(11), 1754-1760.<br>Ge, W., Mu, J., & Huang, C. (2014). The GDF5 SNP is associated with meniscus injury and function recovery in male Chinese soldiers. *International journal of sports medicine*, *35*(07), 625-628.	

rs17576	MMP9, degrades type IV and V collagens	Alt	Figueiredo, E. A., Loyola, L. C., Belangero, P. S., Campos Ribeiro-dos-Santos, Â. K., Emanuel Batista Santos, S., Cohen, C., ... & Leal, M. F. (2020). Rotator cuff tear susceptibility is associated with variants in genes involved in tendon extracellular matrix homeostasis. *Journal of Orthopaedic Research®*, *38*(1), 192-201.	Function seems relevant but reference not with RRI.
rs183364169	CDCP1, tumor invasion and metastasis	Alt	Kim, S. K., Nguyen, C., Avins, A. L., & Abrams, G. D. (2021). Identification of Three Loci Associated with Achilles Tendon Injury Risk from a Genome-wide Association Study. *Medicine and science in sports and exercise*, *53*(8), 1748.	Function does not seem relevant but reference with Achilles tendon injury. **rs149047058

rs11177	GNL3, may be involved in tumorigenesis and stem cell proliferation	Alt	Liu, B., Cheng, H., Ma, W., Gong, F., Wang, X., Duan, N., & Dang, X. (2018). Common variants in the GNL3 contribute to the increasing risk of knee osteoarthritis in Han Chinese population. *Scientific reports*, *8*(1), 9610.	Neither function nor reference directly point to RRI.
rs6617	GNL3, may be involved in tumorigenesis and stem cell proliferation	Alt	Liu, B., Cheng, H., Ma, W., Gong, F., Wang, X., Duan, N., & Dang, X. (2018). Common variants in the GNL3 contribute to the increasing risk of knee osteoarthritis in Han Chinese population. *Scientific reports*, *8*(1), 9610.	Neither function nor reference directly point to RRI.
rs3219008	OGG1, excision of 8-oxoguanine, a mutagenic base byproduct	Ref/alt	Mohamed, R. H., Amal, S., El-Shahawy, E. E., & Galil, S. M. A. (2016). Association of XRCC1 and OGG1 DNA repair gene polymorphisms with rheumatoid arthritis in Egyptian patients. *Gene*, *578*(1), 112-116.	Neither function nor reference directly point to RRI.

rs13107325	SLC39A8, cellular import of zinc at the onset of inflammation	Alt	Kim, S. K., Nguyen, C., Jones, K. B., & Tashjian, R. Z. (2021). A genome-wide association study for shoulder impingement and rotator cuff disease. *Journal of shoulder and elbow surgery*, *30*(9), 2134-2145.	Neither function nor reference directly point to RRI.
rs60713544	TRIML1, function of the encoded protein has not been determined	Alt	Kim, S. K., Roos, T. R., Roos, A. K., Kleimeyer, J. P., Ahmed, M. A., Goodlin, G. T., ... & Dragoo, J. L. (2017). Genome-wide association screens for Achilles tendon and ACL tears and tendinopathy. *PloS one*, *12*(3), e0170422.	Neither function nor reference directly point to RRI.
rs2305948	KDR, endothelial proliferation, survival, migration, tubular	Ref/ref	Salles, J. I., Duarte, M. E. L., Guimarães, J. M., Lopes, L. R., Vilarinho Cardoso, J., Aguiar, D. P., ... & Perini, J. A. (2016). Vascular endothelial growth factor receptor-2 polymorphisms have protective effect against the	Function does not seem to be relevant but reference covers lower limb tendinopathy.

	morphogenesis and sprouting		development of tendinopathy in volleyball athletes. *PLoS One*, *11*(12), e0167717.	
rs145648292	C5orf63, open reading frame, glutaredoxin-like protein	Alt	Kim, S. K., Nguyen, C., Jones, K. B., & Tashjian, R. Z. (2021). A genome-wide association study for shoulder impingement and rotator cuff disease. *Journal of shoulder and elbow surgery*, *30*(9), 2134-2145.	Neither function nor reference directly point to RRI.
rs4244032	NR3C1, glucocorticoid receptor, inflammatory responses	Ref/ref, Alt/alt	Yanovich, R., Friedman, E., Milgrom, R., Oberman, B., Freedman, L., & Moran, D. S. (2012). Candidate gene analysis in Israeli soldiers with stress fractures. *Journal of Sports Science & Medicine*, *11*(1), 147.	Function does not seem relevant and reference in stress fractures, too few cases in current study.
rs12656106	NR3C1, glucocorticoid receptor,	Ref/ref	Yanovich, R., Friedman, E., Milgrom, R., Oberman, B., Freedman, L., & Moran, D. S. (2012). Candidate gene	Function does not seem relevant and reference in

	inflammatory responses		analysis in Israeli soldiers with stress fractures. *Journal of Sports Science & Medicine*, *11*(1), 147.	stress fractures, too few cases in current study.
rs3045	ANKH, controls pyrophosphate levels	Alt	Peach, C. A., Zhang, Y., Dunford, J. E., Brown, M. A., & Carr, A. J. (2007). Cuff tear arthropathy: evidence of functional variation in pyrophosphate metabolism genes. *Clinical Orthopaedics and Related Research (1976-2007)*, *462*, 67-72.	Neither function nor reference directly point to RRI.
rs187483	ANKH, controls pyrophosphate levels	Ref/alt	Peach, C. A., Zhang, Y., Dunford, J. E., Brown, M. A., & Carr, A. J. (2007). Cuff tear arthropathy: evidence of functional variation in pyrophosphate metabolism genes. *Clinical Orthopaedics and Related Research (1976-2007)*, *462*, 67-72.	Neither function nor reference directly point to RRI.

rs4701616	ANKH, controls pyrophosphate levels	Ref	Yanovich, R., Friedman, E., Milgrom, R., Oberman, B., Freedman, L., & Moran, D. S. (2012). Candidate gene analysis in Israeli soldiers with stress fractures. *Journal of Sports Science & Medicine*, *11*(1), 147.	Function does not seem relevant and reference in stress fractures, too few cases in current study.
rs144414988	LSP1P3, pseudogene	Alt	Kim, S. K., Nguyen, C., Jones, K. B., & Tashjian, R. Z. (2021). A genome-wide association study for shoulder impingement and rotator cuff disease. *Journal of shoulder and elbow surgery*, *30*(9), 2134-2145.	Neither function nor reference directly point to RRI.
rs1011814 / rs900379*	FGF10, embryonic development, cell growth, morphogenesis, tissue repair, tumor growth	Alt	Salles, J. I., Amaral, M. V., Aguiar, D. P., Lira, D. A., Quinelato, V., Bonato, L. L., ... & Casado, P. L. (2015). BMP4 and FGF3 haplotypes increase the risk of tendinopathy in volleyball athletes. *Journal of science and medicine in sport*, *18*(2), 150-155.	Function does not seem relevant but reference with lower limb tendinopathy.

			da Rocha Motta, G., Amaral, M. V., Rezende, E., Pitta, R., dos Santos Vieira, T. C., Duarte, M. E. L., ... & Casado, P. L. (2014). Evidence of genetic variations associated with rotator cuff disease. *Journal of shoulder and elbow surgery*, *23*(2), 227-235.	
rs11154027	GJA1, gap junction protein	Ref	Rodas, G., Osaba, L., Arteta, D., Pruna, R., Fernández, D., & Lucia, A. (2019). Genomic prediction of tendinopathy risk in elite team sports. *International Journal of Sports Physiology and Performance*, *15*(4), 489-495.	Function does not seem relevant but reference with lower limb tendinopathy.
rs2234693	ESR1, estrogen receptor 1	Ref	Lian, K., Lui, L., Zmuda, J. M., Nevitt, M. C., Hochberg, M. C., Lee, J. M., ... & Lane, N. E. (2007). Estrogen receptor alpha genotype is associated with a reduced prevalence of radiographic hip osteoarthritis in elderly	Estrogen seems to play a significant role in joint laxity in females, but the reference is not with RRI.

			Caucasian women. *Osteoarthritis and Cartilage*, *15*(8), 972-978.	
rs9340799	ESR1, estrogen receptor 1	Ref/ref	Pontin, P. A., Nogara, P. R. B., Fonseca, F. C. P., Cesar Netto, C., Carvalho, K. C., Soares Junior, J. M., ... & Godoy-Santos, A. (2018). ERα PvuII and XbaI polymorphisms in postmenopausal women with posterior tibial tendon dysfunction: a case control study. *Journal of orthopaedic surgery and research*, *13*, 1-5.	Both function and reference seem to link with potential RRI especially in females.
rs1643821	ESR1, estrogen receptor 1	Ref/ref	Dalewski, B., Kamińska, A., Białkowska, K., Jakubowska, A., & Sobolewska, E. (2020). Association of estrogen receptor 1 and tumor necrosis factor α polymorphisms with temporomandibular joint anterior	Estrogen seems to play a significant role in joint laxity in females, but the reference is not with RRI.

			disc displacement without reduction. *Disease Markers*, *2020*(1), 6351817.	
rs1800629	TNF, tumor necrosis factor	Alt	Furquim, B. D. A., Flamengui, L. M. S. P., Repeke, C. E. P., Cavalla, F., Garlet, G. P., & Conti, P. C. R. (2016). Influence of TNF-α-308 G/A gene polymorphism on temporomandibular disorder. *American Journal of Orthodontics and Dentofacial Orthopedics*, *149*(5), 692-698.	Neither function nor reference directly point to RRI.
rs2010963	VEGFA, vascular endothelial growth factor A	Ref/ref	Hall, E. C., Baumert, P., Larruskain, J., Gil, S. M., Lekue, J. A., Rienzi, E., ... & Erskine, R. M. (2022). The genetic association with injury risk in male academy soccer players depends on maturity status. *Scandinavian Journal of Medicine & Science in Sports*, *32*(2), 338-350.	Function does not seem relevant but reference links to tendon/ligament injuries.

rs10484958	SASH1, scaffold protein involved in the TLR4 signaling pathway	Alt	Tashjian, R. Z., Granger, E. K., Farnham, J. M., Cannon-Albright, L. A., & Teerlink, C. C. (2016). Genome-wide association study for rotator cuff tears identifies two significant single-nucleotide polymorphisms. *Journal of shoulder and elbow surgery*, *25*(2), 174-179.	Neither function nor reference directly point to RRI.
rs970547	COL12A1, alpha chain of type XII collagen	Alt/alt	Bell, R. D., Shultz, S. J., Wideman, L., & Henrich, V. C. (2012). Collagen gene variants previously associated with anterior cruciate ligament injury risk are also associated with joint laxity. *Sports Health*, *4*(4), 312-318.	Both function and reference seem relevant to RRI.
rs4730153	NAMPT, catalyzes the condensation of nicotinamide	Alt/alt	Wang, Y., Meng, F., Wu, J., Long, H., Li, J., Wu, Z., ... & Xie, D. (2022). Associations between adipokines gene polymorphisms and knee osteoarthritis: a meta-analysis. *BMC musculoskeletal disorders*, *23*(1), 166.	Neither function nor reference directly point to RRI.

rs10263021	CNTNAP2, functions in the vertebrate nervous system as cell adhesion molecules and receptors	Alt	Rodas, G., Osaba, L., Arteta, D., Pruna, R., Fernández, D., & Lucia, A. (2019). Genomic prediction of tendinopathy risk in elite team sports. *International Journal of Sports Physiology and Performance*, *15*(4), 489-495.	Function does not seem relevant but reference with tendinopathy.
rs1800797	IL6-AS1, interleukin 6, cytokine that functions in inflammation and the maturation of B cells	Alt	Li, J., Jiang, L., Zhou, X., Wu, L., Li, D., & Chen, G. (2020). The association between Interleukin-6 rs1800795/rs1800797 polymorphisms and risk of rotator cuff tear in a Chinese population. *Bioscience Reports*, *40*(4), BSR20200193.	Inflammatory function is closely linked to overuse injuries but reference is not RRI. **rs1800795

rs1800795	IL6-AS1, interleukin 6, cytokine that functions in inflammation and the maturation of B cells	Ref/ref	Hall, E. C., Baumert, P., Larruskain, J., Gil, S. M., Lekue, J. A., Rienzi, E., ... & Erskine, R. M. (2022). The genetic association with injury risk in male academy soccer players depends on maturity status. *Scandinavian Journal of Medicine & Science in Sports*, *32*(2), 338-350. Li, J., Jiang, L., Zhou, X., Wu, L., Li, D., & Chen, G. (2020). The association between Interleukin-6 rs1800795/rs1800797 polymorphisms and risk of rotator cuff tear in a Chinese population. *Bioscience Reports*, *40*(4), BSR20200193.	Inflammatory function is closely linked to overuse injuries and reference shows link for any (soccer) injuries and muscle injuries
rs1554606	IL6, interleukin 6, cytokine that functions in inflammation and	Ref/ref	Yanovich, R., Friedman, E., Milgrom, R., Oberman, B., Freedman, L., & Moran, D. S. (2012). Candidate gene analysis in Israeli soldiers with stress fractures. *Journal of Sports Science & Medicine*, *11*(1), 147.	Inflammatory function is closely linked to overuse injuries but reference is stress fracture, too few

	the maturation of B cells			cases in current study. **rs1800795
rs2237352 / rs12700903*	CREB5, functions as a CRE-dependent trans-activator	Ref	Yanik, E. L., Keener, J. D., Lin, S. J., Colditz, G. A., Wright, R. W., Evanoff, B. A., ... & Saccone, N. L. (2021). Identification of a novel genetic marker for risk of degenerative rotator cuff disease surgery in the UK biobank. *JBJS*, *103*(14), 1259-1267.	Neither function nor reference directly point to RRI.
rs149047058	COA1, mitochondrial cytochrome c oxidase assembly and mitochondrial respiratory chain	Alt	Kim, S. K., Nguyen, C., Horton, B. H., Avins, A. L., & Abrams, G. D. (2021). Association of COA1 with patellar tendonitis: a genome-wide association analysis. *Med. Sci. Sports Exerc*, *53*, 2419-2424.	Function does not seem relevant but reference with patellar tendonitis.

	complex I assembly			
rs4725069	GLCCI1, may be an early marker for glucocorticoid-induced apoptosis	Alt	Tashjian, R. Z., Kim, S. K., Roche, M. D., Jones, K. B., & Teerlink, C. C. (2021). Genetic variants associated with rotator cuff tearing utilizing multiple population-based genetic resources. *Journal of Shoulder and Elbow Surgery*, *30*(3), 520-531.	Neither function nor reference directly point to RRI.
rs12154667	CALCR, maintaining calcium homeostasis and regulating osteoclast-	Ref/ref	Yanovich, R., Friedman, E., Milgrom, R., Oberman, B., Freedman, L., & Moran, D. S. (2012). Candidate gene analysis in Israeli soldiers with stress fractures. *Journal of Sports Science & Medicine*, *11*(1), 147.	Both function and reference relevant to stress fracture, too few cases in current study.

	mediated bone resorption			
rs1548456	CALCR, maintaining calcium homeostasis and regulating osteoclast-mediated bone resorption	Ref/ref	Yanovich, R., Friedman, E., Milgrom, R., Oberman, B., Freedman, L., & Moran, D. S. (2012). Candidate gene analysis in Israeli soldiers with stress fractures. *Journal of Sports Science & Medicine*, *11*(1), 147.	Both function and reference relevant to stress fracture, too few cases in current study.
rs420257	COL1A2, pro-alpha2 chain of type I collagen	Ref/ref	Yanovich, R., Friedman, E., Milgrom, R., Oberman, B., Freedman, L., & Moran, D. S. (2012). Candidate gene analysis in Israeli soldiers with stress fractures. *Journal of Sports Science & Medicine*, *11*(1), 147.	Function seems relevant but reference is with stress fracture, too few cases in current study.

rs42517	COL1A2, pro-alpha2 chain of type I collagen	Alt/alt	Yanovich, R., Friedman, E., Milgrom, R., Oberman, B., Freedman, L., & Moran, D. S. (2012). Candidate gene analysis in Israeli soldiers with stress fractures. *Journal of Sports Science & Medicine*, *11*(1), 147.	Function seems relevant but reference is with stress fracture, too few cases in current study.
rs42522	COL1A2, pro-alpha2 chain of type I collagen	Ref/ref	Yanovich, R., Friedman, E., Milgrom, R., Oberman, B., Freedman, L., & Moran, D. S. (2012). Candidate gene analysis in Israeli soldiers with stress fractures. *Journal of Sports Science & Medicine*, *11*(1), 147.	Function seems relevant but reference is with stress fracture, too few cases in current study.
rs3216902	COL1A2, pro-alpha2 chain of type I collagen	Ref	Korvala, J., Hartikka, H., Pihlajamäki, H., Solovieva, S., Ruohola, J. P., Sahi, T., ... & Männikkö, M. (2010). Genetic predisposition for femoral neck stress fractures in military conscripts. *BMC genetics*, *11*, 1-9.	Function seems relevant but reference is with stress fracture, too few cases in current study. **rs149047058

rs42531	COL1A2, pro-alpha2 chain of type I collagen	Ref/ref, Alt/alt	Yanovich, R., Friedman, E., Milgrom, R., Oberman, B., Freedman, L., & Moran, D. S. (2012). Candidate gene analysis in Israeli soldiers with stress fractures. *Journal of Sports Science & Medicine*, *11*(1), 147.	Function seems relevant but reference is with stress fracture, too few cases in current study.
rs413826	COL1A2, pro-alpha2 chain of type I collagen	Ref/ref	Yanovich, R., Friedman, E., Milgrom, R., Oberman, B., Freedman, L., & Moran, D. S. (2012). Candidate gene analysis in Israeli soldiers with stress fractures. *Journal of Sports Science & Medicine*, *11*(1), 147.	Function seems relevant but reference is with stress fracture, too few cases in current study.
rs35360670	MTSS1, cellular response to fluid shear stress	Alt	Kim, S. K., Ahmed, M. A., Avins, A. L., & Ioannidis, J. P. (2017). A genetic marker associated with De Quervain's tenosynovitis. *International journal of sports medicine*, *38*(12), 942-948.	Neither function nor reference directly point to RRI.

rs13317	FGFR1, binds both acidic and basic fibroblast growth factors and is involved in limb induction	Ref/ref	da Rocha Motta, G., Amaral, M. V., Rezende, E., Pitta, R., dos Santos Vieira, T. C., Duarte, M. E. L., ... & Casado, P. L. (2014). Evidence of genetic variations associated with rotator cuff disease. *Journal of shoulder and elbow surgery*, *23*(2), 227-235.	Neither function nor reference directly point to RRI.
rs1800972	DEFB1, antimicrobial peptide implicated in the resistance of epithelial surfaces to microbial colonization	Alt/alt	da Rocha Motta, G., Amaral, M. V., Rezende, E., Pitta, R., dos Santos Vieira, T. C., Duarte, M. E. L., ... & Casado, P. L. (2014). Evidence of genetic variations associated with rotator cuff disease. *Journal of shoulder and elbow surgery*, *23*(2), 227-235.	Neither function nor reference directly point to RRI.

rs7035322	TNC, guidance of migrating neurons as well as axons during development, synaptic plasticity, and neuronal regeneration	Ref	Kluger, R., Burgstaller, J., Vogl, C., Brem, G., Skultety, M., & Mueller, S. (2017). Candidate gene approach identifies six SNPs in tenascin-C (TNC) associated with degenerative rotator cuff tears. *Journal of Orthopaedic Research*, *35*(4), 894-901.	Neither function nor reference directly point to RRI.
rs7021589	TNC, guidance of migrating neurons as well as axons during development, synaptic plasticity,	Alt	Tashjian, R. Z., Kim, S. K., Roche, M. D., Jones, K. B., & Teerlink, C. C. (2021). Genetic variants associated with rotator cuff tearing utilizing multiple population-based genetic resources. *Journal of Shoulder and Elbow Surgery*, *30*(3), 520-531.	Neither function nor reference directly point to RRI.

	and neuronal regeneration		Kluger, R., Burgstaller, J., Vogl, C., Brem, G., Skultety, M., & Mueller, S. (2017). Candidate gene approach identifies six SNPs in tenascin-C (TNC) associated with degenerative rotator cuff tears. *Journal of Orthopaedic Research*, *35*(4), 894-901.	
rs72758637	TNC, guidance of migrating neurons as well as axons during development, synaptic plasticity, and neuronal regeneration	Alt	Tashjian, R. Z., Kim, S. K., Roche, M. D., Jones, K. B., & Teerlink, C. C. (2021). Genetic variants associated with rotator cuff tearing utilizing multiple population-based genetic resources. *Journal of Shoulder and Elbow Surgery*, *30*(3), 520-531.<br>Kluger, R., Burgstaller, J., Vogl, C., Brem, G., Skultety, M., & Mueller, S. (2017). Candidate gene approach identifies six SNPs in tenascin-C (TNC) associated with	Neither function nor reference directly point to RRI.

			degenerative rotator cuff tears. *Journal of Orthopaedic Research*, *35*(4), 894-901.	
rs10759753	TNC, guidance of migrating neurons as well as axons during development, synaptic plasticity, and neuronal regeneration	Ref	Kluger, R., Burgstaller, J., Vogl, C., Brem, G., Skultety, M., & Mueller, S. (2017). Candidate gene approach identifies six SNPs in tenascin-C (TNC) associated with degenerative rotator cuff tears. *Journal of Orthopaedic Research*, *35*(4), 894-901.	Neither function nor reference directly point to RRI.
rs2104772	TNC, guidance of migrating neurons as well as axons during	Alt	Saunders, C. J., van der Merwe, L., Posthumus, M., Cook, J., Handley, C. J., Collins, M., & September, A. V. (2013). Investigation of variants within the COL27A1 and TNC genes and Achilles tendinopathy in two	Function does not seem relevant but reference shows link with Achilles tendinopathy.

	development, synaptic plasticity, and neuronal regeneration		populations. *Journal of Orthopaedic Research*, *31*(4), 632-637.	
rs1330363	TNC, guidance of migrating neurons as well as axons during development, synaptic plasticity, and neuronal regeneration	Ref	Saunders, C. J., van der Merwe, L., Posthumus, M., Cook, J., Handley, C. J., Collins, M., & September, A. V. (2013). Investigation of variants within the COL27A1 and TNC genes and Achilles tendinopathy in two populations. *Journal of Orthopaedic Research*, *31*(4), 632-637.	Function does not seem relevant but reference shows link with Achilles tendinopathy.

rs3789870	TNC, guidance of migrating neurons as well as axons during development, synaptic plasticity, and neuronal regeneration	Ref	Kluger, R., Burgstaller, J., Vogl, C., Brem, G., Skultety, M., & Mueller, S. (2017). Candidate gene approach identifies six SNPs in tenascin-C (TNC) associated with degenerative rotator cuff tears. *Journal of Orthopaedic Research*, *35*(4), 894-901.	Neither function nor reference directly point to RRI.
rs1138545	TNC, guidance of migrating neurons as well as axons during development, synaptic plasticity,	Alt	Tashjian, R. Z., Kim, S. K., Roche, M. D., Jones, K. B., & Teerlink, C. C. (2021). Genetic variants associated with rotator cuff tearing utilizing multiple population-based genetic resources. *Journal of Shoulder and Elbow Surgery*, *30*(3), 520-531.	Neither function nor reference directly point to RRI.

	and neuronal regeneration		Kluger, R., Burgstaller, J., Vogl, C., Brem, G., Skultety, M., & Mueller, S. (2017). Candidate gene approach identifies six SNPs in tenascin-C (TNC) associated with degenerative rotator cuff tears. *Journal of Orthopaedic Research*, *35*(4), 894-901.	
rs13946	COL5A1, collagen type V alpha 1 chain	Alt	Guo, R., Ji, Z., Gao, S., Aizezi, A., Fan, Y., Wang, Z., & Ning, K. (2022). Association of COL5A1 gene polymorphisms and musculoskeletal soft tissue injuries: a meta-analysis based on 21 observational studies. *Journal of Orthopaedic Surgery and Research*, *17*(1), 129.<br>Altinisik, J., Meric, G., Erduran, M., Ates, O., Ulusal, A. E., & Akseki, D. (2015). The BstUI and DpnII variants of	Both function and reference seem relevant to RRI.

			the COL5A1 gene are associated with tennis elbow. *The American journal of sports medicine*, *43*(7), 1784-1789.	
rs12722	COL5A1, collagen type V alpha 1 chain	Alt	Lv, Z. T., Gao, S. T., Cheng, P., Liang, S., Yu, S. Y., Yang, Q., & Chen, A. M. (2018). Association between polymorphism rs12722 in COL5A1 and musculoskeletal soft tissue injuries: a systematic review and meta-analysis. *Oncotarget*, *9*(20), 15365. September, A. V., Cook, J., Handley, C. J., van der Merwe, L., Schwellnus, M. P., & Collins, M. (2009). Variants within the COL5A1 gene are associated with Achilles tendinopathy in two populations. *British journal of sports medicine*, *43*(5), 357-365. Hall, E. C., Baumert, P., Larruskain, J., Gil, S. M., Lekue, J. A., Rienzi, E., ... & Erskine, R. M. (2022). The genetic	Both function and reference seem relevant to RRI.

			association with injury risk in male academy soccer players depends on maturity status. *Scandinavian Journal of Medicine & Science in Sports*, *32*(2), 338-350.<br>Guo, R., Ji, Z., Gao, S., Aizezi, A., Fan, Y., Wang, Z., & Ning, K. (2022). Association of COL5A1 gene polymorphisms and musculoskeletal soft tissue injuries: a meta-analysis based on 21 observational studies. *Journal of Orthopaedic Surgery and Research*, *17*(1), 129.<br>Dalewski, B., Białkowska, K., Pałka, Ł., Jakubowska, A., Kiczmer, P., & Sobolewska, E. (2021). COL5A1 RS12722 is associated with temporomandibular joint anterior disc displacement without reduction in polish caucasians. *Cells*, *10*(9), 2423.	

			Bell, R. D., Shultz, S. J., Wideman, L., & Henrich, V. C. (2012). Collagen gene variants previously associated with anterior cruciate ligament injury risk are also associated with joint laxity. *Sports Health*, *4*(4), 312-318.<br>Altinisik, J., Meric, G., Erduran, M., Ates, O., Ulusal, A. E., & Akseki, D. (2015). The BstUI and DpnII variants of the COL5A1 gene are associated with tennis elbow. *The American journal of sports medicine*, *43*(7), 1784-1789.	
rs3196378	COL5A1, collagen type V alpha 1 chain	Alt	September, A. V., Cook, J., Handley, C. J., van der Merwe, L., Schwellnus, M. P., & Collins, M. (2009). Variants within the COL5A1 gene are associated with Achilles tendinopathy in two populations. *British journal of sports medicine*, *43*(5), 357-365.	Both function and reference seem relevant to RRI. **rs12722

			Guo, R., Ji, Z., Gao, S., Aizezi, A., Fan, Y., Wang, Z., & Ning, K. (2022). Association of COL5A1 gene polymorphisms and musculoskeletal soft tissue injuries: a meta-analysis based on 21 observational studies. *Journal of Orthopaedic Surgery and Research*, *17*(1), 129.<br>Figueiredo, E. A., Loyola, L. C., Belangero, P. S., Campos Ribeiro-dos-Santos, Â. K., Emanuel Batista Santos, S., Cohen, C., ... & Leal, M. F. (2020). Rotator cuff tear susceptibility is associated with variants in genes involved in tendon extracellular matrix homeostasis. *Journal of Orthopaedic Research®*, *38*(1), 192-201.	

rs1134170	COL5A1, collagen type V alpha 1 chain	Alt/alt	Abrahams, Y., Laguette, M. J., Prince, S., & Collins, M. (2013). Polymorphisms within the COL5A1 3'-UTR that alters mRNA structure and the MIR608 gene are associated with Achilles tendinopathy. *Annals of human genetics*, *77*(3), 204-214.	Both function and reference seem relevant to RRI. **rs13946
rs10992075	ROR2, early formation of the chondrocytes	Ref/alt	Yanovich, R., Friedman, E., Milgrom, R., Oberman, B., Freedman, L., & Moran, D. S. (2012). Candidate gene analysis in Israeli soldiers with stress fractures. *Journal of Sports Science & Medicine*, *11*(1), 147.	Function does not seem relevant and reference is with stress fracture, too few cases in current study.
rs1590	TGFBR1, serine/threonine protein kinase	Alt	Figueiredo, E. A., Loyola, L. C., Belangero, P. S., Campos Ribeiro-dos-Santos, Â. K., Emanuel Batista Santos, S., Cohen, C., ... & Leal, M. F. (2020). Rotator cuff tear susceptibility is associated with variants in genes involved in tendon extracellular matrix	Neither function nor reference directly point to RRI.

			homeostasis. *Journal of Orthopaedic Research®*, *38*(1), 192-201.	
rs144371252	FRMPD4, positive regulator of dendritic spine morphogenesis and density	Alt	Kim, S. K., Nguyen, C., Jones, K. B., & Tashjian, R. Z. (2021). A genome-wide association study for shoulder impingement and rotator cuff disease. *Journal of shoulder and elbow surgery*, *30*(9), 2134-2145.	Neither function nor reference directly point to RRI.
rs761804508	ACOT9, mitochondrial acyl-CoA thioesterase of unknown function	Alt	Kim, S. K., Nguyen, C., Jones, K. B., & Tashjian, R. Z. (2021). A genome-wide association study for shoulder impingement and rotator cuff disease. *Journal of shoulder and elbow surgery*, *30*(9), 2134-2145.	Neither function nor reference directly point to RRI.
Class1_SNP_risk_score	Combined risk score of all class 1			

	SNPs based on risky alleles			
Class12_SNP_risk_score	Combined risk score of all class 1 and 2 SNPs based on risky alleles			
Class123_SNP_risk_score	Combined risk score of all SNPs based on risky alleles			

*These SNPs have identical genotype distributions among the study population.

**This SNP is classified as class 3 because it has >90% identical genotype information within the study population compared to another SNP, while the other SNP is considered more relevant to RRI.

[1] Separate questions were asked for number of days injured at different body regions during the past 12 months. For each region, answers >365 were assumed to be 365. Lower limb days total was the added value from all lower body regions consisting of knee, ankle/Achilles, calf/shin, foot/toes, hip/groin, and thigh (total of 6 regions).

[2] Scoring was based on the World Athletics Scoring Table 2022 version (Technical Information | Official Documents). Within the baseline questionnaire, participants were asked for their best performance during the past 6 months for 5km track, 5km road, 10km track, 10km road, half marathon, and marathon separately. For those who answered more than one questions, the highest score was chosen. For those who did not have recorded performance for any of the above (some competed at other distances, some recorded performance outside of the 6-month time frame), search was conducted on thePowerof10 website (Power of 10) for their closest scorable performance.

[3] For all male participants and for female participants <18 or >39 years of age at the date of questionnaire completion, LEAF-Q score was assumed to be 0. This is in accordance with the original LEAF-Q guidance (The LEAF questionnaire: a screening tool for the identification of female athletes at risk for the female athlete triad | British Journal of Sports Medicine).

[4] VILR and VALR were only calculated for participants whose rearfoot strikes consisted more than 70% of the total number of strikes during the running trial (Differences in kinetic variables between injured and noninjured novice runners: A prospective cohort study - ScienceDirect). Rearfoot strike was defined as any strike that presented with >1 peaks after vertical GRF were passed through

a lowpass filter. A peak was defined as a point where the frame both immediately before and after it had lower vertical GRFs. A more common approach to define rearfoot runners is through visual inspection during kinematic analysis. However, since this study did not include kinematic analysis, such an approach was not viable. The region of interest was defined as the duration between landing initiation and the first point during a foot strike where GRF > 75% BW and slope < 15 * BW/s; VILR was defined as the highest slope between 20%-100% of the region of interest; VALR was defined as the average slope between 20%-80% of the region of interest (Impact-Related Ground Reaction Forces Are More Strongly Associated With Some Running Injuries Than Others - Caleb D. Johnson, Adam S. Tenforde, Jereme Outerleys, Julia Reilly, Irene S. Davis, 2020). VALR and VILR were only calculated for rearfoot strikes, meaning that forefoot strikes from rearfoot strikers were not average in during calculation. For forefoot strikers, VILR and VALR were assumed to be 0.

[5] A step was defined as a landing phase and an ensuing flight phase. A landing phase was defined as a period of time where vertical GRF rises from 0 to above bodyweight and then returns to 0, and a flight phase is the period of time after each landing phase and before the initiation of the next landing phase.

[6] An alt_strike participant was defined as anyone whose number of rearfoot strikes was between 40%-60% of their total number of strikes. It was observed during data analysis that these participants consistently presented with 2 peaks and 1 peak during alternating

strikes, meaning that they were landing with rearfoot using one leg while landing with forefoot/midfoot using the other leg. This may indicate some form of asymmetry so was included as an additional class 3 feature.

[7] Each food diary consisted of 3 days of food log, and no specification was given on how close the 3 days should be as long as they fall within the 4-month period between the participant's 2 visits. It was recommended that the participants do 2*weekdays and 1*weekend day for each log since many people eat differently during weekdays vs. weekends. For each nutrient, the value was averaged among the 3 days within each log. The 3 dates within each food log were averaged and mapped against the Sunday of the average date's week, and the average nutrient value was placed on that week's weekly questionnaire. If the participant only completed 1 round of 3-day food diary, their nutrient value would be the averaged value for every week. If the participant completed more than 1 round of food diaries, all weeks before the first placed average nutrient value took the value of the first average nutrient value; all weeks after the last placed average nutrient value took the value of the last average nutrient value; all weeks in between 2 average nutrient values were placed on a linear slope between the previous and the next placed nutrient values (X axis time, Y axis value), and were interpolated based on the linear equation. This was to prevent sudden dramatic changes in nutritional intake values between 2 weeks.

[8] Caloric expenditure was calculated separately for every week based on the participants' answers in each weekly questionnaire. Different running speeds, strength and conditioning exercises, and non-running exercises were mapped against the Compendium of

Physical Activities (Compendium of Physical Activities – Quantifying Physical Activity Energy Expenditure) for their respective METs. Participants' masses during the corresponding week and weekly MET were used to calculate total energy expenditure, which was then divided by 7 to get a daily average energy expenditure value for each week.

[9] All measurements during bone scan sessions except for height were linearly interpolated similar to nutrient values (mentioned above). Each scanning session was mapped against the Sunday during that week, and the scan/mass measurement results were placed on the weekly questionnaire during that week. All weeks prior to the first scanning session (typically none since weekly questionnaires start after the first visit) and after the last scanning session took values of the initial and the last session, respectively. Weeks between 2 sessions were mapped onto a slope drawn between the previous and the next sessions' values (X axis time, Y axis value). This was to prevent sudden dramatic change in values between 2 weeks.

[10] Thigh and lower leg regions were custom-drawn for each scan. The top point for the thigh region was defined as the horizontal extension from the anterior-superior iliac spine. Another point on the side was defined close to the horizontal extension of the greater trochanter so that the region can fully contain all thigh tissue. A middle point was defined at the tip of the coccyx, and if the tip of the coccyx was not visible, a visual approximation was used. The bottom of the thigh region/top of the lower leg region was defined as a horizontal line drawn at the knee joint space. Vertical lines were then extended to the bottom of the scan region to define the lower leg

regions. The best effort was made so that the vertical lines in the middle could separate tissues from the two legs. An example of custom-defined regions is shown below:

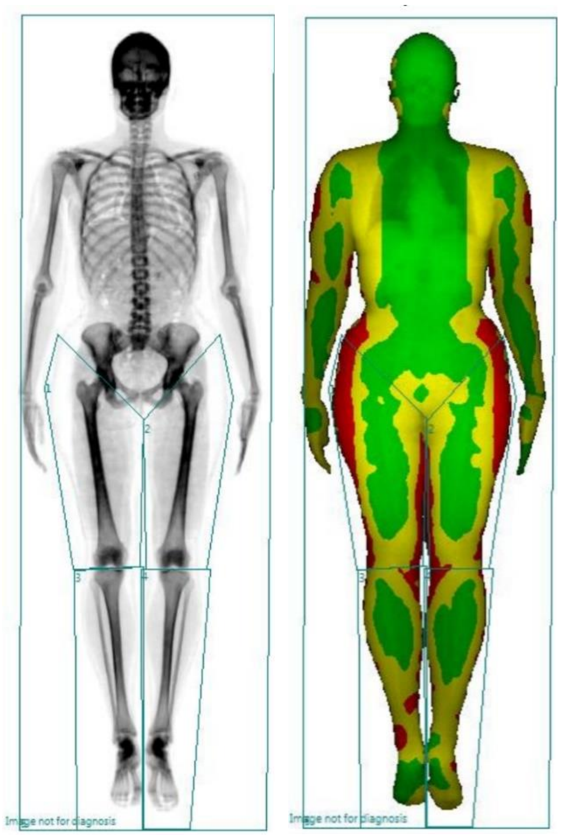

Regions 1, 2, 3, and 4 are right thigh, left thigh, right lower leg, and left lower leg, respectively.

[11] When the risky genotype is ‘ref/alt’, meaning it is heterozygotic, then genotype ‘ref/alt’ is given a risk score of 1, and ‘ref/ref’ and ‘alt/alt’ are both given a risk score of 0. Same goes the other way around (if the risky genotype is ‘ref/ref, alt/alt’, meaning both ‘ref/ref’ and ‘alt/alt’ increase risk, while ‘ref/alt’ reduces risk).

[12] When the risky genotype is ‘alt’, meaning the alternative allele increases the risk of injury, genotype ‘alt/alt’ receives a risk score of 1, genotype ‘ref/alt’ receives a risk score of 0.5, and genotype ‘ref/ref’ receives a risk score of 0. Same goes the other way around (if the risky genotype is ‘ref’).

[13] When the risky genotype is ‘ref/ref’, meaning the reference allele is recessive and increases the risk of injury, genotype ‘ref/ref’ receives a risk score of 1, while genotype ‘ref/alt’ and ‘alt/alt’ both receive a risk score of 0. Same goes the other way around (if the risky genotype is ‘alt/alt’).

Supplementary Material Section 5: Model Output

Jump to: hyperparameter tuning results for class 1 dataset hyperparameter tuning results for "all features" dataset selected feature names for class 1 dataset selected feature names for "all features" dataset

Feature Selection Results Comparison

Feature indexes refer to their column index number (using 0-indexing which is the default in python) in the original dataset (class1_dataset.xlsx and class123_dataset.xlsx).

Supplementary Table 15: Class 1 feature selection

	LASSO-AUC	Relief-AUC	LASSO-features	LASSO-alpha	Relief-features
decision tree	0.584473348	0.729555428	24, 20, 16, 38, 15, 12, 3, 10, 13, 21, 9, 22, 19, 26, 28, 33, 2	0.006951928	37, 30, 25, 26, 29, 28, 32, 38, 36, 10
random forest	0.767295761	0.765169092	24, 20, 16, 15, 12, 3, 10, 13, 21, 9, 22, 28, 29, 19, 38, 2, 6, 26, 31, 5, 33, 11, 35, 34, 1, 8, 36, 32, 30, 23, 14, 0, 4, 27, 37, 18, 17	0.037926902	37, 30, 25, 26, 29, 28, 32, 38, 36, 10, 7, 13, 33, 12, 34, 24, 14, 18, 3, 15, 6, 16, 8, 11, 17, 4, 1, 9, 19, 31, 5, 0, 20, 35, 22, 23, 2, 27, 21
SVM	0.708094201	0.70662508	24, 20, 16, 38, 15, 12, 3, 10, 13, 21, 9, 22, 19, 26, 28, 33, 2, 29, 6, 5, 35, 32, 30, 11, 31, 25, 8, 1, 23, 37, 27, 14, 0, 4, 18, 17, 36	0.006951928	37, 30, 25, 26, 29, 28, 32, 38, 36, 10, 7, 13, 33, 12, 34, 24, 14, 18, 3, 15, 6, 16, 8, 11, 17, 4, 1, 9, 19, 31, 5, 0, 20, 35, 22, 23, 2
KNN	0.69234721	0.694794175	24, 20, 16, 15, 12, 3, 10, 13, 21, 9, 22, 28, 29, 19, 38, 2, 6, 26, 31, 5, 33, 11, 35, 34, 1, 8, 36, 32, 30, 23, 14, 0, 4, 27, 37, 18, 17	0.037926902	37, 30, 25, 26, 29, 28, 32, 38, 36, 10, 7, 13, 33, 12, 34, 24, 14, 18, 3, 15, 6, 16, 8, 11, 17, 4, 1, 9, 19, 31, 5, 0, 20, 35, 22, 23

naïve bayes	0.662029907	0.66105154	24	0.1	37, 30, 25, 26, 29, 28, 32, 38, 36, 10, 7, 13, 33, 12, 34, 24, 14, 18, 3, 15, 6
adaboost	0.695538385	0.694519778	24, 20, 16, 15, 12, 3, 10, 13, 21, 9, 22, 28, 29, 19, 38, 2, 6, 26, 31	0.037926902	37, 30, 25, 26, 29, 28, 32, 38, 36, 10, 7, 13, 33, 12, 34, 24, 14, 18, 3, 15, 6, 16, 8, 11
gradient boosting	0.714393733	0.720638444	24, 20, 16, 15, 12, 3, 10, 13, 21, 9, 22, 28, 29, 19, 38, 2, 6, 26, 31, 5, 33, 11, 35, 34, 1, 8, 36	0.037926902	37, 30, 25, 26, 29, 28, 32, 38, 36, 10, 7, 13, 33, 12
MLP	0.734657131	0.733912176	24, 20, 16, 15, 12, 3, 10, 13, 21, 22, 9, 28, 29, 19, 31, 2, 6, 5, 26, 34, 33, 36, 11, 1, 27, 37, 8, 25, 14, 23, 0, 35, 4, 32, 30, 18	0.078475997	37, 30, 25, 26, 29, 28, 32, 38, 36, 10, 7, 13, 33, 12, 34, 24, 14, 18, 3, 15, 6, 16, 8, 11, 17, 4, 1, 9, 19, 31, 5, 0, 20, 35, 22, 23
logistic regression	0.671088876	0.6604	24, 38, 20, 16, 15, 12, 3, 10, 13, 21, 9, 22, 26, 33, 19, 2, 35, 32, 28, 6, 30	0.011288379	37, 30, 25, 26, 29, 28, 32, 38, 36, 10, 7, 13, 33, 12, 34, 24, 14, 18, 3, 15, 6, 16, 8, 11, 17, 4, 1, 9, 19, 31, 5, 0, 20, 35, 22, 23, 2, 27, 21
Baysian network	0.6494	0.6494	24, 30, 28, 9, 3, 12, 13, 8, 2, 10, 7, 6, 27, 25, 36, 20, 33, 19, 15	0.00001	29, 15, 10, 5, 11, 36, 12, 3, 38, 33, 2, 16, 14, 13, 4, 7, 6, 24

Supplementary Table 16: Class 1-3 feature selection

	LASSO-AUC	Relief-AUC	LASSO-features	LASSO-alpha	Relief-features
decision tree	0.601006395	0.7276	138, 244, 224, 137, 118, 103, 204, 230, 3, 86, 42, 24, 13, 21, 78, 33, 88, 183, 123, 119, 126, 210, 122, 65, 201, 5, 253, 30, 109, 227, 90, 49, 214, 52, 80, 25, 247, 217, 22, 229, 157, 115, 68, 155, 206, 182, 173, 59, 142, 44, 26, 28, 231, 232, 82, 50, 156, 116, 243, 1, 202, 61, 251, 174, 58, 240, 154, 74, 4, 145, 55, 170, 57, 255, 11, 199, 172, 133, 62, 29, 32, 81, 9, 222, 213, 67, 73, 40, 190, 203, 205, 223, 168, 165, 221, 0, 69, 184, 71, 148, 20, 189, 121, 151, 64,	0.1	28, 234, 36, 215, 189, 37, 77, 26, 250, 188, 236, 181, 70, 55, 48, 67, 202, 244, 78, 65, 52, 237, 211, 57, 185, 229, 231, 219, 62, 220, 51, 200, 30, 242, 233, 198, 34, 25, 212, 205, 252, 32, 29, 248, 253, 75, 66, 247, 56, 58, 12, 221, 41, 197, 7, 63, 217, 1, 251, 199

			92, 171, 167, 197, 185, 147, 188, 187, 226, 177, 176, 180, 196, 51, 124, 56, 117, 215, 27, 105, 18, 72, 163, 200, 31, 38, 238, 35, 160, 95, 12, 146, 166, 128, 41, 70, 77, 225, 175, 113, 234, 130, 120, 39		
random forest	0.767986759	0.7733	138, 244, 137, 224, 118, 103, 230, 42, 78, 204, 13, 33, 86, 123, 24, 3, 5, 21, 183, 126, 119, 65, 201, 30, 253, 122, 88, 49, 210, 80, 214, 217, 52, 206, 173, 90, 44, 59, 71, 229, 25, 157, 68, 247, 227, 182, 28	0.061584821	28, 234, 36, 215, 189, 37, 77, 26, 250, 188, 236, 181, 70, 55, 48, 67, 202, 244, 78, 65, 52, 237, 211, 57, 185, 229, 231, 219, 62, 220, 51, 200, 30, 242, 233, 198, 34, 25, 212, 205, 252, 32, 29, 248, 253, 75, 66, 247, 56, 58, 12, 221, 41, 197, 7, 63, 217, 1, 251, 199, 114, 49, 24, 50, 101, 80, 186, 232, 207, 130, 79, 115, 43, 243, 190, 137, 136, 108, 46, 214, 17, 10, 110, 88, 125, 182, 33, 203, 225, 68, 213, 227, 126, 93, 81, 14, 13, 104, 92, 42, 201, 129, 177, 122, 134, 133, 60, 19, 31, 9, 135, 98, 8, 45, 3, 47, 4, 6, 226, 116, 106, 90, 15, 105, 138, 18, 89, 84, 100, 44, 228, 131, 38, 112, 103, 96, 16, 127, 206, 117, 11, 102, 176, 128, 97, 0, 132, 5, 256, 61, 235, 87, 91, 193, 39, 111, 64, 180, 99, 95, 179, 35, 191, 246, 94, 238, 109, 22, 249, 187, 204, 245, 174, 53, 40, 86, 107, 119, 222, 239, 183, 157, 141, 123, 20, 196, 85, 83, 82, 167, 23, 139, 241, 72, 159, 2, 192, 175, 223, 54, 156, 73, 69, 208, 161, 120, 195, 158
SVM	0.716759342	0.7074	138, 244, 224, 137, 118, 103, 204, 230, 3, 86, 42, 24, 13, 21, 78, 33, 88, 183, 123, 119, 126, 210, 122, 65, 201, 5, 253, 30, 109, 227, 90, 49, 214, 52, 80, 25, 247, 217, 22, 229, 157, 115	0.1	28, 234, 36, 215, 189, 37, 77, 26, 250, 188, 236, 181, 70, 55, 48, 67, 202, 244, 78, 65, 52, 237, 211, 57, 185, 229, 231, 219, 62, 220, 51, 200, 30, 242, 233, 198, 34, 25, 212, 205, 252, 32, 29, 248, 253, 75, 66, 247, 56, 58, 12, 221, 41, 197, 7, 63, 217, 1, 251, 199, 114, 49, 24, 50, 101, 80, 186, 232, 207, 130, 79, 115, 43,

					243, 190, 137, 136, 108, 46, 214, 17, 10, 110, 88, 125, 182, 33, 203, 225, 68, 213, 227, 126, 93, 81, 14, 13, 104, 92, 42, 201, 129, 177, 122, 134, 133, 60, 19, 31, 9, 135, 98, 8, 45, 3, 47, 4, 6, 226, 116, 106, 90, 15, 105, 138, 18, 89, 84, 100, 44, 228, 131, 38, 112, 103, 96, 16, 127, 206, 117, 11, 102, 176, 128, 97, 0, 132, 5, 256, 61, 235, 87, 91, 193, 39, 111, 64, 180, 99, 95, 179, 35, 191, 246, 94, 238, 109, 22, 249, 187, 204, 245, 174, 53, 40, 86, 107, 119, 222, 239, 183, 157, 141, 123, 20, 196, 85, 83, 82, 167, 23, 139, 241, 72, 159, 2, 192, 175, 223
KNN	0.700922586	0.7011	138, 244, 224, 137, 118, 103, 230, 204, 42	0.078475997	28, 234, 36, 215, 189, 37, 77, 26, 250, 188, 236, 181, 70, 55, 48, 67, 202, 244, 78, 65, 52, 237, 211, 57, 185, 229, 231, 219, 62, 220, 51, 200, 30, 242, 233, 198, 34, 25, 212, 205, 252, 32, 29, 248, 253, 75, 66, 247, 56, 58, 12, 221, 41, 197, 7, 63, 217, 1, 251, 199, 114, 49, 24, 50, 101, 80, 186, 232, 207, 130, 79, 115, 43, 243, 190, 137, 136, 108, 46, 214, 17, 10, 110, 88, 125, 182, 33, 203, 225, 68, 213, 227, 126, 93, 81, 14, 13, 104, 92, 42, 201, 129, 177, 122, 134, 133, 60, 19, 31, 9, 135, 98, 8, 45, 3, 47, 4, 6, 226, 116, 106, 90, 15
naïve bayes	0.682788866	0.6949	137, 118, 244, 224, 78, 138, 5, 154, 13, 86, 140, 183, 103, 33, 126, 230, 42, 71, 201, 65, 49, 55, 240, 253, 217, 206, 204, 74, 30, 59, 4, 123, 102, 119, 187, 28, 82, 73, 24, 52, 214, 199, 203, 202, 84, 190, 3, 0, 44, 61, 251, 182, 255, 21, 9, 189, 50, 80, 108, 128, 134, 11, 58, 232, 184, 212, 173, 194, 226, 213, 122, 209, 210, 68, 125, 243, 109,	0.005455595	28, 234, 36, 215, 189, 37, 77, 26, 250, 188, 236, 181, 70, 55, 48, 67, 202, 244, 78, 65, 52, 237, 211, 57, 185, 229, 231, 219, 62, 220, 51, 200, 30, 242, 233, 198, 34, 25, 212, 205, 252, 32, 29, 248, 253, 75, 66, 247, 56, 58, 12, 221, 41, 197, 7, 63, 217, 1, 251, 199, 114, 49, 24, 50, 101, 80, 186, 232, 207, 130, 79, 115, 43, 243, 190, 137, 136, 108, 46, 214, 17, 10, 110,

			142, 41, 85, 1, 145, 67, 7, 100, 157, 170, 38, 238, 40, 220, 25, 60, 121, 229, 193, 22, 155, 116, 191, 215, 177, 223, 114, 247, 156, 26, 168, 18, 88, 196, 179, 115, 29, 90, 227, 19, 180, 133, 231, 172, 72, 96, 87, 135, 171, 132, 17, 165, 56, 45, 62, 127, 221, 31, 53, 35, 32, 219, 48, 174, 57, 130, 104, 245, 97, 148, 228, 185, 151, 99, 34, 188, 160, 167, 66, 254, 163, 234, 10, 195, 81, 14, 222, 20, 98, 169, 198, 77, 200, 12, 249, 225, 51, 15, 69, 162, 207, 131, 205, 147, 70, 2, 208, 113, 101, 186, 47, 76, 176, 161, 39, 124, 211, 256, 252, 166, 75, 250, 248, 91, 237, 54, 233, 117, 43, 112, 216		88, 125, 182, 33, 203, 225, 68, 213, 227, 126, 93, 81, 14, 13, 104, 92, 42, 201, 129, 177, 122, 134, 133, 60, 19, 31, 9, 135, 98, 8, 45, 3, 47, 4, 6, 226, 116, 106, 90, 15, 105, 138, 18, 89, 84, 100, 44, 228, 131, 38, 112, 103, 96, 16, 127, 206, 117, 11, 102, 176, 128, 97, 0, 132, 5, 256, 61, 235, 87, 91, 193, 39
adaboost	0.714989998	0.7089	137, 118, 244, 224, 5, 78, 154, 13, 138, 140, 86, 183, 103, 33, 126, 230, 42, 201, 71, 65, 49, 55, 240, 74, 206, 217, 253, 59, 102, 30, 4, 204, 187, 28, 82, 84, 73, 119, 123, 52, 199, 214, 203, 190, 202	0.001274275	28, 234, 36, 215, 189, 37, 77, 26, 250, 188, 236, 181, 70, 55, 48, 67, 202, 244, 78, 65, 52, 237, 211, 57, 185, 229, 231, 219, 62, 220, 51, 200, 30, 242, 233, 198, 34, 25, 212, 205, 252, 32, 29, 248, 253, 75, 66, 247, 56, 58, 12, 221, 41, 197, 7, 63, 217, 1, 251, 199, 114, 49, 24, 50, 101, 80, 186, 232, 207, 130, 79, 115, 43, 243, 190, 137, 136, 108, 46, 214, 17, 10, 110, 88, 125, 182, 33, 203, 225, 68, 213, 227, 126, 93, 81, 14, 13, 104, 92, 42, 201, 129, 177, 122, 134, 133, 60, 19, 31, 9, 135, 98, 8, 45, 3, 47, 4, 6, 226, 116, 106, 90, 15, 105, 138, 18, 89, 84, 100, 44, 228, 131, 38, 112, 103, 96, 16, 127, 206, 117, 11, 102, 176, 128, 97, 0, 132, 5, 256, 61, 235, 87, 91, 193, 39, 111, 64, 180, 99
gradient boosting	0.731445851	0.7266	138, 244, 224, 137, 118, 103, 230, 204, 42, 13, 86, 3	0.078475997	28, 234, 36, 215, 189, 37, 77, 26, 250, 188, 236, 181, 70, 55, 48, 67, 202, 244, 78, 65, 52, 237, 211, 57, 185, 229, 231, 219, 62, 220, 51,

					200, 30, 242, 233, 198, 34, 25, 212, 205, 252, 32, 29, 248, 253, 75, 66, 247, 56, 58, 12
MLP	0.741557538	0.7404	137, 118, 244, 224, 78, 5, 154, 13, 138, 140, 86, 183, 103, 33, 126, 230, 42, 201, 71, 65, 49, 55, 240, 253, 74, 206, 217, 59, 30, 102, 204, 4, 187, 28, 82, 73, 119, 84, 123, 52, 199, 214, 203, 190, 24, 202, 3, 44, 251, 61, 189, 0, 255, 182, 134, 9, 209	0.002069138	28, 234, 36, 215, 189, 37, 77, 26, 250, 188, 236, 181, 70, 55, 48, 67, 202, 244, 78, 65, 52, 237, 211, 57, 185, 229, 231, 219, 62, 220, 51, 200, 30, 242, 233, 198, 34, 25, 212, 205, 252, 32, 29, 248, 253, 75, 66, 247, 56, 58, 12, 221, 41, 197, 7, 63, 217, 1, 251, 199, 114, 49, 24, 50, 101, 80, 186, 232, 207, 130, 79, 115, 43, 243, 190, 137, 136, 108, 46, 214, 17, 10, 110, 88, 125, 182, 33, 203, 225, 68, 213, 227, 126, 93, 81, 14, 13, 104, 92, 42, 201, 129, 177, 122, 134, 133, 60, 19, 31, 9, 135, 98, 8, 45, 3, 47, 4, 6, 226, 116, 106, 90, 15, 105, 138, 18, 89, 84, 100, 44, 228, 131, 38, 112, 103, 96, 16, 127, 206, 117, 11, 102, 176, 128
logistic regression	0.754261739	0.7376	224, 3, 204, 138, 88, 21, 183, 22, 227, 142, 24, 244, 13, 103, 119, 184, 86, 26, 38, 155, 229, 222, 122, 253, 126, 174, 25, 82, 77, 137, 157, 182, 42, 216, 49, 232, 115, 133, 196, 231, 217, 90, 210, 116, 202, 59, 156, 109, 65, 20, 52, 201, 113, 68, 243, 214, 206, 185, 57, 197, 78, 64, 1, 221, 69, 80, 91, 11, 176, 29, 35, 123, 117, 33, 67, 147, 215, 61, 154, 207, 51, 252, 62, 190, 179, 30, 124, 167, 148, 28, 203, 168, 53, 146, 5	0.379269019	28, 234, 36, 215, 189, 37, 77, 26, 250, 188, 236, 181, 70, 55, 48, 67, 202, 244, 78, 65, 52, 237, 211, 57, 185, 229, 231, 219, 62, 220, 51, 200, 30, 242, 233, 198, 34, 25, 212, 205, 252, 32, 29, 248, 253, 75, 66, 247, 56, 58, 12, 221, 41, 197, 7, 63, 217, 1, 251, 199, 114, 49, 24, 50, 101, 80, 186, 232, 207, 130, 79, 115, 43, 243, 190, 137, 136, 108, 46, 214, 17, 10, 110, 88, 125, 182, 33, 203, 225, 68, 213, 227, 126, 93, 81, 14, 13, 104, 92, 42, 201, 129, 177, 122, 134, 133, 60, 19, 31, 9, 135, 98, 8, 45, 3, 47, 4, 6, 226, 116, 106, 90, 15, 105, 138, 18, 89, 84, 100, 44, 228, 131, 38, 112, 103, 96, 16, 127, 206, 117, 11, 102, 176, 128, 97, 0, 132, 5, 256, 61, 235, 87, 91, 193, 39, 111, 64, 180, 99, 95, 179, 35, 191, 246, 94, 238, 109, 22, 249, 187, 204, 245, 174, 53, 40, 86, 107,

					119, 222, 239, 183, 157, 141, 123, 20, 196, 85, 83, 82, 167, 23, 139, 241, 72, 159, 2, 192, 175, 223, 54, 156, 73, 69, 208, 161, 120, 195, 158, 166, 59, 153, 147, 160, 172, 216, 173, 165, 76, 27, 178, 74, 209, 255, 224, 124, 194, 164, 171, 155, 254, 184, 113, 143, 71, 230, 144, 146, 210, 21, 240, 170, 218, 149, 163, 154
Baysian network	0.5892	0.6124	254, 224, 3, 208, 241, 202, 25, 67, 74, 210, 216, 60, 246, 245, 53, 66, 48, 33, 57, 229, 212, 81, 88, 102, 7, 78, 58, 244, 207, 181, 112, 232, 76, 197, 13, 52	LASSO-alpha	198, 11, 244, 103, 55, 45, 253, 234, 177, 200, 39, 247, 248, 43, 62, 112, 97, 80, 98, 6, 217, 29, 7, 87, 86, 5, 231, 102, 232, 137, 213, 227, 111

Hyperparameter Tuning Results

Class 1

Decision tree:

feature_indexes = [37, 30, 25, 26, 29, 28, 32, 38, 36, 10]

Best Average AUC: 0.7339 ± 0.0437

Average Accuracy: 0.7457 ± 0.0316

Average Precision: 0.1932 ± 0.0333

Average F1 Score: 0.2855 ± 0.0438

Best Hyperparameters:

classifier__criterion: entropy

classifier__max_depth: 19

classifier__max_features: None

classifier__min_samples_leaf: 4

classifier__min_samples_split: 5

classifier__splitter: best

sampler: RandomOverSampler()

sampler__sampling_strategy: 0.75

=== Optimized Threshold ===

Average AUPRC: 0.2390 ± 0.0520

Optimal Threshold (F1-maximizing): 0.6289

F1 Score: 0.2959 ± 0.0576

Accuracy: 0.8431 ± 0.0206

Precision: 0.2535 ± 0.0550

Sensitivity (Recall): 0.3581 ± 0.0628

Specificity: 0.8918 ± 0.0194

Random Forest:

Feature_indexes (relief): [37, 30, 25, 26, 29, 28, 32, 38, 36, 10, 7, 13, 33, 12, 34, 24, 14, 18, 3, 15, 6, 16, 8, 11, 17, 4, 1, 9, 19, 31, 5, 0, 20, 35, 22, 23, 2, 27, 21]

Best Average AUC: 0.7810 ± 0.0204

Average Accuracy: 0.8672 ± 0.0078

Average Precision: 0.3042 ± 0.0311

Average F1 Score: 0.3256 ± 0.0339

Best Hyperparameters:

classifier__bootstrap: False

classifier__criterion: gini

classifier__max_depth: 30

classifier__max_features: log2

classifier__min_samples_leaf: 2

classifier__min_samples_split: 2

classifier__n_estimators: 250

sampler: ADASYN()

sampler__sampling_strategy: auto

=== Optimized Threshold ===

Threshold for Maximum F1 Score: 0.32

F1 Score: 0.3274 ± 0.0311

Accuracy: 0.8212 ± 0.0103

Precision: 0.2495 ± 0.0237

Feature index (LASSO): [24, 20, 16, 15, 12, 3, 10, 13, 21, 9, 22, 28, 29, 19, 38, 2, 6, 26, 31, 5, 33, 11, 35, 34, 1, 8, 36, 32, 30, 23, 14, 0, 4, 27, 37, 18, 17]

Best Average AUC: 0.7814 ± 0.0223

Average Accuracy: 0.8702 ± 0.0073

Average Precision: 0.3067 ± 0.0313

Average F1 Score: 0.3185 ± 0.0318

Best Hyperparameters:

classifier__bootstrap: False

classifier__criterion: gini

classifier__max_depth: None

classifier__max_features: log2

classifier__min_samples_leaf: 2

classifier__min_samples_split: 5

classifier__n_estimators: 200

sampler: SMOTE()

sampler__sampling_strategy: 0.75

=== Optimized Threshold ===

Optimal Threshold (F1-maximizing): 0.3605

Average AUPRC: 0.2788 ± 0.0406

F1 Score: 0.3384 ± 0.0224

Accuracy: 0.8413 ± 0.0082

Precision: 0.2734 ± 0.0178

Sensitivity (Recall): 0.4451 ± 0.0382

Specificity: 0.8811 ± 0.0096

SVM:

Feature indexes (relief): [37, 30, 25, 26, 29, 28, 32, 38, 36, 10, 7, 13, 33, 12, 34, 24, 14, 18, 3, 15, 6, 16, 8, 11, 17, 4, 1, 9, 19, 31, 5, 0, 20, 35, 22, 23, 2]

Best Average AUC: 0.7386 ± 0.0259

Average Accuracy: 0.7730 ± 0.0095

Average Precision: 0.2152 ± 0.0143

Average F1 Score: 0.3111 ± 0.0207

Best Hyperparameters:

classifier__C: 125

classifier__class_weight: None

classifier__gamma: scale

classifier__kernel: rbf

classifier__tol: 0.001

sampler: RandomOverSampler

sampler__sampling_strategy: 0.75

=== Optimized Threshold ===

Optimal Threshold (F1-maximizing): 0.6612

Average AUPRC: 0.2494 ± 0.0412

F1 Score: 0.3104 ± 0.0244

Accuracy: 0.8227 ± 0.0112

Precision: 0.2411 ± 0.0184

Sensitivity (Recall): 0.4378 ± 0.0452

Specificity: 0.8613 ± 0.0137

Feature Indexes (LASSO): [24, 20, 16, 38, 15, 12, 3, 10, 13, 21, 9, 22, 19, 26, 28, 33, 2, 29, 6, 5, 35, 32, 30, 11, 31, 25, 8, 1, 23, 37, 27, 14, 0, 4, 18, 17, 36]

Best Average AUC: 0.7279 ± 0.0302

Average Accuracy: 0.7801 ± 0.0148

Average Precision: 0.2123 ± 0.0196

Average F1 Score: 0.3008 ± 0.0257

Best Hyperparameters:

classifier__C: 100

classifier__class_weight: None

classifier__gamma: scale

classifier__kernel: rbf

classifier__max_iter: 10000

classifier__tol: 0.001

sampler: RandomOverSampler

sampler__sampling_strategy: 0.75

=== Optimized Threshold ===

Threshold for Maximum F1 Score: 0.47

F1 Score: 0.3027 ± 0.0261

Accuracy: 0.7843 ± 0.0088

Precision: 0.2148 ± 0.0175

KNN:

Feature Indexes (relief): [37, 30, 25, 26, 29, 28, 32, 38, 36, 10, 7, 13, 33, 12, 34, 24, 14, 18, 3, 15, 6, 16, 8, 11, 17, 4, 1, 9, 19, 31, 5, 0, 20, 35, 22, 23]

Best Average AUC: 0.7600 ± 0.0165

Average Accuracy: 0.7572 ± 0.0156

Average Precision: 0.2104 ± 0.0196

Average F1 Score: 0.3116 ± 0.0270

Best Hyperparameters:

classifier__algorithm: kd_tree

classifier__leaf_size: 50

classifier__metric: minkowski

classifier__n_neighbors: 28

classifier__p: 1

classifier__weights: distance

oversampler: RandomOverSampler()

oversampler__sampling_strategy: 0.9

=== Optimized Threshold ===

Optimal Threshold (F1-maximizing): 0.6068

Average AUPRC: 0.2583 ± 0.0406

F1 Score: 0.3276 ± 0.0329

Accuracy: 0.8139 ± 0.0118

Precision: 0.2447 ± 0.0255

Sensitivity (Recall): 0.4962 ± 0.0494

Specificity: 0.8458 ± 0.0110

Feature Indexes (LASSO): [24, 20, 16, 15, 12, 3, 10, 13, 21, 9, 22, 28, 29, 19, 38, 2, 6, 26, 31, 5, 33, 11, 35, 34, 1, 8, 36, 32, 30, 23, 14, 0, 4, 27, 37, 18, 17]

Best Average AUC: 0.7594 ± 0.0223

Average Accuracy: 0.7305 ± 0.0156

Average Precision: 0.1980 ± 0.0142

Average F1 Score: 0.3021 ± 0.0195

Best Hyperparameters:

classifier__algorithm: brute

classifier__leaf_size: 15

classifier__metric: manhattan

classifier__n_neighbors: 24

classifier__weights: distance

oversampler: RandomOverSampler()

oversampler__sampling_strategy: auto

=== Optimized Threshold ===

Threshold for Maximum F1 Score: 0.76

F1 Score: 0.3214 ± 0.0506

Accuracy: 0.8620 ± 0.0127

Precision: 0.2930 ± 0.0492

Naïve Bayes:

Feature Indexes (LASSO): [24]

Best Average AUC: 0.6620 ± 0.0440

Average Accuracy: 0.8524 ± 0.0129

Average Precision: 0.2111 ± 0.0520

Average F1 Score: 0.2154 ± 0.0488

Best Hyperparameters:

classifier: GaussianNB()

classifier__var_smoothing: 1e-12

sampler: RandomOverSampler()

sampler__sampling_strategy: 0.5

=== Optimized Threshold ===

Threshold for Maximum F1 Score: 0.30

F1 Score: 0.2420 ± 0.0462

Accuracy: 0.7617 ± 0.0152

Precision: 0.1705 ± 0.0325

Feature Indexes (relief): [37, 30, 25, 26, 29, 28, 32, 38, 36, 10, 7, 13, 33, 12, 34, 24, 14, 18, 3, 15, 6]

Best Average AUC: 0.6626 ± 0.0414

Average Accuracy: 0.8215 ± 0.0181

Average Precision: 0.2062 ± 0.0438

Average F1 Score: 0.2532 ± 0.0495

Best Hyperparameters:

classifier: GaussianNB()

classifier__var_smoothing: 1e-11

sampler: RandomOverSampler()

sampler__sampling_strategy: 0.5

=== Optimized Threshold ===

Optimal Threshold (F1-maximizing): 0.4740

Average AUPRC: 0.1788 ± 0.0337

F1 Score: 0.2703 ± 0.0458

Accuracy: 0.8157 ± 0.0216

Precision: 0.2139 ± 0.0428

Sensitivity (Recall): 0.3706 ± 0.0544

Specificity: 0.8604 ± 0.0213

Adaboost:

Feature Indexes (LASSO): [24, 20, 16, 15, 12, 3, 10, 13, 21, 9, 22, 28, 29, 19, 38, 2, 6, 26, 31]

Best Average AUC: 0.7704 ± 0.0232

Average Accuracy: 0.8725 ± 0.0064

Average Precision: 0.3036 ± 0.0314

Average F1 Score: 0.3044 ± 0.0331

Best Hyperparameters:

ada__algorithm: SAMME

ada__estimator__max_depth: 25

ada__estimator__max_features: sqrt

ada__estimator__min_samples_leaf: 1

ada__estimator__min_samples_split: 5

ada__learning_rate: 0.0025

ada__n_estimators: 300

sampler: ADASYN()

sampler__sampling_strategy: 0.5

=== Optimized Threshold ===

Threshold for Maximum F1 Score: 0.34

F1 Score: 0.3261 ± 0.0281

Accuracy: 0.8368 ± 0.0103

Precision: 0.2622 ± 0.0249

Feature Indexes (relief): [37, 30, 25, 26, 29, 28, 32, 38, 36, 10, 7, 13, 33, 12, 34, 24, 14, 18, 3, 15, 6, 16, 8, 11]

Best Average AUC: 0.7711 ± 0.0221

Average Accuracy: 0.8306 ± 0.0098

Average Precision: 0.2530 ± 0.0223

Average F1 Score: 0.3201 ± 0.0285

Best Hyperparameters:

ada__algorithm: SAMME

ada__estimator__max_depth: 15

ada__estimator__max_features: sqrt

ada__estimator__min_samples_leaf: 2

ada__estimator__min_samples_split: 3

ada__learning_rate: 0.001

ada__n_estimators: 400

sampler: ADASYN()

sampler__sampling_strategy: 0.5

=== Optimized Threshold ===

Optimal Threshold (F1-maximizing): 0.4211

Average AUPRC: 0.2588 ± 0.0434

F1 Score: 0.3263 ± 0.0314

Accuracy: 0.8094 ± 0.0162

Precision: 0.2417 ± 0.0250

Sensitivity (Recall): 0.5050 ± 0.0535

Specificity: 0.8400 ± 0.0181

Gradient Boosting:

Feature Indexes (LASSO): [24, 20, 16, 15, 12, 3, 10, 13, 21, 9, 22, 28, 29, 19, 38, 2, 6, 26, 31, 5, 33, 11, 35, 34, 1, 8, 36]

Best Average AUC: 0.7564 ± 0.0209

Average Accuracy: 0.8696 ± 0.0104

Average Precision: 0.2960 ± 0.0449

Average F1 Score: 0.3005 ± 0.0414

Best Hyperparameters:

gbc__learning_rate: 0.05

gbc__loss: exponential

gbc__max_depth: None

gbc__max_features: log2

gbc__min_samples_leaf: 1

gbc__min_samples_split: 14

gbc__n_estimators: 75

gbc__subsample: 0.4

resampler: RandomOverSampler()

resampler__sampling_strategy: auto

=== Optimized Threshold ===

Optimal Threshold (F1-maximizing): 0.2179

Average AUPRC: 0.2588 ± 0.0446

F1 Score: 0.3304 ± 0.0251

Accuracy: 0.8359 ± 0.0097

Precision: 0.2638 ± 0.0217

Sensitivity (Recall): 0.4433 ± 0.0352

Specificity: 0.8754 ± 0.0101

Feature Indexes (relief): [37, 30, 25, 26, 29, 28, 32, 38, 36, 10, 7, 13, 33, 12]

Best Average AUC: 0.7337 ± 0.0433

Average Accuracy: 0.8073 ± 0.0198

Average Precision: 0.2198 ± 0.0433

Average F1 Score: 0.3039 ± 0.0530

Best Hyperparameters:

gbc__learning_rate: 0.05

gbc__loss: exponential

gbc__max_depth: 6

gbc__max_features: None

gbc__min_samples_leaf: 2

gbc__min_samples_split: 2

gbc__n_estimators: 200

gbc__subsample: 0.8

resampler: RandomOverSampler()

resampler__sampling_strategy: 0.5

=== Optimized Threshold ===

Threshold for Maximum F1 Score: 0.47

F1 Score: 0.2849 ± 0.0522

Accuracy: 0.8033 ± 0.0200

Precision: 0.2138 ± 0.0388

Multi-layer Perceptron:

Feature Indexes: [24, 20, 16, 15, 12, 3, 10, 13, 21, 22, 9, 28, 29, 19, 31, 2, 6, 5, 26, 34, 33, 36, 11, 1, 27, 37, 8, 25, 14, 23, 0, 35, 4, 32, 30, 18]

Best Average AUC: 0.7359 ± 0.0412

Average Accuracy: 0.8900 ± 0.0090

Average Precision: 0.3459 ± 0.0667

Average F1 Score: 0.2682 ± 0.0597

Best Hyperparameters:

classifier__activation: relu

classifier__alpha: 1e-06

classifier__batch_size: auto

classifier__early_stopping: False

classifier__hidden_layer_sizes: (100,)

classifier__learning_rate: adaptive

classifier__learning_rate_init: 0.0005

classifier__max_iter: 1500

classifier__solver: adam

sampler: RandomOverSampler()

sampler__sampling_strategy: 0.25

=== Optimized Threshold ===

Threshold for Maximum F1 Score: 0.38

F1 Score: 0.3112 ± 0.0510

Accuracy: 0.8602 ± 0.0196

Precision: 0.2881 ± 0.0525

Feature Indexes (relief): [37, 30, 25, 26, 29, 28, 32, 38, 36, 10, 7, 13, 33, 12, 34, 24, 14, 18, 3, 15, 6, 16, 8, 11, 17, 4, 1, 9, 19, 31, 5, 0, 20, 35, 22, 23]

Best Average AUC: 0.7483 ± 0.0208

Average Accuracy: 0.7288 ± 0.0300

Average Precision: 0.1929 ± 0.0165

Average F1 Score: 0.2923 ± 0.0189

Best Hyperparameters:

classifier__activation: relu

classifier__alpha: 1e-05

classifier__batch_size: auto

classifier__early_stopping: True

classifier__hidden_layer_sizes: (150, 75, 35)

classifier__learning_rate: constant

classifier__learning_rate_init: 0.001

classifier__max_iter: 1000

classifier__solver: adam

sampler: RandomOverSampler()

sampler__sampling_strategy: 1.0

=== Optimized Threshold ===

Optimal Threshold (F1-maximizing): 0.7091

Average AUPRC: 0.2610 ± 0.0337

F1 Score: 0.3178 ± 0.0486

Accuracy: 0.8434 ± 0.0177

Precision: 0.2660 ± 0.0410

Sensitivity (Recall): 0.4005 ± 0.0705

Specificity: 0.8878 ± 0.0198

Bayesian Network:

Feature Indexes (lasso): [24, 30, 28, 9, 3, 12, 13, 8, 2, 10, 7, 6, 27, 25, 36, 20, 33, 19, 15]

scoring_method BIC

max_parents NaN

oversampler passthrough

sampling_rate NaN

avg_auc 0.649448

std_auc 0.038856

AUPRC: 0.1359 ± 0.0196

Optimal Threshold: 0.1015

F1 Score: 0.2401 ± 0.0401

Accuracy: 0.6837 ± 0.0662

Precision: 0.1555 ± 0.0281

Sensitivity: 0.5423 ± 0.1117

Specificity: 0.6979 ± 0.0789

Feature Indexes (relief): [29, 15, 10, 5, 11, 36, 12, 3, 38, 33, 2, 16, 14, 13, 4, 7, 6, 24]

scoring_method BIC

max_parents NaN

oversampler passthrough

sampling_rate NaN

avg_auc 0.649448

std_auc 0.038856

Accuracy: 0.7021 ± 0.0551

Precision: 0.1629 ± 0.0255

F1 Score: 0.2484 ± 0.0310

Logistic Regression:

Feature Indexes (lasso): [24, 38, 20, 16, 15, 12, 3, 10, 13, 21, 9, 22, 26, 33, 19, 2, 35, 32, 28, 6, 30]

Best Average AUC: 0.6739 ± 0.0469

Average Accuracy: 0.6657 ± 0.0219

Average Precision: 0.1552 ± 0.0202

Average F1 Score: 0.2463 ± 0.0315

Best Hyperparameters:

lr__C: 30

lr__l1_ratio: None

lr__penalty: l2

lr__solver: sag

resampler: RandomOverSampler()

resampler__sampling_strategy: auto

=== Optimized Threshold ===

Optimal Threshold (F1-maximizing): 0.6390

Average AUPRC: 0.2031 ± 0.0460

F1 Score: 0.2764 ± 0.0603

Accuracy: 0.8338 ± 0.0169

Precision: 0.2303 ± 0.0512

Sensitivity (Recall): 0.3474 ± 0.0783

Specificity: 0.8827 ± 0.0143

Feature Indexes (relief): [37, 30, 25, 26, 29, 28, 32, 38, 36, 10, 7, 13, 33, 12, 34, 24, 14, 18, 3, 15, 6, 16, 8, 11, 17, 4, 1, 9, 19, 31, 5, 0, 20, 35, 22, 23, 2, 27, 21]

Best Average AUC: 0.6668 ± 0.0509

Average Accuracy: 0.8547 ± 0.0148

Average Precision: 0.2352 ± 0.0604

Average F1 Score: 0.2459 ± 0.0612

Best Hyperparameters:

lr__C: 0.1

lr__l1_ratio: 0.5

lr__penalty: elasticnet

lr__solver: saga

resampler: SMOTE()

resampler__sampling_strategy: 0.5

=== Optimized Threshold ===

Threshold for Maximum F1 Score: 0.43

F1 Score: 0.2724 ± 0.0432

Accuracy: 0.8133 ± 0.0148

Precision: 0.2119 ± 0.0344

TSGNN:

global relief

Best trial:

Value (Mean AUC): 0.7455567712393181

Params:

n_epochs: 1175

lr: 0.002652887105202934

weight_decay: 1.1942523033711955e-07

batch_size: 64

n_genotype: 12

n_history: 5

n_phenotype: 11

n_behaviour: 4

Average AUC: 0.7456 ± 0.0312

Average F1 Score: 0.1408 ± 0.0673

Average Accuracy: 0.9058 ± 0.0083

Average Precision: 0.4434 ± 0.2184

Optimal Threshold: 0.1703

AUPRC: 0.2668 ± 0.0566

F1 Score: 0.3187 ± 0.0410

Accuracy: 0.8223 ± 0.0209

Precision: 0.2471 ± 0.0344

Sensitivity (Recall): 0.4553 ± 0.0713

Specificity: 0.8591 ± 0.0252

Separate relief:

Best trial:

Value (Mean AUC): 0.7394404100047507

Params:

n_epochs: 2080

lr: 0.0003314586104440468

weight_decay: 0.0005417687953694567

batch_size: 128

n_genotype: 6

n_history: 2

n_phenotype: 13

n_behaviour: 1

Average AUC: 0.7394 ± 0.0405

Average F1 Score: 0.0334 ± 0.0297

Average Accuracy: 0.9079 ± 0.0031

Average Precision: 0.4571 ± 0.4554

Average F1 Score: 0.3331 ± 0.0619

Average Accuracy: 0.8162 ± 0.0740

Average Precision: 0.2721 ± 0.0782

Global lasso:

Best trial:

Value (Mean AUC): 0.7377684524348088

Params:

n_epochs: 1042

lr: 0.002295940611801856

weight_decay: 1.3988230833611534e-07

batch_size: 32

n_genotype: 13

n_history: 6

n_phenotype: 12

n_behaviour: 5

Average AUC: 0.7378 ± 0.0317

Average F1 Score: 0.1673 ± 0.0557

Average Accuracy: 0.9073 ± 0.0062

Average Precision: 0.4841 ± 0.1696

Average F1 Score: 0.3375 ± 0.0662

Average Accuracy: 0.8424 ± 0.0609

Average Precision: 0.3393 ± 0.1543

Separate lasso:

Best trial:

Value (Mean AUC): 0.7433285096690063

Params:

n_epochs: 1175

lr: 0.0004464002613441988

weight_decay: 1.4153526263149175e-08

batch_size: 128

n_genotype: 10

n_history: 3

n_phenotype: 13

n_behaviour: 2

Average AUC: 0.7433 ± 0.0368

Average F1 Score: 0.0718 ± 0.0559

Average Accuracy: 0.9073 ± 0.0049

Average Precision: 0.3878 ± 0.3015

Average F1 Score: 0.3459 ± 0.0665

Average Accuracy: 0.8327 ± 0.0723

Average Precision: 0.3171 ± 0.1140

TSNN:

global relief

Best Trial:

AUC: 0.7336598151821246 ± 0.0361

Params:

n_genotype: 14

n_history: 5

n_phenotype: 10

n_behaviour: 4

learning_rate: 0.0014641867315396168

epochs: 1546

batch_size: 16

Best Threshold to maximize F1 Score: 0.16

F1 Score: 0.3010 ± 0.0464

Accuracy: 0.8230 ± 0.0178

Precision: 0.2363 ± 0.0384

separate relief

Best Trial:

AUC: 0.7357700263311236

Params:

n_genotype: 13

n_history: 3

n_phenotype: 11

n_behaviour: 3

learning_rate: 0.0013776008234454911

epochs: 2382

batch_size: 32

Optimal Threshold: 0.1833

AUPRC: 0.2508 ± 0.0570

F1 Score: 0.3102 ± 0.0605

Accuracy: 0.8487 ± 0.0121

Precision: 0.2653 ± 0.0476

Sensitivity (Recall): 0.3756 ± 0.0836

Specificity: 0.8962 ± 0.0110

global lasso:

Best Trial:

AUC: 0.7242 (±0.0508)

F1 Score: 0.0411 (Std: 0.0449)

Accuracy: 0.9068 (Std: 0.0029)

Precision: 0.1867 (Std: 0.2059)

Params:

n_genotype: 12

n_history: 6

n_phenotype: 10

n_behaviour: 1

learning_rate: 0.0007849256583952668

epochs: 1674

batch_size: 64

Best Threshold to maximize F1 Score: 0.68

F1 Score: 0.2874 ± 0.0547

Accuracy: 0.8194 ± 0.0244

Precision: 0.2273 ± 0.0500

separate lasso:

Best Trial:

AUC: 0.7293 (±0.0259)

F1 Score: 0.0192 (±0.0321)

Accuracy: 0.9058 (±0.0024)

Precision: 0.1550 (±0.3020)

Params:

n_genotype: 13

n_history: 5

n_phenotype: 10

n_behaviour: 2

learning_rate: 0.0009955474543238663

epochs: 1353

batch_size: 64

Best Threshold to maximize F1 Score: 0.15

F1 Score: 0.2984 ± 0.0445

Accuracy: 0.8059 ± 0.0200

Precision: 0.2239 ± 0.0372

Class 1-3

Decision Tree

Feature Indexes (relief): [28, 234, 36, 215, 189, 37, 77, 26, 250, 188, 236, 181, 70, 55, 48, 67, 202, 244, 78, 65, 52, 237, 211, 57, 185, 229, 231, 219, 62, 220, 51, 200, 30, 242, 233, 198, 34, 25, 212, 205, 252, 32, 29, 248, 253, 75, 66, 247, 56, 58, 12, 221, 41, 197, 7, 63, 217, 1, 251, 199]

Best Average AUC: 0.7337 ± 0.0400

Average Accuracy: 0.8966 ± 0.0101

Average Precision: 0.3636 ± 0.1096

Average F1 Score: 0.2262 ± 0.0686

Best Hyperparameters:

classifier__criterion: entropy

classifier__max_depth: 21

classifier__max_features: sqrt

classifier__min_samples_leaf: 2

classifier__min_samples_split: 7

classifier__splitter: best

sampler: RandomOverSampler()

sampler__sampling_strategy: 0.25

=== Optimized Threshold ===

Average AUPRC: 0.2303 ± 0.0437

Optimal Threshold (F1-maximizing): 0.3676

F1 Score: 0.2948 ± 0.0586

Accuracy: 0.8478 ± 0.0208

Precision: 0.2595 ± 0.0625

Sensitivity (Recall): 0.3438 ± 0.0527

Specificity: 0.8984 ± 0.0188

Random Forest

Feature indexes (lasso): [138, 244, 137, 224, 118, 103, 230, 42, 78, 204, 13, 33, 86, 123, 24, 3, 5, 21, 183, 126, 119, 65, 201, 30, 253, 122, 88, 49, 210, 80, 214, 217, 52, 206, 173, 90, 44, 59, 71, 229, 25, 157, 68, 247, 227, 182, 28]

Best Average AUC: 0.7825 ± 0.0206

Average Accuracy: 0.8830 ± 0.0059

Average Precision: 0.3273 ± 0.0365

Average F1 Score: 0.2942 ± 0.0357

Best Hyperparameters:

classifier__bootstrap: False

classifier__class_weight: None

classifier__criterion: gini

classifier__max_depth: None

classifier__max_features: sqrt

classifier__min_samples_leaf: 2

classifier__min_samples_split: 2

classifier__n_estimators: 290

sampler: ADASYN()

sampler__sampling_strategy: 0.25

=== Optimized Threshold ===

Threshold for Maximum F1 Score: 0.30

F1 Score: 0.3328 ± 0.0315

Accuracy: 0.8448 ± 0.0095

Precision: 0.2744 ± 0.0282

Feature indexes (relief): [28, 234, 36, 215, 189, 37, 77, 26, 250, 188, 236, 181, 70, 55, 48, 67, 202, 244, 78, 65, 52, 237, 211, 57, 185, 229, 231, 219, 62, 220, 51, 200, 30, 242, 233, 198, 34, 25, 212, 205, 252, 32, 29, 248, 253, 75, 66, 247, 56, 58, 12, 221, 41, 197, 7, 63, 217, 1, 251, 199, 114, 49, 24, 50, 101, 80, 186, 232, 207, 130, 79, 115, 43, 243, 190, 137, 136, 108, 46, 214, 17, 10, 110, 88, 125, 182, 33, 203, 225, 68, 213, 227, 126, 93, 81, 14, 13, 104, 92, 42, 201, 129, 177, 122, 134, 133, 60, 19, 31, 9, 135, 98, 8, 45, 3, 47, 4, 6, 226, 116, 106, 90, 15, 105, 138, 18, 89, 84, 100, 44, 228, 131, 38, 112, 103, 96, 16, 127, 206, 117, 11, 102, 176, 128, 97, 0, 132, 5, 256, 61, 235, 87, 91, 193, 39, 111, 64, 180, 99, 95, 179, 35, 191, 246, 94, 238, 109, 22, 249, 187, 204, 245, 174, 53, 40, 86, 107, 119, 222, 239, 183, 157, 141, 123, 20, 196, 85, 83, 82, 167, 23, 139, 241, 72, 159, 2, 192, 175, 223, 54, 156, 73, 69, 208, 161, 120, 195, 158]

Best Average AUC: 0.7842 ± 0.0189

Average Accuracy: 0.8845 ± 0.0056

Average Precision: 0.3068 ± 0.0497

Average F1 Score: 0.2532 ± 0.0564

Best Hyperparameters:

classifier__bootstrap: False

classifier__class_weight: None

classifier__criterion: entropy

classifier__max_depth: None

classifier__max_features: sqrt

classifier__min_samples_leaf: 2

classifier__min_samples_split: 7

classifier__n_estimators: 225

sampler: RandomOverSampler()

sampler__sampling_strategy: auto

=== Optimized Threshold ===

Optimal Threshold (F1-maximizing): 0.1938

Average AUPRC: 0.2734 ± 0.0402

F1 Score: 0.3408 ± 0.0348

Accuracy: 0.8350 ± 0.0110

Precision: 0.2686 ± 0.0270

Sensitivity (Recall): 0.4680 ± 0.0547

Specificity: 0.8718 ± 0.0111

Support Vector Machine

Feature indexes (LASSO): [138, 244, 224, 137, 118, 103, 204, 230, 3, 86, 42, 24, 13, 21, 78, 33, 88, 183, 123, 119, 126, 210, 122, 65, 201, 5, 253, 30, 109, 227, 90, 49, 214, 52, 80, 25, 247, 217, 22, 229, 157, 115]

Best Average AUC: 0.7500 ± 0.0193

Average Accuracy: 0.8882 ± 0.0100

Average Precision: 0.3663 ± 0.0599

Average F1 Score: 0.3294 ± 0.0515

Best Hyperparameters:

classifier__C: 10

classifier__class_weight: None

classifier__coef0: 0.5

classifier__degree: 5

classifier__gamma: scale

classifier__kernel: poly

classifier__max_iter: 20000

classifier__tol: 1e-05

sampler: RandomOverSampler

sampler__sampling_strategy: 0.25

=== Optimized Threshold ===

Optimal Threshold (F1-maximizing): 0.2328

Average AUPRC: 0.2746 ± 0.0406

F1 Score: 0.3462 ± 0.0336

Accuracy: 0.8426 ± 0.0128

Precision: 0.2799 ± 0.0322

Sensitivity (Recall): 0.4556 ± 0.0422

Specificity: 0.8814 ± 0.0133

Feature Indexes (relief): [28, 234, 36, 215, 189, 37, 77, 26, 250, 188, 236, 181, 70, 55, 48, 67, 202, 244, 78, 65, 52, 237, 211, 57, 185, 229, 231, 219, 62, 220, 51, 200, 30, 242, 233, 198, 34, 25, 212, 205, 252, 32, 29, 248, 253, 75, 66, 247, 56, 58, 12, 221, 41, 197, 7, 63, 217, 1, 251, 199, 114, 49, 24, 50, 101, 80, 186, 232, 207, 130, 79, 115, 43, 243, 190, 137, 136, 108, 46, 214, 17, 10, 110, 88, 125, 182, 33, 203, 225, 68, 213, 227, 126, 93, 81, 14, 13, 104, 92, 42, 201, 129, 177, 122, 134, 133, 60, 19, 31, 9, 135, 98, 8, 45, 3, 47, 4, 6, 226, 116, 106, 90, 15, 105, 138, 18, 89, 84, 100, 44, 228, 131, 38, 112, 103, 96, 16, 127, 206, 117, 11, 102, 176, 128, 97, 0, 132, 5, 256, 61, 235, 87, 91, 193, 39, 111, 64, 180, 99, 95, 179, 35, 191, 246, 94, 238, 109, 22, 249, 187, 204, 245, 174, 53, 40, 86, 107, 119, 222, 239, 183, 157, 141, 123, 20, 196, 85, 83, 82, 167, 23, 139, 241, 72, 159, 2, 192, 175, 223]

Best Average AUC: 0.7415 ± 0.0316

Average Accuracy: 0.8076 ± 0.0153

Average Precision: 0.2294 ± 0.0283

Average F1 Score: 0.3072 ± 0.0354

Best Hyperparameters:

classifier__C: 10

classifier__class_weight: balanced

classifier__coef0: 1.0

classifier__degree: 4

classifier__gamma: scale

classifier__kernel: poly

classifier__max_iter: 20000

classifier__tol: 1e-05

sampler: SMOTE

sampler__sampling_strategy: 0.5

=== Optimized Threshold ===

Threshold for Maximum F1 Score: 0.56

F1 Score: 0.3231 ± 0.0734

Accuracy: 0.8544 ± 0.0203

Precision: 0.2835 ± 0.0710

KNN:

feature_indexes (lasso): [138, 244, 224, 137, 118, 103, 230, 204, 42]

Best Average AUC: 0.7592 ± 0.0266

Average Accuracy: 0.9071 ± 0.0031

Average Precision: 0.4590 ± 0.1331

Average F1 Score: 0.1110 ± 0.0262

Best Hyperparameters:

classifier__algorithm: auto

classifier__leaf_size: 50

classifier__metric: manhattan

classifier__n_neighbors: 30

classifier__weights: distance

oversampler: passthrough

=== Optimized Threshold ===

Optimal Threshold (F1-maximizing): 0.1876

Average AUPRC: 0.2693 ± 0.0404

F1 Score: 0.3312 ± 0.0387

Accuracy: 0.8348 ± 0.0146

Precision: 0.2637 ± 0.0345

Sensitivity (Recall): 0.4467 ± 0.0477

Specificity: 0.8738 ± 0.0138

Feature Indexes (relief): [28, 234, 36, 215, 189, 37, 77, 26, 250, 188, 236, 181, 70, 55, 48, 67, 202, 244, 78, 65, 52, 237, 211, 57, 185, 229, 231, 219, 62, 220, 51, 200, 30, 242, 233, 198, 34, 25, 212, 205, 252, 32, 29, 248, 253, 75, 66, 247, 56, 58, 12, 221, 41, 197, 7, 63, 217, 1, 251, 199, 114, 49, 24, 50, 101, 80, 186, 232, 207, 130, 79, 115, 43, 243, 190, 137, 136, 108, 46, 214, 17, 10, 110, 88, 125, 182, 33, 203, 225, 68, 213, 227, 126, 93, 81, 14, 13, 104, 92, 42, 201, 129, 177, 122, 134, 133, 60, 19, 31, 9, 135, 98, 8, 45, 3, 47, 4, 6, 226, 116, 106, 90, 15]

Best Average AUC: 0.7527 ± 0.0291

Average Accuracy: 0.8963 ± 0.0076

Average Precision: 0.3706 ± 0.0821

Average F1 Score: 0.2485 ± 0.0488

Best Hyperparameters:

classifier__algorithm: auto

classifier__leaf_size: 20

classifier__metric: manhattan

classifier__n_neighbors: 49

classifier__weights: distance

oversampler: ADASYN

oversampler__sampling_strategy: 0.2

=== Optimized Threshold ===

Threshold for Maximum F1 Score: 0.29

F1 Score: 0.3371 ± 0.0445

Accuracy: 0.8466 ± 0.0164

Precision: 0.2804 ± 0.0429

Naïve Bayes

Feature Indexes (relief): [28, 234, 36, 215, 189, 37, 77, 26, 250, 188, 236, 181, 70, 55, 48, 67, 202, 244, 78, 65, 52, 237, 211, 57, 185, 229, 231, 219, 62, 220, 51, 200, 30, 242, 233, 198, 34, 25, 212, 205, 252, 32, 29, 248, 253, 75, 66, 247, 56, 58, 12, 221, 41, 197, 7, 63, 217, 1, 251, 199, 114, 49, 24, 50, 101, 80, 186, 232, 207, 130, 79, 115, 43, 243, 190, 137, 136, 108, 46, 214, 17, 10, 110, 88, 125, 182, 33, 203, 225, 68, 213, 227, 126, 93, 81, 14, 13, 104, 92, 42, 201, 129, 177, 122, 134, 133, 60, 19, 31, 9, 135, 98, 8, 45, 3, 47, 4, 6, 226, 116, 106, 90, 15, 105, 138, 18, 89, 84, 100, 44, 228, 131,

38, 112, 103, 96, 16, 127, 206, 117, 11, 102, 176, 128, 97, 0, 132, 5, 256, 61, 235, 87, 91, 193, 39]

Best Average AUC: 0.6977 ± 0.0444

Average Accuracy: 0.6790 ± 0.0257

Average Precision: 0.1623 ± 0.0240

Average F1 Score: 0.2553 ± 0.0357

Best Hyperparameters:

classifier: BernoulliNB()

classifier__alpha: 25.75

classifier__binarize: 0.0

classifier__fit_prior: False

classifier__force_alpha: True

sampler: RandomOverSampler()

sampler__sampling_strategy: 0.5

=== Optimized Threshold ===

Optimal Threshold (F1-maximizing): 0.7810

Average AUPRC: 0.2153 ± 0.0444

F1 Score: 0.2680 ± 0.0392

Accuracy: 0.8330 ± 0.0114

Precision: 0.2237 ± 0.0300

Sensitivity (Recall): 0.3367 ± 0.0590

Specificity: 0.8829 ± 0.0136

Adaboost:

Feature Indexes (lasso): [137, 118, 244, 224, 5, 78, 154, 13, 138, 140, 86, 183, 103, 33, 126, 230, 42, 201, 71, 65, 49, 55, 240, 74, 206, 217, 253, 59, 102, 30, 4, 204, 187, 28, 82, 84, 73, 119, 123, 52, 199, 214, 203, 190, 202]

Best Average AUC: 0.7461 ± 0.0251

Average Accuracy: 0.9073 ± 0.0045

Average Precision: 0.4738 ± 0.1492

Average F1 Score: 0.1357 ± 0.0333

Best Hyperparameters:

ada__algorithm: SAMME

ada__estimator__max_depth: 10

ada__estimator__max_features: log2

ada__estimator__min_samples_leaf: 1

ada__estimator__min_samples_split: 12

ada__learning_rate: 0.01

ada__n_estimators: 250

sampler: passthrough

=== Optimized Threshold ===

Threshold for Maximum F1 Score: 0.24

F1 Score: 0.3279 ± 0.0425

Accuracy: 0.8415 ± 0.0158

Precision: 0.2690 ± 0.0402

Feature Indexes (relief): [28, 234, 36, 215, 189, 37, 77, 26, 250, 188, 236, 181, 70, 55, 48, 67, 202, 244, 78, 65, 52, 237, 211, 57, 185, 229, 231, 219, 62, 220, 51, 200, 30, 242, 233, 198, 34, 25, 212, 205, 252, 32, 29, 248, 253, 75, 66, 247, 56, 58, 12, 221, 41, 197, 7, 63, 217, 1, 251, 199, 114, 49, 24, 50, 101, 80, 186, 232, 207, 130, 79, 115, 43, 243, 190, 137, 136, 108, 46, 214, 17, 10, 110, 88, 125, 182, 33, 203, 225, 68, 213, 227, 126, 93, 81, 14, 13, 104, 92, 42, 201, 129, 177, 122, 134, 133, 60, 19, 31, 9, 135, 98, 8, 45, 3, 47, 4, 6, 226, 116, 106, 90, 15, 105, 138, 18, 89, 84, 100, 44, 228, 131, 38, 112, 103, 96, 16, 127, 206, 117, 11, 102, 176, 128, 97, 0, 132, 5, 256, 61, 235, 87, 91, 193, 39, 111, 64, 180, 99]

Best Average AUC: 0.7811 ± 0.0248

Average Accuracy: 0.8855 ± 0.0090

Average Precision: 0.3202 ± 0.0554

Average F1 Score: 0.2616 ± 0.0476

Best Hyperparameters:

ada__algorithm: SAMME

ada__estimator__max_depth: 20

ada__estimator__max_features: sqrt

ada__estimator__min_samples_leaf: 2

ada__estimator__min_samples_split: 15

ada__learning_rate: 1e-05

ada__n_estimators: 250

sampler: ADASYN

sampler__sampling_strategy: 0.2

=== Optimized Threshold ===

Optimal Threshold (F1-maximizing): 0.2399

Average AUPRC: 0.2664 ± 0.0432

F1 Score: 0.3195 ± 0.0309

Accuracy: 0.7973 ± 0.0166

Precision: 0.2310 ± 0.0257

Sensitivity (Recall): 0.5194 ± 0.0384

Specificity: 0.8252 ± 0.0163

Gradient boosting:

Feature Indexes (lasso): [138, 244, 224, 137, 118, 103, 230, 204, 42, 13, 86, 3]

Best Average AUC: 0.7590 ± 0.0283

Average Accuracy: 0.8796 ± 0.0147

Average Precision: 0.3179 ± 0.0816

Average F1 Score: 0.2908 ± 0.0708

Best Hyperparameters:

gbc__learning_rate: 0.01

gbc__loss: exponential

gbc__max_depth: 20

gbc__max_features: log2

gbc__min_samples_leaf: 2

gbc__min_samples_split: 5

gbc__n_estimators: 125

gbc__subsample: 0.1

resampler: RandomOverSampler

resampler__sampling_strategy: 0.5

=== Optimized Threshold ===

Optimal Threshold (F1-maximizing): 0.4279

Average AUPRC: 0.2684 ± 0.0452

F1 Score: 0.3059 ± 0.0500

Accuracy: 0.8536 ± 0.0138

Precision: 0.2709 ± 0.0450

Sensitivity (Recall): 0.3544 ± 0.0654

Specificity: 0.9037 ± 0.0147

Feature Indexes (relief): [28, 234, 36, 215, 189, 37, 77, 26, 250, 188, 236, 181, 70, 55, 48, 67, 202, 244, 78, 65, 52, 237, 211, 57, 185, 229, 231, 219, 62, 220, 51, 200, 30, 242, 233, 198, 34, 25, 212, 205, 252, 32, 29, 248, 253, 75, 66, 247, 56, 58, 12]

Best Average AUC: 0.7332 ± 0.0428

Average Accuracy: 0.8313 ± 0.0163

Average Precision: 0.2460 ± 0.0176

Average F1 Score: 0.2653 ± 0.0282

Best Hyperparameters:

gbc__learning_rate: 0.1

gbc__loss: exponential

gbc__max_depth: 5

gbc__max_features: sqrt

gbc__min_samples_leaf: 1

gbc__min_samples_split: 2

gbc__n_estimators: 100

gbc__subsample: 0.8

resampler: RandomOverSampler

resampler__sampling_strategy: auto

=== Optimized Threshold ===

Threshold for Maximum F1 Score: 0.29

F1 Score: 0.2893 ± 0.0596

Accuracy: 0.8272 ± 0.0170

Precision: 0.2322 ± 0.0460

MLP:

Feature Indexes (lasso): [137, 118, 244, 224, 78, 5, 154, 13, 138, 140, 86, 183, 103, 33, 126, 230, 42, 201, 71, 65, 49, 55, 240, 253, 74, 206, 217, 59, 30, 102, 204, 4, 187, 28, 82, 73, 119, 84, 123, 52, 199, 214, 203, 190, 24, 202, 3, 44, 251, 61, 189, 0, 255, 182, 134, 9, 209]

Best Average AUC: 0.7429 ± 0.0421

Average Accuracy: 0.8102 ± 0.0172

Average Precision: 0.2322 ± 0.0205

Average F1 Score: 0.2724 ± 0.0319

Best Hyperparameters:

classifier__activation: tanh

classifier__alpha: 0.0001

classifier__batch_size: 64

classifier__early_stopping: False

classifier__hidden_layer_sizes: (50, 100, 50)

classifier__learning_rate: adaptive

classifier__learning_rate_init: 0.001

classifier__max_iter: 500

classifier__solver: sgd

sampler: RandomOverSampler

sampler__sampling_strategy: 1.0

=== Optimized Threshold ===

Threshold for Maximum F1 Score: 0.64

F1 Score: 0.3000 ± 0.0441

Accuracy: 0.8057 ± 0.0154

Precision: 0.2239 ± 0.0339

Feature Indexes (relief): [28, 234, 36, 215, 189, 37, 77, 26, 250, 188, 236, 181, 70, 55, 48, 67, 202, 244, 78, 65, 52, 237, 211, 57, 185, 229, 231, 219, 62, 220, 51, 200, 30, 242, 233, 198, 34, 25, 212, 205, 252, 32, 29, 248, 253, 75, 66, 247, 56, 58, 12, 221, 41, 197, 7, 63, 217, 1, 251, 199, 114, 49, 24, 50, 101, 80, 186, 232, 207, 130, 79, 115, 43, 243, 190, 137, 136, 108, 46, 214, 17, 10, 110, 88, 125, 182, 33, 203, 225, 68, 213, 227, 126, 93, 81, 14, 13, 104, 92, 42, 201, 129, 177, 122, 134, 133, 60, 19, 31, 9, 135, 98, 8, 45, 3, 47, 4, 6, 226, 116, 106, 90, 15, 105, 138, 18, 89, 84, 100, 44, 228, 131, 38, 112, 103, 96, 16, 127, 206, 117, 11, 102, 176, 128]

Best Average AUC: 0.7455 ± 0.0406

Average Accuracy: 0.8225 ± 0.0191

Average Precision: 0.2445 ± 0.0234

Average F1 Score: 0.2795 ± 0.0360

Best Hyperparameters:

classifier__activation: tanh

classifier__alpha: 1e-05

classifier__batch_size: auto

classifier__early_stopping: False

classifier__hidden_layer_sizes: (100,)

classifier__learning_rate: constant

classifier__learning_rate_init: 0.0001

classifier__max_iter: 500

classifier__solver: adam

sampler: RandomOverSampler

sampler__sampling_strategy: auto

=== Optimized Threshold ===

Optimal Threshold (F1-maximizing): 0.7083

Average AUPRC: 0.2634 ± 0.0596

F1 Score: 0.3209 ± 0.0543

Accuracy: 0.8492 ± 0.0189

Precision: 0.2754 ± 0.0517

Sensitivity (Recall): 0.3881 ± 0.0651

Specificity: 0.8955 ± 0.0182

Bayesian Network:

Feature Indexes (relief): [29, 15, 10, 5, 11, 36, 12, 3, 38, 33, 2, 16, 14, 13, 4, 7, 6, 24]

Best Hyperparameters Identified:

**scoring_method**	**BIC**
**max_parents**	**NaN**
**oversampler**	**passthrough**
**sampling_rate**	**NaN**

avg_auc 0.649448

std_auc 0.038856

AUPRC: 0.1344 ± 0.0197

Optimal Threshold: 0.1302

F1 Score: 0.2387 ± 0.0383

Accuracy: 0.6615 ± 0.0878

Precision: 0.1533 ± 0.0294

Sensitivity: 0.5677 ± 0.1088

Specificity: 0.6710 ± 0.1037

Logistic Regression:

Feature Indexes (lasso): [224, 3, 204, 138, 88, 21, 183, 22, 227, 142, 24, 244, 13, 103, 119, 184, 86, 26, 38, 155, 229, 222, 122, 253, 126, 174, 25, 82, 77, 137, 157, 182, 42, 216, 49, 232, 115, 133, 196, 231, 217, 90, 210, 116, 202, 59, 156, 109, 65, 20, 52, 201, 113, 68, 243, 214, 206, 185, 57, 197, 78, 64, 1, 221, 69, 80, 91, 11, 176, 29, 35, 123, 117, 33, 67, 147, 215, 61, 154, 207, 51, 252, 62, 190, 179, 30, 124, 167, 148, 28, 203, 168, 53, 146, 5]

Best Average AUC: 0.7620 ± 0.0274

Average Accuracy: 0.8358 ± 0.0153

Average Precision: 0.2622 ± 0.0429

Average F1 Score: 0.3272 ± 0.0494

Best Hyperparameters:

lr__C: 1

lr__l1_ratio: 0.3

lr__penalty: elasticnet

lr__solver: saga

resampler: RandomOverSampler()

resampler__sampling_strategy: 0.5

=== Optimized Threshold ===

Optimal Threshold (F1-maximizing): 0.5458

Average AUC: 0.7505 ± 0.0327

Average AUPRC: 0.2759 ± 0.0592

F1 Score: 0.3278 ± 0.0551

Accuracy: 0.8565 ± 0.0156

Precision: 0.2883 ± 0.0523

Sensitivity (Recall): 0.3826 ± 0.0650

Specificity: 0.9040 ± 0.0149

Feature Indexes (relief): [28, 234, 36, 215, 189, 37, 77, 26, 250, 188, 236, 181, 70, 55, 48, 67, 202, 244, 78, 65, 52, 237, 211, 57, 185, 229, 231, 219, 62, 220, 51, 200, 30, 242, 233, 198, 34, 25, 212, 205, 252, 32, 29, 248, 253, 75, 66, 247, 56, 58, 12, 221, 41, 197, 7, 63, 217, 1, 251, 199, 114, 49, 24, 50, 101, 80, 186, 232, 207, 130, 79, 115, 43, 243, 190, 137, 136, 108, 46, 214, 17, 10, 110, 88, 125, 182, 33, 203, 225, 68, 213, 227, 126, 93, 81, 14, 13, 104, 92, 42, 201, 129, 177, 122, 134, 133, 60, 19, 31, 9, 135, 98, 8, 45, 3, 47, 4, 6, 226, 116, 106, 90, 15, 105, 138, 18, 89, 84, 100, 44, 228, 131, 38, 112, 103, 96, 16, 127, 206, 117, 11, 102, 176, 128, 97, 0, 132, 5, 256, 61, 235, 87, 91, 193, 39, 111, 64, 180, 99, 95, 179, 35, 191, 246, 94, 238, 109, 22, 249, 187, 204, 245, 174, 53, 40, 86, 107, 119, 222, 239, 183, 157, 141, 123, 20, 196, 85, 83, 82, 167, 23, 139, 241, 72, 159, 2, 192, 175, 223, 54, 156, 73, 69, 208, 161, 120, 195, 158, 166, 59, 153, 147, 160, 172, 216, 173, 165, 76, 27, 178, 74, 209, 255, 224, 124, 194, 164, 171, 155, 254, 184, 113, 143, 71, 230, 144, 146, 210, 21, 240, 170, 218, 149, 163, 154]

Best Average AUC: 0.7421 ± 0.0375

Average Accuracy: 0.6895 ± 0.0138

Average Precision: 0.1757 ± 0.0171

Average F1 Score: 0.2766 ± 0.0268

Best Hyperparameters:

lr__C: 1

lr__l1_ratio: None

lr__penalty: l1

lr__solver: saga

resampler: RandomOverSampler()

resampler__sampling_strategy: auto

=== Optimized Threshold ===

Threshold for Maximum F1 Score: 0.70

F1 Score: 0.3072 ± 0.0529

Accuracy: 0.8491 ± 0.0130

Precision: 0.2648 ± 0.0458

TSNN:

Global relief:

Best Trial:

AUC: 0.7531640655561265 ± 0.0330

Params:

n_genotype: 68

n_history: 1

n_phenotype: 59

n_behaviour: 7

learning_rate: 0.0023343464127852007

epochs: 1568

batch_size: 128

AUC: 0.7532 ± 0.0330

F1 Score: 0.1198 ± 0.0304

Accuracy: 0.9055 ± 0.0055

Precision: 0.4348 ± 0.1277

Optimal Threshold: 0.1869

AUC: 0.7405 ± 0.0223

AUPRC: 0.2633 ± 0.0342

F1 Score: 0.3292 ± 0.0394

Accuracy: 0.8340 ± 0.0205

Precision: 0.2633 ± 0.0379

Sensitivity (Recall): 0.4430 ± 0.0438

Specificity: 0.8732 ± 0.0217

separate relief:

Best Trial:

AUC: 0.752471120396032

parameters: {'n_genotype': 1, 'n_history': 7, 'n_phenotype': 52, 'n_behaviour': 4,

'learning_rate': 0.0051175419520232135,

'epochs': 2654,

'batch_size': 256}

AUC: 0.7525 ± 0.0275

F1 Score: 0.1247 ± 0.0445

Accuracy: 0.9065 ± 0.0050

Precision: 0.4666 ± 0.1263

Best Threshold to maximize F1 Score: 0.19

--- Per-Fold Metrics after Threshold Optimization ---

F1 Score: 0.3220 ± 0.0518

Accuracy: 0.8379 ± 0.0171

Precision: 0.2617 ± 0.0450

Global lasso:

Best Trial:

AUC: 0.7417 ± 0.0357

F1 Score: 0.0456 (Std: 0.0528)

Accuracy: 0.9075 (Std: 0.0046)

Precision: 0.3000 (Std: 0.3253)

Params:

n_genotype: 36

n_history: 4

n_phenotype: 8

n_behaviour: 2

learning_rate: 0.00013536078428508602

epochs: 2045

batch_size: 16

Best Threshold to maximize F1 Score: 0.60

F1 Score: 0.2816 ± 0.0400

Accuracy: 0.7588 ± 0.0279

Precision: 0.1948 ± 0.0325

Separate lasso:

Best Trial:

AUC: 0.7311 ± 0.0484

F1 Score: 0.0775 (Std: 0.0450)

Accuracy: 0.9047 (Std: 0.0062)

Precision: 0.3852 (Std: 0.2947)

Params:

n_genotype: 89

n_history: 11

n_phenotype: 49

n_behaviour: 18

learning_rate: 1.0102030709795042e-05

epochs: 1640

batch_size: 32

Best Threshold to maximize F1 Score: 0.22

F1 Score: 0.2978 ± 0.0456

Accuracy: 0.8641 ± 0.0117

Precision: 0.2832 ± 0.0464

TSGNN:

Global relief:

Value (Mean AUC): 0.7532256092485068 ± 0.0225

Params:

n_epochs: 2059

lr: 1.185885489910591e-05

weight_decay: 9.916744984817729e-07

batch_size: 64

n_genotype: 125

n_history: 3

n_phenotype: 44

n_behaviour: 20

Best Trial Metrics:

Selected Features: ['rs591058', 'rs1800797', 'rs13946', 'rs25487', 'rs2228570', 'rs12722', 'rs2104772', 'rs1144393', 'rs3196378', 'rs1544410', 'rs2237352', 'rs1137101', 'rs10263021', 'rs4454832', 'rs7528684', 'rs2234693', 'rs10132091', 'rs7035322', 'rs1330363', 'rs1011814', 'rs1249269', 'rs4725069', 'rs820218', 'rs62051384', 'rs11232681', 'rs4701616', 'rs1800629', 'rs11177', 'rs143383', 'rs6617', 'rs6481512', 'rs17756404', 'rs4986938', 'rs13317', 'rs4730153', 'rs911263', 'rs970547', 'rs11225395', 'rs3018362', 'rs11629171', 'rs10992075', 'rs4789932', 'rs2252070', 'rs3789870', 'rs1590', 'rs42531', 'rs11154027', 'rs10759753', 'rs2761884', 'rs4362400', 'rs3219008', 'rs3218791', 'rs17576', 'rs2289360', 'rs1134170', 'rs2525504', 'rs4919510', 'rs1937810', 'rs1718119', 'rs10484958', 'rs2285053', 'class12_SNP_risk_score', 'rs1800972', 'rs4328262', 'rs1800469', 'rs2306033', 'rs9340799', 'rs17583842', 'rs4244032', 'rs1643821', 'rs1800470', 'rs3045', 'rs4903399', 'rs3753841', 'rs2277698', 'rs1800012', 'rs12656106', 'rs187483', 'class1_SNP_risk_score', 'rs2281518', 'sex', 'class123_SNP_risk_score', 'rs1045485', 'rs1554606', 'rs78391032', 'rs2305948', 'rs4654760', 'rs1887632', 'rs1800795', 'rs1021188', 'rs72758637', 'rs12154667', 'rs1138545', 'rs3751143', 'rs1676303',

'rs7021589', 'rs12574452', 'rs13107325', 'rs1548456', 'rs2277268', 'rs912336', 'rs35360670', 'rs420257', 'rs74544784', 'rs60713544', 'rs12429486', 'rs42517', 'rs2010963', 'rs71404070', 'rs117544024', 'rs2586488', 'rs25489', 'rs413826', 'rs650108', 'rs57104447', 'rs42522', 'rs710079', 'rs761804508', 'rs145648292', 'rs77569527', 'rs144371252', 'rs4988321', 'rs149047058', 'rs144414988', 'rs2858056', 'Age', 'tracking_period_injury', 'Athlete_Score', 'Q_angle', 'hip_abduction_peak_torque_asymmetry', 'total_ad_ab_ratio', 'Step_frequency_10', 'thigh_ffmi', 'hip_adduction_peak_torque_asymmetry', 'BMD_hip', 'calf_size', 'Flight_time_12', 'total_fl_ex_ratio', 'navicular_drop', 'Step_frequency_12', 'knee_extension_peak_angle_asymmetry', 'VALR_10', 'VALR_12', 'Q_angle_asymmetry', 'VILR_12', 'VILR_10', 'hip_adduction_peak_torque', 'thigh_lean_mass', 'total_lean_mass', 'leg_ffmi', 'BMD_spine', 'knee_flexion_peak_torque', 'total_ffmi', 'Flight_time_10', 'knee_extension_peak_torque', 'knee_flexion_peak_torque_asymmetry', 'leg_lean_mass', 'hip_abduction_peak_torque', 'VALR_asymmetry_12', 'knee_flexion_peak_angle_asymmetry', 'Impact_peak_12', 'VILR_asymmetry_12', 'BMD_body', 'BMI', 'knee_flexion_peak_angle', 'hip_adduction_peak_angle', 'Duty_factor_10', 'knee_extension_peak_torque_asymmetry', 'lower_leg_lean_mass', 'Duty_factor_asymmetry_12', 'Duty_factor_asymmetry_10', 'Impact_peak_10', 'fat_intake_BW', 'fat_percentage_avg', 'fat_intake_avg', 'glycine_intake_BW', 'arginine_intake_BW', 'calcium_intake_BW', 'average_energy_availability', 'protein_intake_BW', 'SC_past_season', 'SC_past_month', 'vitaminD_intake_BW', 'resistance_training_past_season', 'past_month_volume_low', 'copper_intake_BW', 'past_month_distance', 'drills_past_season', 'non_running_past_season', 'past_month_min', 'bodyweight_exercises_past_season', 'non_running_past_month']

Average AUC: 0.7532 ± 0.0225

Average F1 Score: 0.2214 ± 0.0522

Average Accuracy: 0.9073 ± 0.0063

Average Precision: 0.4877 ± 0.1276

Optimal Threshold: 0.1899

AUPRC: 0.2876 ± 0.0519

F1 Score: 0.3282 ± 0.0515

Accuracy: 0.8403 ± 0.0183

Precision: 0.2681 ± 0.0461

Sensitivity (Recall): 0.4253 ± 0.0611

Specificity: 0.8819 ± 0.0171

Separate relief:

Best trial:

Value (Mean AUC): 0.7530759131312909

Params:

n_epochs: 730

lr: 4.86593416133986e-05

weight_decay: 0.0003438008978072548

batch_size: 256

n_genotype: 126

n_history: 10

n_phenotype: 32

n_behaviour: 9

Best Trial Metrics:

Selected Features: ['rs591058', 'rs2104772', 'rs25487', 'rs1249269', 'rs1800797', 'rs1137101', 'rs1330363', 'rs1144393', 'rs7528684', 'rs4701616', 'rs10132091', 'rs2237352', 'rs143383', 'rs4789932', 'rs2228570', 'rs13946', 'rs10263021', 'rs6481512', 'rs2234693', 'rs6617', 'rs4730153', 'rs7035322', 'rs11177', 'rs12722', 'rs820218', 'rs17756404', 'rs3018362', 'rs4725069', 'rs13317', 'rs4454832', 'rs3196378', 'rs1544410', 'rs62051384', 'rs970547', 'rs4986938', 'rs42531', 'rs17576', 'rs11232681', 'rs911263', 'rs10992075', 'rs1011814', 'rs2252070', 'rs11225395',

'rs11154027', 'rs1800629', 'rs1590', 'rs2761884', 'rs1800469', 'rs4362400', 'rs2525504', 'rs4903399', 'rs3219008', 'rs1800972', 'rs1937810', 'rs3753841', 'rs4919510', 'rs10759753', 'rs3045', 'rs4328262', 'rs1718119', 'rs2306033', 'rs3789870', 'rs4244032', 'rs11629171', 'rs9340799', 'rs2285053', 'rs2289360', 'rs3218791', 'rs17583842', 'rs1800470', 'rs2281518', 'rs10484958', 'class12_SNP_risk_score', 'rs1643821', 'rs12656106', 'sex', 'rs2277698', 'rs1134170', 'class1_SNP_risk_score', 'rs187483', 'rs1800012', 'rs1045485', 'rs1554606', 'class123_SNP_risk_score', 'rs72758637', 'rs1887632', 'rs2305948', 'rs1676303', 'rs1800795', 'rs12154667', 'rs4654760', 'rs1138545', 'rs1021188', 'rs78391032', 'rs3751143', 'rs7021589', 'rs1548456', 'rs2277268', 'rs13107325', 'rs12574452', 'rs912336', 'rs420257', 'rs35360670', 'rs42517', 'rs2010963', 'rs2586488', 'rs60713544', 'rs74544784', 'rs71404070', 'rs12429486', 'rs25489', 'rs117544024', 'rs650108', 'rs761804508', 'rs57104447', 'rs145648292', 'rs42522', 'rs710079', 'rs413826', 'rs4988321', 'rs77569527', 'rs144371252', 'rs144414988', 'rs149047058', 'rs2858056', 'rs3216902', 'Age', 'Athlete_Score', 'average_run_hours', 'average_run_frequency', 'average_interval_training_frequency', 'EDEQ_total', 'LEAF-Q', 'tracking_period_injury', 'lower_limb_days_total', 'past_stress_injury', 'Duty_factor_10', 'Flight_time_10', 'Step_frequency_10', 'Contact_time_10', 'Q_angle_asymmetry', 'Impact_peak_10', 'Cadence_asymmetry_10', 'total_ad_ab_ratio', 'knee_extension_peak_torque', 'hip_abduction_peak_torque', 'hip_adduction_peak_torque', 'Impact_peak_12', 'Duty_factor_12', 'Q_angle', 'Flight_time_12', 'BMI', 'BMD_body', 'knee_flexion_peak_angle', 'knee_flexion_peak_angle_asymmetry', 'hip_adduction_peak_angle', 'Contact_time_12', 'knee_flexion_peak_torque', 'hip_abduction_peak_torque_asymmetry', 'Impact_peak_asymmetry_10', 'knee_flexion_peak_torque_asymmetry', 'Duty_factor_asymmetry_10', 'Cadence_asymmetry_12', 'knee_extension_peak_angle_asymmetry', 'knee_extension_peak_torque_asymmetry', 'knee_extension_peak_angle', 'BMD_hip', 'height', 'fat_intake_BW', 'fat_percentage_avg', 'fat_intake_avg', 'arginine_intake_BW', 'protein_intake_BW', 'average_energy_availability', 'glycine_intake_BW', 'calcium_intake_BW', 'past_month_distance']

Average AUC: 0.7531 ± 0.0378

Average F1 Score: 0.0925 ± 0.0424

Average Accuracy: 0.9068 ± 0.0033

Average Precision: 0.4604 ± 0.2347

Average F1 Score: 0.3619 ± 0.0468

Average Accuracy: 0.8562 ± 0.0304

Average Precision: 0.3162 ± 0.0572

Global lasso:

Best trial:

Value (Mean AUC): 0.7503470913074771

Params:

n_epochs: 1142

lr: 3.5784877937744905e-05

weight_decay: 8.830400761450904e-08

batch_size: 128

n_genotype: 54

n_history: 6

n_phenotype: 12

n_behaviour: 7

Best Trial Metrics:

Selected Features: ['rs7035322', 'rs145648292', 'rs1330363', 'rs144414988', 'rs9340799', 'rs1676303', 'rs2277268', 'rs1011814', 'rs4903399', 'rs1590', 'rs4986938', 'rs4919510', 'rs149047058', 'rs2289360', 'rs1800469', 'rs1249269', 'rs2281518', 'rs2586488', 'rs4454832', 'rs42522', 'rs3216902', 'rs2858056', 'rs591058', 'rs4362400', 'rs10132091', 'rs1937810', 'rs2306033', 'rs1643821', 'rs10484958', 'rs1045485', 'rs42517', 'rs4701616', 'rs2525504', 'rs761804508', 'rs1134170', 'rs11225395', 'rs10759753', 'sex', 'rs1800629', 'rs1144393', 'rs3045', 'rs17583842', 'rs1800972', 'rs4328262', 'rs2234693', 'rs3751143', 'rs1800470', 'rs4988321', 'rs2228570', 'rs78391032', 'rs12656106', 'rs60713544', 'rs143383', 'rs62051384', 'EDEQ_total', 'tracking_period_injury', 'average_run_hours', 'Athlete_Score', 'Age',

'average_interval_training_frequency', 'BMD_body', 'BMD_hip', 'Duty_factor_asymmetry_10', 'Q_angle_asymmetry', 'hip_adduction_peak_torque', 'ad_ab_ratio_asymmetry', 'knee_extension_peak_torque_asymmetry', 'knee_extension_peak_angle_asymmetry', 'knee_flexion_peak_torque', 'hip_abduction_peak_angle', 'knee_flexion_peak_angle_asymmetry', 'navicular_drop_asymmetry', 'omega3_intake_BW', 'copper_intake_BW', 'arginine_intake_BW', 'vitaminD_intake_BW', 'past_month_ratio', 'past_week_ratio_calculated_volume', 'calcium_intake_BW']

Average AUC: 0.7503 ± 0.0355

Average F1 Score: 0.0385 ± 0.0357

Average Accuracy: 0.9062 ± 0.0030

Average Precision: 0.2822 ± 0.2255

Average F1 Score: 0.3472 ± 0.0537

Average Accuracy: 0.8403 ± 0.0495

Average Precision: 0.3085 ± 0.0972

Separate lasso:

Best trial:

Value (Mean AUC): 0.7415440699513642

Params:

n_epochs: 2163

lr: 4.600641941225531e-06

weight_decay: 3.128639842545911e-08

batch_size: 256

n_genotype: 67

n_history: 4

n_phenotype: 47

n_behaviour: 37

Best Trial Metrics:

Selected Features: ['rs144414988', 'rs117544024', 'rs77569527', 'rs78391032', 'rs12722', 'rs3196378', 'rs57104447', 'rs144371252', 'class123_SNP_risk_score', 'rs11177', 'rs2277268', 'rs6617', 'rs4988321', 'rs1676303', 'rs25487', 'rs11154027', 'rs35360670', 'rs6481512', 'rs710079', 'class12_SNP_risk_score', 'rs420257', 'rs42522', 'rs71404070', 'rs1249269', 'rs591058', 'rs72758637', 'rs413826', 'rs74544784', 'rs1330363', 'rs25489', 'rs149047058', 'rs3218791', 'rs4903399', 'rs10263021', 'rs761804508', 'rs2234693', 'rs7528684', 'rs17576', 'rs12154667', 'rs2858056', 'rs17583842', 'rs1137101', 'rs2289360', 'rs2525504', 'rs4701616', 'rs1554606', 'rs1887632', 'rs820218', 'rs2252070', 'rs143383', 'rs1011814', 'rs2586488', 'rs42517', 'rs2277698', 'rs4919510', 'rs3753841', 'rs2104772', 'rs13107325', 'rs2306033', 'rs1800012', 'rs1800972', 'rs11232681', 'rs1800469', 'rs2285053', 'rs12574452', 'rs62051384', 'rs3045', 'tracking_period_injury', 'past_month_injury', 'average_run_hours', 'average_run_frequency', 'Mass', 'BMI', 'VILR_asymmetry_10', 'height', 'VALR_asymmetry_10', 'VALR_10', 'calf_size', 'VILR_10', 'ad_ab_ratio_asymmetry', 'VALR_asymmetry_12', 'Duty_factor_10', 'Impact_peak_12', 'VILR_asymmetry_12', 'Duty_factor_12', 'hip_abduction_peak_torque', 'knee_extension_peak_torque', 'lower_leg_lean_mass', 'knee_extension_peak_angle', 'hip_abduction_peak_torque_asymmetry', 'hip_adduction_peak_torque', 'Step_frequency_12', 'Step_frequency_10', 'hip_adduction_peak_angle_asymmetry', 'knee_flexion_peak_torque', 'Impact_peak_asymmetry_12', 'Impact_peak_10', 'Q_angle', 'total_lean_mass', 'Flight_time_12', 'leg_ffmi', 'total_fl_ex_ratio', 'hip_abduction_peak_angle', 'total_ffmi', 'hip_abduction_peak_angle_asymmetry', 'hip_adduction_peak_torque_asymmetry', 'Alt_strike', 'knee_flexion_peak_angle', 'Q_angle_asymmetry', 'navicular_drop', 'Cadence_asymmetry_10', 'Impact_peak_asymmetry_10', 'Contact_time_10', 'fl_ex_ratio_asymmetry', 'knee_extension_peak_torque_asymmetry', 'total_ad_ab_ratio', 'lower_leg_ffmi', 'Cadence_asymmetry_12', 'past_week_ratio_calculated_volume', 'past_week_ratio', 'arginine_intake_BW', 'glycine_intake_BW', 'stretching_past_season', 'barefoot_past_season', 'past_week_ratio_low', 'circuit_training_past_month', 'past_month_ratio', 'resistance_training_past_season', 'stretching_past_month', 'past_month_distance', 'drills_past_season', 'past_month_ratio_calculated_volume', 'vitaminE_intake_BW', 'past_month_volume_very_high',

'circuit_training_past_season', 'protein_intake_BW', 'iron_intake_BW', 'vitaminD_intake_BW', 'resistance_training_past_month', 'barefoot_past_month', 'past_month_min', 'past_month_volume_low', 'SC_past_month', 'past_week_ratio_moderate', 'vitaminC_intake_BW', 'past_week_ratio_high', 'non_running_past_season', 'fat_percentage_avg', 'non_running_past_month', 'core_stability_past_season', 'calcium_intake_BW', 'omega3_intake_BW', 'past_month_calculated_volume', 'drills_past_month', 'plyometrics_past_season']

Average AUC: 0.7415 ± 0.0366

Average F1 Score: 0.0352 ± 0.0355

Average Accuracy: 0.9068 ± 0.0016

Average Precision: 0.2119 ± 0.1837

Average F1 Score: 0.3479 ± 0.0506

Average Accuracy: 0.8267 ± 0.1040

Average Precision: 0.3057 ± 0.0855

Selected Feature Names

Class 1

Decision Tree

rs12722, rs4986938, rs11225395, rs1144393, rs2252070, rs591058, rs4789932, class1_SNP_risk_score, rs13946, navicular_drop

Random Forest

tracking_period_injury, past_month_distance, Duty_factor_12, Impact_peak_12, Q_angle, average_run_hours, navicular_drop, Q_angle_asymmetry, past_month_ratio, knee_flexion_peak_torque, SC_past_season, rs591058, rs2252070, BMD_spine, class1_SNP_risk_score, lower_limb_days_total, hip_abduction_peak_torque, rs1144393, rs1800012, EDEQ_total, rs9340799, navicular_drop_asymmetry, rs1800795, rs970547, Age, knee_extension_peak_torque, rs13946, rs4789932, rs4986938, non_running_past_season, VALR_12, sex, average_interval_training_frequency, rs650108, rs12722, BMI, fat_intake_avg

SVM

rs12722, rs4986938, rs11225395, rs1144393, rs2252070, rs591058, rs4789932, class1_SNP_risk_score, rs13946, navicular_drop, total_ad_ab_ratio, Q_angle_asymmetry, rs9340799, Q_angle, rs970547, tracking_period_injury, VALR_12, BMI, average_run_hours, Impact_peak_12, hip_abduction_peak_torque, Duty_factor_12, knee_extension_peak_torque, navicular_drop_asymmetry, fat_intake_avg, average_interval_training_frequency, Age, knee_flexion_peak_torque, BMD_spine, rs1800012, EDEQ_total, sex, past_month_distance, rs1800795, SC_past_season, non_running_past_season, lower_limb_days_total

KNN

rs12722, rs4986938, rs11225395, rs1144393, rs2252070, rs591058, rs4789932, class1_SNP_risk_score, rs13946, navicular_drop, total_ad_ab_ratio,

Q_angle_asymmetry, rs9340799, Q_angle, rs970547, tracking_period_injury, VALR_12, BMI, average_run_hours, Impact_peak_12, hip_abduction_peak_torque, Duty_factor_12, knee_extension_peak_torque, navicular_drop_asymmetry, fat_intake_avg, average_interval_training_frequency, Age, knee_flexion_peak_torque, BMD_spine, rs1800012, EDEQ_total, sex, past_month_distance, rs1800795, SC_past_season, non_running_past_season

Naïve Bayes

rs12722, rs4986938, rs11225395, rs1144393, rs2252070, rs591058, rs4789932, class1_SNP_risk_score, rs13946, navicular_drop, total_ad_ab_ratio, Q_angle_asymmetry, rs9340799, Q_angle, rs970547, tracking_period_injury, VALR_12, BMI, average_run_hours, Impact_peak_12, hip_abduction_peak_torque

Adaboost

rs12722, rs4986938, rs11225395, rs1144393, rs2252070, rs591058, rs4789932, class1_SNP_risk_score, rs13946, navicular_drop, total_ad_ab_ratio, Q_angle_asymmetry, rs9340799, Q_angle, rs970547, tracking_period_injury, VALR_12, BMI, average_run_hours, Impact_peak_12, hip_abduction_peak_torque, Duty_factor_12, knee_extension_peak_torque, navicular_drop_asymmetry

Gradient Boosting

tracking_period_injury, past_month_distance, Duty_factor_12, Impact_peak_12, Q_angle, average_run_hours, navicular_drop, Q_angle_asymmetry, past_month_ratio, knee_flexion_peak_torque, SC_past_season, rs591058, rs2252070, BMD_spine, class1_SNP_risk_score, lower_limb_days_total, hip_abduction_peak_torque, rs1144393, rs1800012, EDEQ_total, rs9340799, navicular_drop_asymmetry, rs1800795, rs970547, Age, knee_extension_peak_torque, rs13946

MLP

rs12722, rs4986938, rs11225395, rs1144393, rs2252070, rs591058, rs4789932, class1_SNP_risk_score, rs13946, navicular_drop, total_ad_ab_ratio, Q_angle_asymmetry, rs9340799, Q_angle, rs970547, tracking_period_injury, VALR_12, BMI, average_run_hours, Impact_peak_12, hip_abduction_peak_torque, Duty_factor_12, knee_extension_peak_torque, navicular_drop_asymmetry, fat_intake_avg, average_interval_training_frequency, Age, knee_flexion_peak_torque, BMD_spine, rs1800012, EDEQ_total, sex, past_month_distance, rs1800795, SC_past_season, non_running_past_season

Bayesian Network

tracking_period_injury, rs4986938, rs591058, knee_flexion_peak_torque, average_run_hours, Q_angle, Q_angle_asymmetry, knee_extension_peak_torque, lower_limb_days_total, navicular_drop, total_ad_ab_ratio, hip_abduction_peak_torque,

rs650108, rs11225395, rs13946, past_month_distance, rs9340799, BMD_spine, Impact_peak_12

Logistic Regression

tracking_period_injury, class1_SNP_risk_score, past_month_distance, Duty_factor_12, Impact_peak_12, Q_angle, average_run_hours, navicular_drop, Q_angle_asymmetry, past_month_ratio, knee_flexion_peak_torque, SC_past_season, rs1144393, rs9340799, BMD_spine, lower_limb_days_total, rs1800795, rs4789932, rs591058, hip_abduction_peak_torque, rs4986938

TSNN

'rs2252070', 'rs4986938', 'rs12722', 'rs1144393', 'rs591058', 'rs4789932', 'rs11225395', 'rs13946', 'class1_SNP_risk_score', 'rs9340799', 'rs970547', 'rs1800012', 'sex', 'average_run_hours', 'Age', 'average_interval_training_frequency', 'BMD_spine', 'Q_angle_asymmetry', 'Q_angle', 'Impact_peak_12', 'navicular_drop', 'Duty_factor_12', 'VALR_12', 'knee_flexion_peak_torque', 'navicular_drop_asymmetry', 'total_ad_ab_ratio', 'hip_abduction_peak_torque', 'fat_intake_avg', 'past_month_distance', 'SC_past_season'

TSGNN

'rs12722', 'rs4986938', 'rs11225395', 'rs1144393', 'rs2252070', 'rs591058', 'rs4789932', 'class1_SNP_risk_score', 'rs13946', 'rs9340799', 'rs970547', 'rs1800012',

'tracking_period_injury', 'average_run_hours', 'average_interval_training_frequency', 'Age', 'EDEQ_total', 'navicular_drop', 'total_ad_ab_ratio', 'Q_angle_asymmetry', 'Q_angle', 'VALR_12', 'BMI', 'Impact_peak_12', 'hip_abduction_peak_torque', 'Duty_factor_12', 'knee_extension_peak_torque', 'navicular_drop_asymmetry', 'fat_intake_avg', 'past_month_distance', 'SC_past_season', 'non_running_past_season'

Class 1-3

Decision Tree

rs591058, rs1800797, rs13946, rs25487, rs2228570, rs12722, rs2104772, rs1144393, rs3196378, rs1544410, rs2237352, rs1137101, rs10263021, rs4454832, rs7528684, rs2234693, rs10132091, rs7035322, rs1330363, rs1011814, rs1249269, rs4725069, rs820218, rs62051384, rs11232681, rs4701616, rs1800629, rs11177, rs143383, rs6617, rs6481512, rs17756404, rs4986938, rs13317, rs4730153, rs911263, rs970547, rs11225395, rs3018362, rs11629171, rs10992075, rs4789932, rs2252070, rs3789870, rs1590, rs42531, rs11154027, rs10759753, rs2761884, rs4362400, Q_angle, rs3219008, hip_abduction_peak_torque_asymmetry, rs3218791, total_ad_ab_ratio, rs17576, rs2289360, Age, rs1134170, rs2525504

Random Forest

rs591058, rs1800797, rs13946, rs25487, rs2228570, rs12722, rs2104772, rs1144393, rs3196378, rs1544410, rs2237352, rs1137101, rs10263021, rs4454832, rs7528684, rs2234693, rs10132091, rs7035322, rs1330363, rs1011814, rs1249269, rs4725069, rs820218, rs62051384, rs11232681, rs4701616, rs1800629, rs11177, rs143383, rs6617, rs6481512, rs17756404, rs4986938, rs13317, rs4730153, rs911263, rs970547, rs11225395, rs3018362, rs11629171, rs10992075, rs4789932, rs2252070, rs3789870, rs1590, rs42531, rs11154027, rs10759753, rs2761884, rs4362400, Q_angle, rs3219008, hip_abduction_peak_torque_asymmetry, rs3218791, total_ad_ab_ratio, rs17576, rs2289360, Age, rs1134170, rs2525504, fat_intake_BW, rs4919510, tracking_period_injury, rs1937810, Step_frequency_10, Athlete_Score, rs1718119, rs10484958, rs2285053, thigh_ffmi, class12_SNP_risk_score, fat_percentage_avg, hip_adduction_peak_torque_asymmetry, rs1800972, rs4328262, BMD_hip, calf_size, Flight_time_12, total_fl_ex_ratio, rs1800469, fat_intake_avg, navicular_drop, Step_frequency_12, knee_extension_peak_angle_asymmetry, glycine_intake_BW, rs2306033, rs9340799, rs17583842, rs4244032, rs1643821, rs1800470, rs3045, arginine_intake_BW, VALR_10, average_run_frequency, VALR_12, Q_angle_asymmetry, VILR_12, VILR_10, hip_adduction_peak_torque, rs4903399, thigh_lean_mass, rs3753841, calcium_intake_BW, total_lean_mass, leg_ffmi, rs2277698, BMD_spine, rs1800012, knee_flexion_peak_torque, total_ffmi, Flight_time_10, knee_extension_peak_torque, knee_flexion_peak_torque_asymmetry, average_run_hours, leg_lean_mass, average_interval_training_frequency, hip_abduction_peak_torque, rs12656106, average_energy_availability,

VALR_asymmetry_12, knee_flexion_peak_angle_asymmetry, Impact_peak_12, VILR_asymmetry_12, BMD_body, BMI, knee_flexion_peak_angle, hip_adduction_peak_angle, Duty_factor_10, knee_extension_peak_torque_asymmetry, rs187483, lower_leg_lean_mass, class1_SNP_risk_score, Duty_factor_asymmetry_12, Duty_factor_asymmetry_10, Impact_peak_10, Duty_factor_12, height, rs2281518, protein_intake_BW, navicular_drop_asymmetry, Cadence_asymmetry_10, past_month_injury, Mass, Impact_peak_asymmetry_10, sex, lower_leg_ffmi, EDEQ_total, class123_SNP_risk_score, rs1045485, rs1554606, knee_extension_peak_angle, fl_ex_ratio_asymmetry, rs78391032, past_stress_injury, Cadence_asymmetry_12, rs2305948, rs4654760, Contact_time_10, VALR_asymmetry_10, rs1887632, rs1800795, rs1021188, rs72758637, VILR_asymmetry_10, rs12154667, Contact_time_12, SC_past_season, rs1138545, rs3751143, rs1676303, rs7021589, SC_past_month, rs12574452, LEAF-Q, ad_ab_ratio_asymmetry, Impact_peak_asymmetry_12, vitaminD_intake_BW, rs13107325, rs1548456, rs2277268, resistance_training_past_season, past_month_volume_low, copper_intake_BW, past_month_distance, rs912336, hip_adduction_peak_angle_asymmetry, hip_abduction_peak_angle_asymmetry, hip_abduction_peak_angle, drills_past_season, non_running_past_season, past_month_min, rs35360670, rs420257, bodyweight_exercises_past_season, lower_limb_days_total, rs74544784, non_running_past_month, rs60713544, rs12429486, resistance_training_past_month, rs42517, rs2010963, rs71404070, core_stability_past_season, vitaminC_intake_BW, rs117544024, bodyweight_exercises_past_month

SVM

BMD_body, rs7035322, rs145648292, BMD_hip, omega3_intake_BW, Duty_factor_asymmetry_10, rs1676303, rs144414988, average_run_hours, ad_ab_ratio_asymmetry, hip_adduction_peak_torque, tracking_period_injury, Q_angle_asymmetry, past_month_ratio, rs1330363, rs9340799, knee_extension_peak_angle_asymmetry, rs2277268, copper_intake_BW, vitaminD_intake_BW, arginine_intake_BW, rs2858056, calcium_intake_BW, rs1011814, rs4903399, EDEQ_total, rs1590, rs4986938, Contact_time_12, rs3045, knee_flexion_peak_angle_asymmetry, rs4919510, rs1800469, rs1249269, Athlete_Score, rs11225395, rs10759753, rs2289360, SC_past_season, rs4701616, resistance_training_past_season, fat_percentage_avg

KNN

BMD_body, rs7035322, rs145648292, BMD_hip, omega3_intake_BW, Duty_factor_asymmetry_10, rs144414988, rs1676303, hip_adduction_peak_torque

Naïve Bayes

rs591058, rs1800797, rs13946, rs25487, rs2228570, rs12722, rs2104772, rs1144393, rs3196378, rs1544410, rs2237352, rs1137101, rs10263021, rs4454832, rs7528684, rs2234693, rs10132091, rs7035322, rs1330363, rs1011814, rs1249269, rs4725069,

rs820218, rs62051384, rs11232681, rs4701616, rs1800629, rs11177, rs143383, rs6617, rs6481512, rs17756404, rs4986938, rs13317, rs4730153, rs911263, rs970547, rs11225395, rs3018362, rs11629171, rs10992075, rs4789932, rs2252070, rs3789870, rs1590, rs42531, rs11154027, rs10759753, rs2761884, rs4362400, Q_angle, rs3219008, hip_abduction_peak_torque_asymmetry, rs3218791, total_ad_ab_ratio, rs17576, rs2289360, Age, rs1134170, rs2525504, fat_intake_BW, rs4919510, tracking_period_injury, rs1937810, Step_frequency_10, Athlete_Score, rs1718119, rs10484958, rs2285053, thigh_ffmi, class12_SNP_risk_score, fat_percentage_avg, hip_adduction_peak_torque_asymmetry, rs1800972, rs4328262, BMD_hip, calf_size, Flight_time_12, total_fl_ex_ratio, rs1800469, fat_intake_avg, navicular_drop, Step_frequency_12, knee_extension_peak_angle_asymmetry, glycine_intake_BW, rs2306033, rs9340799, rs17583842, rs4244032, rs1643821, rs1800470, rs3045, arginine_intake_BW, VALR_10, average_run_frequency, VALR_12, Q_angle_asymmetry, VILR_12, VILR_10, hip_adduction_peak_torque, rs4903399, thigh_lean_mass, rs3753841, calcium_intake_BW, total_lean_mass, leg_ffmi, rs2277698, BMD_spine, rs1800012, knee_flexion_peak_torque, total_ffmi, Flight_time_10, knee_extension_peak_torque, knee_flexion_peak_torque_asymmetry, average_run_hours, leg_lean_mass, average_interval_training_frequency, hip_abduction_peak_torque, rs12656106, average_energy_availability, VALR_asymmetry_12, knee_flexion_peak_angle_asymmetry, Impact_peak_12, VILR_asymmetry_12, BMD_body, BMI, knee_flexion_peak_angle, hip_adduction_peak_angle, Duty_factor_10, knee_extension_peak_torque_asymmetry, rs187483, lower_leg_lean_mass, class1_SNP_risk_score, Duty_factor_asymmetry_12,

Duty_factor_asymmetry_10, Impact_peak_10, Duty_factor_12, height, rs2281518, protein_intake_BW, navicular_drop_asymmetry, Cadence_asymmetry_10, past_month_injury, Mass, Impact_peak_asymmetry_10, sex, lower_leg_ffmi, EDEQ_total, class123_SNP_risk_score, rs1045485, rs1554606, knee_extension_peak_angle, fl_ex_ratio_asymmetry, rs78391032, past_stress_injury

Adaboost

rs591058, rs1800797, rs13946, rs25487, rs2228570, rs12722, rs2104772, rs1144393, rs3196378, rs1544410, rs2237352, rs1137101, rs10263021, rs4454832, rs7528684, rs2234693, rs10132091, rs7035322, rs1330363, rs1011814, rs1249269, rs4725069, rs820218, rs62051384, rs11232681, rs4701616, rs1800629, rs11177, rs143383, rs6617, rs6481512, rs17756404, rs4986938, rs13317, rs4730153, rs911263, rs970547, rs11225395, rs3018362, rs11629171, rs10992075, rs4789932, rs2252070, rs3789870, rs1590, rs42531, rs11154027, rs10759753, rs2761884, rs4362400, Q_angle, rs3219008, hip_abduction_peak_torque_asymmetry, rs3218791, total_ad_ab_ratio, rs17576, rs2289360, Age, rs1134170, rs2525504, fat_intake_BW, rs4919510, tracking_period_injury, rs1937810, Step_frequency_10, Athlete_Score, rs1718119, rs10484958, rs2285053, thigh_ffmi, class12_SNP_risk_score, fat_percentage_avg, hip_adduction_peak_torque_asymmetry, rs1800972, rs4328262, BMD_hip, calf_size, Flight_time_12, total_fl_ex_ratio, rs1800469, fat_intake_avg, navicular_drop, Step_frequency_12, knee_extension_peak_angle_asymmetry, glycine_intake_BW, rs2306033, rs9340799, rs17583842, rs4244032, rs1643821, rs1800470, rs3045, arginine_intake_BW, VALR_10, average_run_frequency, VALR_12,

Q_angle_asymmetry, VILR_12, VILR_10, hip_adduction_peak_torque, rs4903399, thigh_lean_mass, rs3753841, calcium_intake_BW, total_lean_mass, leg_ffmi, rs2277698, BMD_spine, rs1800012, knee_flexion_peak_torque, total_ffmi, Flight_time_10, knee_extension_peak_torque, knee_flexion_peak_torque_asymmetry, average_run_hours, leg_lean_mass, average_interval_training_frequency, hip_abduction_peak_torque, rs12656106, average_energy_availability, VALR_asymmetry_12, knee_flexion_peak_angle_asymmetry, Impact_peak_12, VILR_asymmetry_12, BMD_body, BMI, knee_flexion_peak_angle, hip_adduction_peak_angle, Duty_factor_10, knee_extension_peak_torque_asymmetry, rs187483, lower_leg_lean_mass, class1_SNP_risk_score, Duty_factor_asymmetry_12, Duty_factor_asymmetry_10, Impact_peak_10, Duty_factor_12, height, rs2281518, protein_intake_BW, navicular_drop_asymmetry, Cadence_asymmetry_10, past_month_injury, Mass, Impact_peak_asymmetry_10, sex, lower_leg_ffmi, EDEQ_total, class123_SNP_risk_score, rs1045485, rs1554606, knee_extension_peak_angle, fl_ex_ratio_asymmetry, rs78391032, past_stress_injury, Cadence_asymmetry_12, rs2305948, rs4654760, Contact_time_10

Gradient Boosting

BMD_body, rs7035322, rs145648292, BMD_hip, omega3_intake_BW, Duty_factor_asymmetry_10, rs144414988, rs1676303, hip_adduction_peak_torque, Q_angle_asymmetry, ad_ab_ratio_asymmetry, average_run_hours

MLP

rs591058, rs1800797, rs13946, rs25487, rs2228570, rs12722, rs2104772, rs1144393, rs3196378, rs1544410, rs2237352, rs1137101, rs10263021, rs4454832, rs7528684, rs2234693, rs10132091, rs7035322, rs1330363, rs1011814, rs1249269, rs4725069, rs820218, rs62051384, rs11232681, rs4701616, rs1800629, rs11177, rs143383, rs6617, rs6481512, rs17756404, rs4986938, rs13317, rs4730153, rs911263, rs970547, rs11225395, rs3018362, rs11629171, rs10992075, rs4789932, rs2252070, rs3789870, rs1590, rs42531, rs11154027, rs10759753, rs2761884, rs4362400, Q_angle, rs3219008, hip_abduction_peak_torque_asymmetry, rs3218791, total_ad_ab_ratio, rs17576, rs2289360, Age, rs1134170, rs2525504, fat_intake_BW, rs4919510, tracking_period_injury, rs1937810, Step_frequency_10, Athlete_Score, rs1718119, rs10484958, rs2285053, thigh_ffmi, class12_SNP_risk_score, fat_percentage_avg, hip_adduction_peak_torque_asymmetry, rs1800972, rs4328262, BMD_hip, calf_size, Flight_time_12, total_fl_ex_ratio, rs1800469, fat_intake_avg, navicular_drop, Step_frequency_12, knee_extension_peak_angle_asymmetry, glycine_intake_BW, rs2306033, rs9340799, rs17583842, rs4244032, rs1643821, rs1800470, rs3045, arginine_intake_BW, VALR_10, average_run_frequency, VALR_12, Q_angle_asymmetry, VILR_12, VILR_10, hip_adduction_peak_torque, rs4903399, thigh_lean_mass, rs3753841, calcium_intake_BW, total_lean_mass, leg_ffmi, rs2277698, BMD_spine, rs1800012, knee_flexion_peak_torque, total_ffmi, Flight_time_10, knee_extension_peak_torque, knee_flexion_peak_torque_asymmetry, average_run_hours, leg_lean_mass, average_interval_training_frequency, hip_abduction_peak_torque, rs12656106, average_energy_availability,

VALR_asymmetry_12, knee_flexion_peak_angle_asymmetry, Impact_peak_12, VILR_asymmetry_12, BMD_body, BMI, knee_flexion_peak_angle, hip_adduction_peak_angle, Duty_factor_10, knee_extension_peak_torque_asymmetry, rs187483, lower_leg_lean_mass, class1_SNP_risk_score, Duty_factor_asymmetry_12, Duty_factor_asymmetry_10, Impact_peak_10, Duty_factor_12, height, rs2281518, protein_intake_BW, navicular_drop_asymmetry, Cadence_asymmetry_10, past_month_injury, Mass

Bayesian Network

rs2252070, Impact_peak_12, navicular_drop, EDEQ_total, navicular_drop_asymmetry, rs13946, Q_angle, average_run_hours, class1_SNP_risk_score, rs9340799, lower_limb_days_total, Duty_factor_12, VALR_12, Q_angle_asymmetry, average_interval_training_frequency, total_ad_ab_ratio, hip_abduction_peak_torque, tracking_period_injury

Logistic Regression

rs145648292, average_run_hours, rs1676303, BMD_body, knee_extension_peak_angle_asymmetry, past_month_ratio, rs2277268, SC_past_season, rs3045, past_week_ratio_low, tracking_period_injury, rs7035322, Q_angle_asymmetry, Duty_factor_asymmetry_10, vitaminD_intake_BW, rs4988321, ad_ab_ratio_asymmetry, rs1144393, class1_SNP_risk_score, past_month_ratio_calculated_volume, rs4701616, rs13107325, calcium_intake_BW, rs1590, arginine_intake_BW, SC_past_month,

rs11225395, hip_abduction_peak_angle, rs2104772, BMD_hip, resistance_training_past_season, rs2306033, hip_adduction_peak_torque, rs25489, rs4919510, rs10484958, fat_percentage_avg, leg_ffmi, rs912336, rs1800629, rs2289360, knee_flexion_peak_angle_asymmetry, rs2858056, average_energy_availability, rs10132091, rs2586488, resistance_training_past_month, Contact_time_12, rs1011814, past_month_distance, rs1249269, rs4903399, Alt_strike, rs1643821, rs1800972, rs1800469, rs2281518, rs11232681, rs62051384, rs3218791, rs1330363, rs2305948, Age, rs3219008, rs2010963, Athlete_Score, fl_ex_ratio_asymmetry, navicular_drop_asymmetry, past_month_injury, rs2252070, rs1800795, copper_intake_BW, protein_intake_BW, rs9340799, rs2234693, past_month_volume_high, rs25487, rs1045485, past_week_ratio_calculated_volume, rs2285053, rs6481512, rs10992075, rs143383, rs4328262, rs1887632, rs4986938, iron_intake_BW, drills_past_season, past_week_ratio_high, rs591058, rs17583842, circuit_training_past_month, rs12574452, past_month_ratio_moderate, EDEQ_total

TSNN

'rs591058', 'rs1800797', 'rs13946', 'rs25487', 'rs2228570', 'rs12722', 'rs2104772', 'rs1144393', 'rs3196378', 'rs1544410', 'rs2237352', 'rs1137101', 'rs10263021', 'rs4454832', 'rs7528684', 'rs2234693', 'rs10132091', 'rs7035322', 'rs1330363', 'rs1011814', 'rs1249269', 'rs4725069', 'rs820218', 'rs62051384', 'rs11232681', 'rs4701616', 'rs1800629', 'rs11177', 'rs143383', 'rs6617', 'rs6481512', 'rs17756404', 'rs4986938', 'rs13317', 'rs4730153', 'rs911263', 'rs970547', 'rs11225395', 'rs3018362', 'rs11629171', 'rs10992075', 'rs4789932', 'rs2252070', 'rs3789870', 'rs1590', 'rs42531', 'rs11154027', 'rs10759753', 'rs2761884',

'rs4362400', 'rs3219008', 'rs3218791', 'rs17576', 'rs2289360', 'rs1134170', 'rs2525504', 'rs4919510', 'rs1937810', 'rs1718119', 'rs10484958', 'rs2285053', 'class12_SNP_risk_score', 'rs1800972', 'rs4328262', 'rs1800469', 'rs2306033', 'rs9340799', 'rs17583842', 'Age', 'Q_angle', 'hip_abduction_peak_torque_asymmetry', 'total_ad_ab_ratio', 'Step_frequency_10', 'thigh_ffmi', 'hip_adduction_peak_torque_asymmetry', 'BMD_hip', 'calf_size', 'Flight_time_12', 'total_fl_ex_ratio', 'navicular_drop', 'Step_frequency_12', 'knee_extension_peak_angle_asymmetry', 'VALR_10', 'VALR_12', 'Q_angle_asymmetry', 'VILR_12', 'VILR_10', 'hip_adduction_peak_torque', 'thigh_lean_mass', 'total_lean_mass', 'leg_ffmi', 'BMD_spine', 'knee_flexion_peak_torque', 'total_ffmi', 'Flight_time_10', 'knee_extension_peak_torque', 'knee_flexion_peak_torque_asymmetry', 'leg_lean_mass', 'hip_abduction_peak_torque', 'VALR_asymmetry_12', 'knee_flexion_peak_angle_asymmetry', 'Impact_peak_12', 'VILR_asymmetry_12', 'BMD_body', 'BMI', 'knee_flexion_peak_angle', 'hip_adduction_peak_angle', 'Duty_factor_10', 'knee_extension_peak_torque_asymmetry', 'lower_leg_lean_mass', 'Duty_factor_asymmetry_12', 'Duty_factor_asymmetry_10', 'Impact_peak_10', 'Duty_factor_12', 'height', 'navicular_drop_asymmetry', 'Cadence_asymmetry_10', 'Mass', 'Impact_peak_asymmetry_10', 'lower_leg_ffmi', 'knee_extension_peak_angle', 'fl_ex_ratio_asymmetry', 'Cadence_asymmetry_12', 'Contact_time_10', 'VALR_asymmetry_10', 'VILR_asymmetry_10', 'Contact_time_12', 'ad_ab_ratio_asymmetry', 'fat_intake_BW', 'fat_percentage_avg', 'fat_intake_avg', 'glycine_intake_BW', 'arginine_intake_BW', 'calcium_intake_BW', 'average_energy_availability'

TSGNN

'rs591058', 'rs1800797', 'rs13946', 'rs25487', 'rs2228570', 'rs12722', 'rs2104772', 'rs1144393', 'rs3196378', 'rs1544410', 'rs2237352', 'rs1137101', 'rs10263021', 'rs4454832', 'rs7528684', 'rs2234693', 'rs10132091', 'rs7035322', 'rs1330363', 'rs1011814', 'rs1249269', 'rs4725069', 'rs820218', 'rs62051384', 'rs11232681', 'rs4701616', 'rs1800629', 'rs11177', 'rs143383', 'rs6617', 'rs6481512', 'rs17756404', 'rs4986938', 'rs13317', 'rs4730153', 'rs911263', 'rs970547', 'rs11225395', 'rs3018362', 'rs11629171', 'rs10992075', 'rs4789932', 'rs2252070', 'rs3789870', 'rs1590', 'rs42531', 'rs11154027', 'rs10759753', 'rs2761884', 'rs4362400', 'rs3219008', 'rs3218791', 'rs17576', 'rs2289360', 'rs1134170', 'rs2525504', 'rs4919510', 'rs1937810', 'rs1718119', 'rs10484958', 'rs2285053', 'class12_SNP_risk_score', 'rs1800972', 'rs4328262', 'rs1800469', 'rs2306033', 'rs9340799', 'rs17583842', 'rs4244032', 'rs1643821', 'rs1800470', 'rs3045', 'rs4903399', 'rs3753841', 'rs2277698', 'rs1800012', 'rs12656106', 'rs187483', 'class1_SNP_risk_score', 'rs2281518', 'sex', 'class123_SNP_risk_score', 'rs1045485', 'rs1554606', 'rs78391032', 'rs2305948', 'rs4654760', 'rs1887632', 'rs1800795', 'rs1021188', 'rs72758637', 'rs12154667', 'rs1138545', 'rs3751143', 'rs1676303', 'rs7021589', 'rs12574452', 'rs13107325', 'rs1548456', 'rs2277268', 'rs912336', 'rs35360670', 'rs420257', 'rs74544784', 'rs60713544', 'rs12429486', 'rs42517', 'rs2010963', 'rs71404070', 'rs117544024', 'rs2586488', 'rs25489', 'rs413826', 'rs650108', 'rs57104447', 'rs42522', 'rs710079', 'rs761804508', 'rs145648292', 'rs77569527', 'rs144371252', 'rs4988321', 'rs149047058', 'rs144414988', 'rs2858056', 'Age', 'tracking_period_injury', 'Athlete_Score', 'Q_angle', 'hip_abduction_peak_torque_asymmetry', 'total_ad_ab_ratio', 'Step_frequency_10',

'thigh_ffmi', 'hip_adduction_peak_torque_asymmetry', 'BMD_hip', 'calf_size', 'Flight_time_12', 'total_fl_ex_ratio', 'navicular_drop', 'Step_frequency_12', 'knee_extension_peak_angle_asymmetry', 'VALR_10', 'VALR_12', 'Q_angle_asymmetry', 'VILR_12', 'VILR_10', 'hip_adduction_peak_torque', 'thigh_lean_mass', 'total_lean_mass', 'leg_ffmi', 'BMD_spine', 'knee_flexion_peak_torque', 'total_ffmi', 'Flight_time_10', 'knee_extension_peak_torque', 'knee_flexion_peak_torque_asymmetry', 'leg_lean_mass', 'hip_abduction_peak_torque', 'VALR_asymmetry_12', 'knee_flexion_peak_angle_asymmetry', 'Impact_peak_12', 'VILR_asymmetry_12', 'BMD_body', 'BMI', 'knee_flexion_peak_angle', 'hip_adduction_peak_angle', 'Duty_factor_10', 'knee_extension_peak_torque_asymmetry', 'lower_leg_lean_mass', 'Duty_factor_asymmetry_12', 'Duty_factor_asymmetry_10', 'Impact_peak_10', 'fat_intake_BW', 'fat_percentage_avg', 'fat_intake_avg', 'glycine_intake_BW', 'arginine_intake_BW', 'calcium_intake_BW', 'average_energy_availability', 'protein_intake_BW', 'SC_past_season', 'SC_past_month', 'vitaminD_intake_BW', 'resistance_training_past_season', 'past_month_volume_low', 'copper_intake_BW', 'past_month_distance', 'drills_past_season', 'non_running_past_season', 'past_month_min', 'bodyweight_exercises_past_season', 'non_running_past_month'
